# Clb3-centered regulations are recurrent across distinct parameter regions in minimal autonomous cell cycle oscillator designs

**DOI:** 10.1038/s41540-020-0125-0

**Published:** 2020-04-03

**Authors:** Thierry D. G. A. Mondeel, Oleksandr Ivanov, Hans V. Westerhoff, Wolfram Liebermeister, Matteo Barberis

**Affiliations:** 1 Systems Biology, School of Biosciences and Medicine, Faculty of Health and Medical Sciences, University of Surrey, Guildford, Surrey UK; 2 Centre for Mathematical and Computational Biology, CMCB, University of Surrey, Guildford, UK; 3 Synthetic Systems Biology and Nuclear Organization, Swammerdam Institute for Life Sciences, University of Amsterdam, Amsterdam, The Netherlands; 4 Theoretical Research in Evolutionary Life Sciences, Groningen Institute for Evolutionary Life Sciences, University of Groningen, Groningen, The Netherlands; 5 Systems, Control and Applied Analysis Group, Johan Bernoulli Institute for Mathematics and Computer Science, University of Groningen, Groningen, The Netherlands; 6 Molecular Cell Physiology, VU University Amsterdam, Amsterdam, The Netherlands; 7 Institute of Biochemistry, Charité Universitätsmedizin Berlin, Berlin, Germany; 8 Université Paris-Saclay, INRAE, MaIAGE, Jouy en Josas, France

**Keywords:** Oscillators, Dynamical systems

## Abstract

Some biological networks exhibit oscillations in their components to convert stimuli to time-dependent responses. The eukaryotic cell cycle is such a case, being governed by waves of cyclin-dependent kinase (cyclin/Cdk) activities that rise and fall with specific timing and guarantee its timely occurrence. Disruption of cyclin/Cdk oscillations could result in dysfunction through reduced cell division. Therefore, it is of interest to capture properties of network designs that exhibit robust oscillations. Here we show that a minimal yeast cell cycle network is able to oscillate autonomously, and that cyclin/Cdk-mediated positive feedback loops (PFLs) and Clb3-centered regulations sustain cyclin/Cdk oscillations, in known and hypothetical network designs. We propose that Clb3-mediated coordination of cyclin/Cdk waves reconciles checkpoint and oscillatory cell cycle models. Considering the evolutionary conservation of the cyclin/Cdk network across eukaryotes, we hypothesize that functional (“healthy”) phenotypes require the capacity to oscillate autonomously whereas dysfunctional (potentially “diseased”) phenotypes may lack this capacity.

## Introduction

Living systems exhibit dynamic self-organization, i.e. the spontaneous emergence of spatio-temporal order with the formation of various spatio-temporal patterns^1^. Self-organization may involve oscillations in the concentrations of a system’s components^2–4^, which have been observed at various temporal scales in cell populations. Oscillatory behavior arises from non-linear interactions among two or more components of a system^5^. An example is given by the eukaryotic cell cycle, the sequential process through which a growing cell replicates and divides into two daughter cells. The dynamics of this process are implemented through biochemical interactions between genes and proteins, and are governed by periodic waves of cyclin-dependent kinase (Cdk) activities^6–10^.

Here, self-organization in the form of oscillations results from the sequential activation and inactivation of a number of cyclin/Cdk complexes that regulate a timely cell cycle^8^. The periodic fluctuations of cyclin/Cdk activities are regulated by cyclin levels (i) through transcription factors and (ii) through targeted degradation by multi-protein complexes such as the anaphase-promoting complex (APC).

Sustained cyclin/Cdk oscillations equate to growth and cell division. For bacteria and single-cell organisms such as budding yeast, (faster) growing subpopulations will outperform slower growing and not-growing subpopulations, thus providing a selectable advantage. Thus, the increased fitness of an organism, realized through sustained, autonomous oscillations, can be considered a functional or “healthy” phenotype of a cell. In contrast, lack of oscillations in cyclin/Cdk complexes is to be considered dysfunctional or “diseased” behavior, unless quiescent cells are considered.

Mathematical modeling can be of help to better understand how cell cycle networks exhibit oscillations with certain properties, e.g. a specific amplitude and/or frequency and a definite order of appearance among a system’s components. Cell cycle oscillations have been modeled (i) by sustained oscillations in the form of autonomous limit cycles, where cyclin/Cdk oscillations arise independently from external factors, or (ii) by checkpoint mechanisms, where external requirements such as attaining a minimum cell size to progress from the G1 to S phase are explicitly taken into account in the form of irreversible transitions between steady-states. Here, checkpoints act as signals that delay the cell cycle phase transitions by stabilizing the dynamics in alternative stable steady states of the underlying biochemical system. Contrarily, sustained autonomous oscillations exhibit limit cycles around a single steady-state. The checkpoint view is currently prevalent, due to correlations observed between the cell cycle period and the growth rate^11^, although noise-induced oscillations have been theoretically predicted when cell size is constant^12^. However, models that exhibit autonomous oscillations in the form of limit cycles are better suited when networks are investigated in the absence of external controls such as cell size.

Among the network designs that have been described to characterize cell cycle oscillators, positive feedback loops (PFLs) enhance amplitude and robustness of cyclin/Cdk oscillations^13–15^. PFLs promote switch-like responses that guarantee unidirectionality of cell cycle progression^5^. Similarly, negative feedback loops (NFLs) with delays can produce oscillations, and combinations of the two can enhance robustness of the oscillations^15,16^, and a model consisting of at least three ordinary differential equations (ODEs) is needed for sustained oscillations to occur^5^. It has been conjectured that PFLs have evolved to facilitate oscillations in NFLs at lower, kinetically achievable, degrees of cooperativity^5^. Alteration in the frequency of cyclin/Cdk oscillations or of a cell cycle as a whole may correspond to alteration of cell proliferation, thereby to a dysfunctional or “diseased” phenotype of a cell, as a result of deregulation of timely cyclin/Cdk activities^9,17,18^. This deregulation may impinge on the cellular concentrations and properties of cyclin and Cdk proteins, which already exhibit significant oscillations in a wild-type cell^19^.

Here we build on our previously published minimal model of the cell cycle network^10^ to generate a truly autonomously oscillating model of Clb/Cdk1 complexes in budding yeast, with the intent to: (i) simplify our previously published model to make it more amenable to the parameter scans performed in this work, (ii) integrate new evidence in order for the model to accurately reflect the experimental observations, and (iii) investigate the effect of hypothetical interactions that can be validated experimentally. For each of the 11 resulting network designs we investigate (i) which network designs exhibit autonomous, stable limit cycle oscillations, and (ii) how network designs and associated parameters influence the occurrence and frequency of these oscillations.

The design described by our model(s) comprehends: (i) three cyclin/Cdk complexes, i.e. Clb5,6/Cdk1, Clb3,4/Cdk1, and Clb1,2/Cdk1, which exert their function in the S–G2–M (mitotic) phases of the cell cycle, and (ii) their stoichiometric inhibitor Sic1 that is active in the G1 phase. We consider all the interactions among the three Clb/Cdk1 complexes and Sic1 that are reported in literature. One key feature of our model design is the incorporation of the Clb3 cyclin, which is lacking in existing cell cycle models^20,21^. We investigated whether the experimentally characterized properties of this minimal network would allow for autonomous oscillations to occur. Our analysis is driven by the hypothesis that some elements of the experimentally characterized interaction network are critical, or more important, than others to generate sustained oscillations. Dynamic models based on this design exhibit transient oscillations of all mitotic cyclins simultaneously^10^, and thereby of cyclin/Cdk activities, which may result in a frequency characteristic of a functional wild type cell. However, so far no analysis has been conducted to investigate: (i) whether these models are actually able to oscillate autonomously, and (ii) how the occurrence and properties of cyclin/Cdk oscillations can be modulated by variation of model parameters, suggesting shifts to dysfunctional or hyperfunctional states.

Other models of the cell cycle have been investigated in terms of their potential to show oscillations (briefly called “oscillatory potential” below)^8,20,21^; however, these are: (i) larger in size, (ii) different in the network structure, and (iii) analyzed only using bifurcation analysis techniques to find single oscillating points or regions in the parameter space. Due to the differences in network design and model size, it is not clear a priori that the results from existing models would translate to the simplified network investigated here.

Identifying limit cycles is an open mathematical challenge for high-dimensional systems, and even numerically this is challenging. This problem is exacerbated if the interest is to find multiple limit cycles across distinct regions in the parameter space. Several methods exist for finding parameter sets leading to bifurcations and oscillations in biochemical networks^22–24^. In this work, we make use of the System Design Space (SDS) methodology^25–27^ to detect limit cycles more easily^28^, and analyze the ability of our minimal cell cycle model to generate sustained oscillations. The application of the SDS methodology to analyze oscillatory behavior is new in the cell cycle field, and it has never been applied to models of the size considered here. Our pipeline centered around the SDS method allows to search for oscillations across a set of regions that partition the parameter space, each with unique network properties. The SDS methodology relates genotype and environment, which affect biochemical and environmental parameters in the system, to the phenotype of steady-state attributes of the biochemical system. It does this by deconstructing the biochemical system into a finite number of qualitatively distinct subsystems. The analogy is that the genotype and environment set the specific parameter values in the system which, when altered, generate differences in phenotypic characteristic such as steady-state stability. Within this approach, the term “phenotype” refers to a combination of “dominant terms”, i.e. a subset of interactions in the network that are large in numerical value with respect to the other terms, which are then neglected from the equations. Note that the parameters (genotype and environment) and dynamic concentrations in the system define which terms are dominant (numerically large) and therefore which phenotype is expressed.

The computational cost of using the SDS methodology increases with the number of terms in the model equations, since this number translates into more distinct phenotypes. For this reason, it is advantageous to use our previously published model, which has significantly fewer terms than other published models for budding yeast^20,21,29^. Applying the pipeline considered here to the more complex yeast cell cycle models would most likely require significant computer cluster usage. The disadvantage of using a minimal model is that biochemical details that have been uncovered about the cell cycle regulatory network may be lacking. However, one may expect that if the core design of a minimal and detailed models are similar, the general properties are the same as those of the more complete cell cycle models for both budding yeast^29^ and fission yeast^30^. Furthermore, the implementation time of the complex, yet powerful, framework provided by the SDS methodology is greatly simplified by utilizing the Systems Design Space Toolbox^31^.

In this work, we present the pioneer autonomously oscillating Clb/Cdk1 model for budding yeast, and explore the oscillatory behavior of 11 known and hypothetical network designs. We recover the known importance of PFLs and NFLs for oscillations. More specifically, we show that a PFL by Clb3/Cdk1 on *CLB3* synthesis (Clb3 PFL) improves the ability of our models to produce sustained Clb/Cdk1 oscillations, and that a PFL by Clb2/Cdk1 on *CLB2* synthesis (Clb2 PFL) takes over this key role when the model takes into account the inhibition of G1/S cyclins by Clb2/Cdk1. Furthermore, we show that two regulatory activations, i.e. Clb5 → Clb3 and Clb3 → Clb2, forming a transcription factor-mediated linear *CLB* cascade that we have recently discovered^32^ are more frequently dominant in phenotypes that yield sustained Clb/Cdk1 oscillations as compared to the feed-forward Clb5 → Clb2 regulation described earlier^33^. We thus hypothesize that functional (“healthy”) phenotypes require the capacity to oscillate autonomously—through Clb3-centered regulations—compared to dysfunctional (potentially “diseased”) cellular phenotypes—where these designs are altered and the potential for oscillatory behavior is reduced. We envision a scenario in which Clb5 and Clb2 are involved in the checkpoints, whereas Clb3-centered regulations that coordinate Clb5 and Clb2 drive autonomous cell cycle oscillations to maintain cell proliferation. This scenario thus reconciles checkpoint and oscillatory views of cell cycle regulation. In addition, we highlight that the transcriptional inhibition of G1/S cyclins and Sic1 by mitotic Clb/Cdk1 results in particularly strong NFLs for stabilizing oscillations. Finally, through perturbation of selected limit cycles, we identify crucial model parameters that exert the strongest control on the frequency of the Clb/Cdk1 oscillations.

Given the evolutionary conservation of the cell cycle network across eukaryotes, the mitotic cyclin/Cdk network can be used as a core building block of multi-scale models that integrate regulatory modules to address cellular physiology.

## Results

### Experimental rationale underlying the computational analyses

The cell cycle has a unique property as compared to other biochemical networks. Its drivers, i.e. the cyclin subunits that regulate the Cdk activity, have both specialized functions and partially overlapping functions, through different specificity of binding to the substrates that they recognize and—through their partner Cdk—phosphorylate^34^. Budding yeast cells lacking Clb5 (S phase cyclin) do not replicate at the proper time, but they do so progressively after activation of Clb2 (G2/M phase cyclin), which can partially substitute for the missing Clb5 activity; this indicates that a partial overlap in the cyclin function helps to drive DNA replication^35^. In these cells, the S phase is prolonged and the overall cell cycle timing is slightly delayed^36^. Conversely, cells lacking Clb2 (G2/M phase cyclin) exhibit defects in mitotic entry and delay in mitotic exit^37^; moreover, modified Clb2 degradation kinetics result in a compromised viability^38^. In these cells, Clb5 (S phase cyclin) and/or Clb3 (S/G2 cyclin) cannot substitute for the missing Clb2 activity, indicating the relevance of cyclin specificity for the events that trigger cell division.

Differently from Clb5 and Clb2, cells lacking Clb3 or cells where Clb3 degradation kinetics have been modulated are viable and complete cell division at the same timing as a wild-type cell^38^. In fact, Clb2 can replace Clb3 activity (Clb2 replaces Clb3 better than it does with Clb5; Clb2, and Clb3 have more structural and functional similarities than Clb2 and Clb5). Whereas Clb5 and Clb2 deletions affect dynamics of cell division timing as well as cell viability, Clb3 deletion does not affect cell cycle timing nor cell viability. Clb3 deletion is lethal only in the *clb2*Δ *clb3*Δ double mutant^37^, and in the *clb5*Δ *clb3*Δ *clb4*Δ^39^ and *clb2*Δ *clb3*Δ *clb4*Δ^37,40–42^ triple mutants, suggesting that Clb5 and Clb2, respectively, are required for spindle formation in the absence of Clb3 and Clb4.

Taking into account this experimental evidence, we envision a scenario where (i) Clb5 and Clb2 serve a function in checkpoint models (as currently incorporated in Tyson/Novák’s cell cycle models^20,21^), whereas (ii) Clb3 serves a function in autonomous oscillations required to sustain the cell’s viability. Specifically: (i) In Tyson/Novák’s cell cycle models, Clb5 and Clb2 represent the checkpoints that drive the cell cycle through the next cell cycle phase, should their concentration reach a definite threshold. In the cell, DNA damage/errors would activate the checkpoint affecting Clb5 levels, thus slowing/halting DNA replication dynamics, whereas troubles in cell division would activate the checkpoint affecting Clb2 levels, thus delaying/impairing cell division. In addition, the requirement of definite Clb5/Clb2 threshold concentrations may be seen as the result of a proper availability of nutrients which, if lacking, would not allow the thresholds to be reached, thus the cell cycle not to be completed. Conversely: (ii) Clb3 has never been considered in any existing (checkpoint) model of cell cycle regulation, possibly due to its not fully clear and not critical role in cell division. In our view, Clb3 serves a function in the cell’s autonomous oscillations. Clb3 is not involved in the checkpoints, as its deletion is lethal only in the *clb5*Δ *clb3*Δ *clb4*Δ^39^ and *clb2*Δ *clb3*Δ *clb4*Δ^40–42^ triple mutants, but not in the *clb5*Δ *clb3*Δ and *clb*2Δ *clb*3Δ^40^ double mutants. Furthermore, we have discovered, through a detailed computational and experimental investigation, the role of Clb3 in the coordination of the mitotic waves of cyclins, synchronized from the S through M phases in a linear cascade (Clb5 → Clb3 → Clb2) through the Fkh2 transcription factor^32^. In order to shed light on this hypothesis, in this study we have conducted a detailed computational analysis to investigate the occurrence of sustained oscillations in a minimal Clb/Cdk1 model and to identify recurring Clb-mediated principles of design, i.e. network motifs, underlying autonomous oscillations.

### The minimal cell cycle model and derivation of model designs *1A–3*

**Fig. 1:**
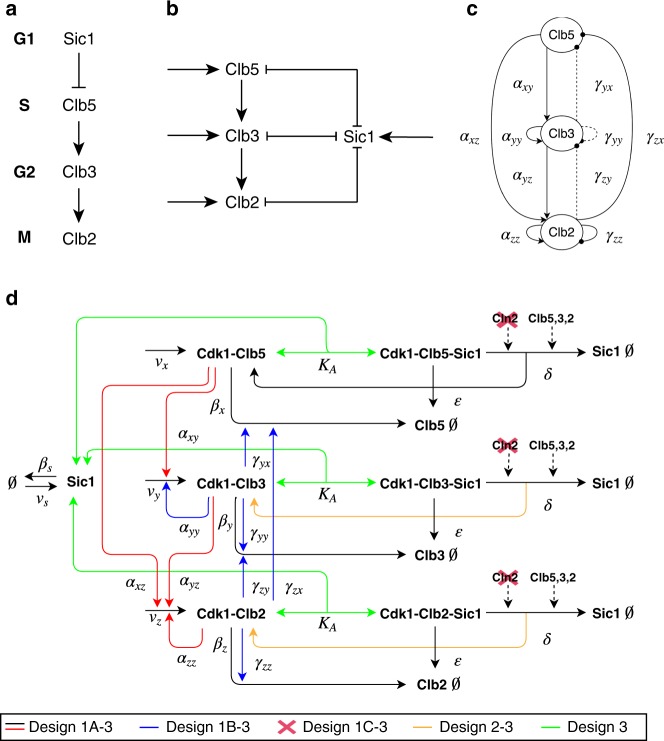
Schematic views of the minimal cell cycle model for budding yeast and full interaction diagram for designs *1A*, *1B*, *1C*, *2*, and *3*. **a** The molecular players driving phase-specific cell cycle events. **b** Linear cascade of the three Clb/Cdk1 complexes, and mutual inhibition between these and the Clb/Cdk1 inhibitor Sic1. **c** Interactions among the Clb/Cdk1 complexes. Solid lines indicate (7) proven interactions, whereas dotted lines indicate (3) hypothetical interactions (see Supplementary Table 2). **d** Full interaction diagram for designs *1A*, *1B*, *1C*, *2*, and *3* of the minimal cell cycle network. The scheme illustrates the core interactions in all model designs presented in this work, i.e. black and red arrows for the basal and activatory regulations, respectively, and highlights the progressive changes to the core structure introduced in designs *1A–3* (blue, red cross, orange, and green, respectively). Dotted arrows indicate the Cln(/Cdk1)- and Clb(/Cdk1)-mediated phosphorylation of Sic1 in Clb/Cdk1/Sic1 ternary complexes, resulting in its degradation. The complex formation between Clb/Cdk1 complexes and Sic1 is indicated with the *K*_*A*_ parameter, referring to the quasi-steady-state assumption introduced in *Design 3* (see Supplementary Information, Section 1.6.2), which should be taken to be the regular complex formation (*k*_+_ for formation, *k*_−_ for dissociation) for designs *1A–2*. Model derivations are reported in Supplementary Information, Section 1, and details of the reactions and their experimental evidence are reported in Supplementary Table 2.

Starting from our previously published minimal cell cycle model^10^ (*Design 1A*, Supplementary Information, Supplementary Fig. 1a), we built a number of mathematical models in terms of ODEs. The core design considers four species: (i) three representing the complexes that Cdk1 forms with the three pairs of B-type cyclins, Clb5,6, Clb3,4, and Clb1,2, and (ii) the inhibitor Sic1 that binds and inhibits all three Clb/Cdk1 complexes. Each of the four species is associated to cell cycle events during a specific phase of the cell cycle (Fig. 1a). The model describes (i) the progressive activation of the three Clb/Cdk1 complexes in a linear cascade, and (ii) the complex formation between Sic1 and the Clb/Cdk1 complexes, which are mutually inhibiting one another (Fig. 1b). The minimal model considers the complexity of all documented interactions among the Clb/Cdk1 complexes (Fig. 1c, solid lines, and Supplementary Table 2) in addition to two hypothetical interactions (Fig. 1c, dotted lines, and Supplementary Table 2). In addition, the model describes the degradation of the Clb/Cdk1/Sic1 complex, and the basal synthesis and degradation of each species, as visualized in the interaction diagram (Fig. 1d).

The existing model^10^ is rooted in experimental evidence, and the new models developed here were built considering: (i) recently unraveled experimental evidence, (ii) hypotheses generated on existing experimental evidence, and (iii) simplification of a number of reactions (Supplementary Table 2). This process resulted in five sequential model designs: *1A*, *1B*, *1C*, *2*, and *3*. The essential differences between the five designs are summarized in Fig. 1d and Supplementary Fig. 1. In Supplementary Information, Section 1, we document the step-by-step derivation of the five alternative network designs. *Design 3* presents a special case as it incorporates a quasi-steady-state approximation, which assumes that formation (*k*_+_) and/or dissociation (*k*_−_) of the Clb/Cdk1/Sic1 ternary complexes occur on a faster time-scale than the other processes considered in the model (see Supplementary Information, Section 1.6.2).

The main aim of this work is to identify limit cycles systematically across wide, distinct parameter regions and across multiple minimal model designs, in order to identify network motifs (presence of specific interactions and parameter values) that support the occurrence of oscillations. As a preliminary analysis, we investigated the ability of designs *1A*–*3* to generate: (i) transient cyclin/Cdk oscillations and (ii) sustained oscillations in the form of limit cycles. Finally, for each design, we performed a sensitivity analysis of how the model parameters influence the period of a single limit cycle. We observed that all five model designs are able to generate limit cycles, and that the basal synthesis and degradation parameters together with those responsible for the linear Clb cascade (Clb5 → Clb3 → Clb2) hold the strongest control over the length of the period of oscillation (see Supplementary Information, Section 2).

### Identification of conserved network motifs across oscillatory phenotypes

For a system of the size of model designs *1A*–*3* (7 species and over 20 parameters), there is generally no way to obtain all possible parameter sets that give rise to limit cycles, as an infinite number of these exists. However, a relevant question, interpretable biologically, is to address which network designs are able to generate oscillatory behavior in the form of limit cycles. The SDS methodology^25^ can be utilized to investigate such a capability of definite network designs. Specifically, the methodology allows to partition network designs into “phenotypes” that represent the dominance of certain reactions over others, thus neglecting all non-dominant ones. For a given set of parameters and concentrations, i.e. states, one activatory term and one inhibitory term in each differential equation are numerically larger than all others, i.e. dominant (see Methods section and Supplementary Information, Section 4). The dominance of a reaction implies boundary conditions, i.e. inequalities in the parameter and state spaces, which, if feasible, partition these spaces into areas referred to as “valid phenotypes”. Parameter sets that yield oscillations occur within a valid phenotype and therefore link the dominance of specific reactions to the occurrence of oscillations.

**Fig. 2:**
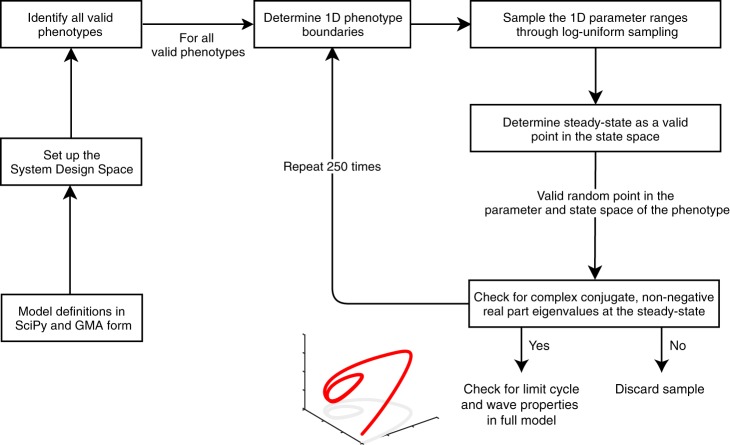
Computational pipeline implemented using the Systems Design Space Toolbox to identify oscillatory phenotypes in high-dimensional parameter spaces, for any model, using targeted parameter sampling. The procedure starts from a previously defined set of model equations in the format used by SciPy and the Systems Design Space Toolbox. All valid phenotypes with consistent boundary conditions in each model were identified. Each such valid phenotype was sampled log-uniformly 250 times. For each random sample, the parameter values were checked to lie within the parameter region defined by the phenotype and the steady-state was calculated. The potential for oscillation was identified by looking for the presence of two complex conjugate eigenvalues with non-negative real part. If this condition was met, the full model was analyzed for the presence of a limit cycle (see Methods section).

In order to analyze our models for the occurrence of limit cycle oscillations, we implemented a modeling pipeline based on the SDS methodology that incorporates a new approach to sample phenotypes for finding limit cycles, applicable to any network design. We implemented a parameter sampling procedure that makes use of the boundaries of a phenotype and employs log-uniform random sampling (see Fig. 2 and Methods section).

We set up model definitions in the Generalized Mass Action (GMA) form (see Supplementary Information, Section 4) for all the kinetic models representing five network designs considered in this work. In addition, we used the model presented by Fasani and Savageau^43^ as a test case to make sure that our implementation could recover their previously reported results. By using our pipeline (Fig. 2) we generated the SDS for each model variant, i.e. the set of all phenotypes, and retrieved the set of valid phenotypes (see Supplementary Information, Section 4). For our models, the valid phenotypes represented 0.044–0.34% of all theoretically possible phenotypes, demonstrating the importance of the SDS methodology to rapidly scan through the parameter space. This reduced the number of phenotypes for which the stability was investigated to a manageable number of several hundred to several thousand phenotypes (Table 1). After identifying the valid phenotypes, these were sampled to retrieve parameter sets yielding potential oscillations by using log-uniform random sampling. As a criterion for oscillations that emerge from a Hopf bifurcation, for each sampled parameter set we checked for the presence of a pair of complex conjugate eigenvalues with non-negative real part in the steady-state of a phenotype. If a particular combination of phenotype and parameter set satisfied this condition, and therefore showed potential for oscillations, the limit cycle behavior in the full kinetic model was tested using that specific parameter set.

**Table 1:** Number of total phenotypes, valid phenotypes, phenotypes with a potential for oscillations (presence of two non-zero eigenvalues), distinct phenotypes for which limit cycles were found, and limit cycles retrieved for model *Design 3* through *Design 9*.

Design #	Total phenotypes	Valid phenotypes	Potential for oscillations	Phenotypes with limit cycles	Limit cycles retrieved
**3**	995328	3355	664	7	8
**4**	1990656	6689	1158	66	204
**5**	1990656	6950	1523	16	23
**6**	1990656	6844	1508	20	31
**7**	1990656	6692	1239	19	24
**8**	1990656	6750	974	9	9
**9**	1990656	6977	677	13	21

We observed that in *Design 3*, with 250 samples for each valid phenotype, 664 phenotypes with a potential for oscillations were identified, and 8 limit cycles across 7 of these were found. Conversely, for *Design 1A* no positive complex conjugate eigenvalues were found, supporting the existing point of view that NFLs are required for sustained limit cycle oscillations^15,16^. Of note, this design is able to exhibit transient oscillations and sustained, limit cycle oscillations (see Supplementary Information, Supplementary Figs. 3 and 4). A possible reason for the failure of the SDS methodology to detect limit cycles may be the random sampling procedure, i.e. for this design, the sampling may just not have occurred in the area of the parameter space that allows for oscillations to occur. This may be especially likely if the region in the parameter space where such oscillation can occur is small. Furthermore, since the SDS methodology works with sub-models, which may have different stability properties than the full model, the full network’s parameter set that generates oscillations may lie in a phenotype whose stability does not meet the eigenvalue requirement that we used in order to check the full model. Designs *1B–2* exhibited limit cycles but with an incorrect order of peaking of the four model species so we did not further consider these. The results for the updated model *Design 3* can be directly compared to designs *1A* and *1B*, which are based on our published minimal model^10,32^; we conclude that the updated model outperforms its counterparts.

Each parameter set that yielded a limit cycle was stored, and the time-dependent oscillatory behavior was plotted (the parameter sets are available in the Supplementary Code Repository). For each phenotype for which a limit cycle was found, the terms in the differential equations that were dominant for that phenotype were identified. Inspection of these dominant processes allowed for counting the existence of specific parameters within these phenotypes (Table 2). In *Design 3*, *α*_*yy*_ (Clb3 PFL, responsible for Clb3/Cdk1 activation) and *α*_*yz*_ (Clb2/Cdk1 activation by Clb3/Cdk1) are the activatory parameters observed most frequently in phenotypes that yielded limit cycles. This finding suggests that the dominance of these two terms in the differential equations increases the ability to generate sustained oscillations, perhaps by enlarging the region within phenotypes of the design space where oscillations occur. The result supports the relevance of the linear *CLB* cascade through the Fkh2 transcription factor that we have recently discovered^32^—formed by the two regulatory activations Clb5 → Clb3 (*α*_*xy*_) and Clb3 → Clb2 (*α*_*yz*_)—over the Clb5 → Clb2 regulation (*α*_*xz*_) described earlier^33^. Altogether, these findings point to the relevance of Clb3 for generating sustained Clb/Cdk1 oscillations, through the dominance (i) of the Clb3 PFL and (ii) of the linear cascade (Clb5 → Clb3 → Clb2).

**Table 2:** Counts of the occurrence of parameters in dominant terms of phenotypes that yielded limit cycles for model *Design 3* through *Design 9*.

Design #	*α*_*xy*_	*α*_*yy*_	*α*_*xz*_	*α*_*yz*_	*α*_*zz*_	*γ*_*yx*_	*γ*_*zx*_	*γ*_*yy*_	*γ*_*zy*_	*γ*_*zz*_	*K*	*δ*	*ε*	*β*_*x*_	*β*_*y*_	*β*_*z*_	*β*_*s*_	*v*_*y*_	*v*_*z*_
3	0	*6	0	*6	1	1	^#^5	1	4	0	–	^‡^7	3	1	0	4	0	1	0
4	*41	23	1	17	*48	12	20	12	18	4	^†^54	^‡^46	^‡^63	14	11	5	0	2	0
5	3	*12	2	*10	4	0	^#^11	0	^#^13	0	8	^‡^14	^‡^12	5	1	4	0	1	0
6	3	*17	0	*13	7	3	^#^13	2	^#^12	1	10	^‡^16	^‡^15	3	2	4	2	0	0
7	6	*12	2	9	7	3	^#^14	3	^#^12	0	^†^16	^‡^18	^‡^13	2	1	6	0	1	1
8	2	*6	3	3	2	2	^#^6	0	^#^6	2	2	^‡^9	^‡^6	1	0	3	0	1	1
9	2	*11	1	*9	3	2	^#^11	1	^#^10	1	1	^‡^12	7	0	1	5	0	0	0

The counts listed are subsets of the numbers in the column “Phenotypes with limit cycles” in Table 1. Parameters that are present in all phenotypes, due to the model design, are not shown (*v*_*s*_, *v*_*x*_, *K*_*A*_). The generic *K* parameter modulates the strength of the unique new inhibitions in designs *4–9*. For example, in *Design 4*, *K* represents the parameter *K*_*zx*_, which refers to the transcriptional inhibition of Clb2/Cdk1 (*z*) on Clb5/Cdk1 (*x*). Parameters that are part of terms that were dominant in more than 60% of all phenotypes that yielded limit cycles per design (Table 1) are marked with symbols in groups: activatory (*) and inhibitory (^#^) interactions among the Clb/Cdk1 complexes, new inhibitory interactions in designs *4–9* (^†^), and basal synthesis and degradation reactions (^‡^).

With respect to the inhibitory regulations, we observed that the Clb3 NFL (*γ*_*yy*_) and the Clb2 NFL (*γ*_*zz*_) terms are rarely dominant in phenotypes exhibiting limit cycles (Table 2). Conversely, the parameters referring to the APC-mediated inhibition of Clb5 and Clb3 by Clb2 (*γ*_*zx*_ and *γ*_*zy*_, respectively), and to the degradation rates of Sic1 and Clbs from the Clb/Cdk1/Sic1 ternary complex (δ and ε, respectively) were observed more frequently with respect to the generation of sustained cyclin/Cdk1 oscillations.

### Alternative network designs of the minimal cell cycle model

**Fig. 3:**
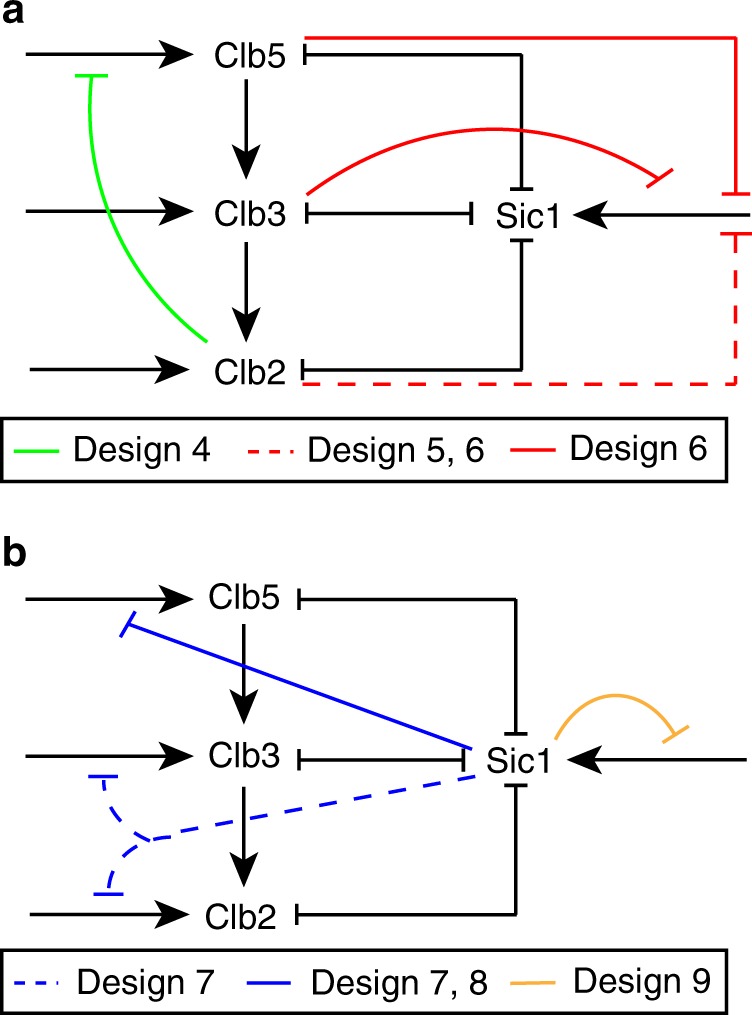
Schematic view of known and hypothetical inhibitory regulations added to *Design 3* of the minimal cell cycle model. **a** *Design 4*, *Design 5*, and *Design 6* describe known inhibitory regulations mediated by Clb/Cdk1 activities. **b** *Design 7*, *Design 8*, and *Design 9* describe hypothetical inhibitory regulations mediated by Sic1. Colored lines indicate new regulations, with each color identifying a particular network design. Dashed lines indicate regulations occurring in two different designs. In the latter, the two designs related to each such interaction are shown with the same color. Black lines indicate the activatory and inhibitory regulations occurring in the minimal cell cycle model. See text and Supplementary Information, Section 3 for details about the specific molecular mechanisms.

To further analyze the oscillatory behavior of our minimal cell cycle model, we extended *Design 3* to test six new network designs that include further known or hypothetical inhibitory regulations. By doing this, we aim to understand whether and how these regulations might enable the cyclin/Cdk network to generate limit cycles. The new designs are based on *Design 3*, and are referred to as *Design 4* through *Design 9* (Fig. 3). Each new design reflects either a single or several related inhibitory regulations. *Design 4*, *Design 5*, and *Design 6* describe known inhibitory regulations mediated by Clb/Cdk1 activities (Fig. 3a), whereas *Design 7*, *Design 8*, and *Design 9* describe hypothetical inhibitory regulations mediated by Sic1 (Fig. 3b). In the following, each design is described succinctly. Both detailed molecular mechanisms and equations supporting the designs are reported in Supplementary Information, Section 3.

*Design 4* essentially adds a long NFL, which is known to have a positive effect on the oscillatory behavior. It incorporates the inhibition of Clb5/Cdk1 by Clb2/Cdk1 through the MBF transcription factor, formed by Mbp1 and Swi6. Clb2 has been shown to interact physically with Swi4, and to repress transcription of the G1 cyclins^44^. This inhibition translates to an effective inhibition of the Clb5/Cdk1 activity, due to the lack of the PFL between the G1 phase Cln2/Cdk1 complex and SBF/MBF^45^ as well as due to the lifted inhibition of Sic1 by Cln1,2/Cdk1 (ref. ^46^).

The designs *5* through *9* essentially add PFLs (or double NFLs) which could potentially provide stable states and increase robustness of the oscillatory behavior. *Design 5* and *Design 6* incorporate the inhibition of Clb/Cdk1 on *SIC1* transcription through the *SWI5* transcription factor. Specifically, *Design 5* describes the inhibition of *SIC1* transcription mediated by the Clb2/Cdk1 activity^47^, reflecting the likely scenario where the most abundant Cdk1 activity is due to Clb2/Cdk1. *Design 6* describes the same mechanism mediated by the three Clb/Cdk1 complexes: Clb2/Cdk1, Clb3/Cdk1, and Clb5/Cdk1.

*Design 7* and *Design 8* incorporate the hypothetical inhibition of Sic1 on mitotic *CLB* transcription to rationalize a recent observation that Sic1 oscillations rescue viability of cells with low levels of mitotic Clb cyclins^48^. Specifically, *Design 7* describes the inhibition of Clb2 and Clb3 synthesis—which we have recently shown to be regulated by a similar transcriptional mechanism^32^—by Sic1. *Design 8* describes the hypothetical inhibition of Clb5, Clb3, and Clb2 syntheses by Sic1. Finally, *Design 9* incorporates the hypothetical inhibition of Sic1 synthesis by a Sic1-mediated NFL through *SWI5*. This design adds a short NFL which should not contribute the oscillation if there is no time-delay.

### Ability of known and hypothetical network designs to generate sustained Clb/Cdk1 oscillations

To retrieve limit cycles for the new network designs *4*–*9*, we again employed the pipeline described in Fig. 2. Each design yielded several hundred phenotypes that corresponded to parameter space regions with two non-negative complex conjugate eigenvalues, and all designs yielded a set of limit cycles across multiple phenotypes (Table 1). Specifically, all designs yielded a higher number of limit cycles and more distinct phenotypes with limit cycles than *Design 3* did. This points to a stronger tendency for these designs to oscillate due to their new inhibitory regulations. Interestingly, *Design 4* outperformed all other designs in terms of the number of limit cycles retrieved, followed by *Design 6*, *Design 7*, and *Design 5*. The computational results obtained for *Design 4* and *Design 5* indicate a role in generating and stabilizing sustained oscillations for the two inhibitory regulations experimentally observed: (i) Clb2/Cdk1 on Clb5/Cdk1, indirectly, through Swi4 (ref. ^44^), and (ii) Clb2/Cdk1 on Sic1, directly, through Swi5 (ref. ^47^).

Among the hypothetical designs, *Design 6* is an extension of *Design 5*, which is experimentally supported. *Design 6* performs better, suggesting that it should be beneficial for a cell to have all Clb/Cdk1 activities inhibiting *SIC1* transcription. This finding is a testable prediction. Among the hypothetical designs that are not yet supported by experimental evidence, *Design 7*, which describes the inhibition of *CLB2* and *CLB3* syntheses by Sic1, exhibits the highest number of parameter sets in which limit cycles occur with 24 sampled limit cycles across 19 distinct phenotypes. This finding suggests the possible relevance of transcriptional inhibition mediated by Sic1 to guarantee a self-sustaining cell cycle.

As we exemplified for *Design 3*, we quantified the occurrence of parameters in the dominant terms (processes) for designs *4–9*, identifying phenotypes that (i) are valid, (ii) have a potential for oscillations, and (iii) yield autonomous limit cycles (Table 1, Supplementary Code Repository). As we showed for *Design 3*, among the phenotypes that yield limit cycles, in designs *5–9* *α*_*yy*_ (Clb3 PFL) is the parameter observed most frequently among the activatory regulations, followed by *α*_*yz*_ (Clb2/Cdk1 activation by Clb3/Cdk1) (Table 2). Intriguingly, when adding the inhibition of Clb5/Cdk1 by Clb2/Cdk1 in *Design 4*, the Clb2 PFL (*α*_*zz*_) becomes the most dominant design. This likely reflects the crucial role of Clb2/Cdk1 in the modulation of *CLB5* synthesis, thus reinforcing the importance of PFLs in the occurrence of sustained oscillations.

Furthermore, in all designs except for *Design 3* and *Design 8*, both steps in the linear Clb cascade^32^, *α*_*xy*_ (Clb5 → Clb3) and *α*_*yz*_ (Clb3 → Clb2), are more frequent than *α*_*xz*_ (Clb5 → Clb2), and in designs *5–9*, *α*_*yz*_ is equally or more frequent than *α*_*zz*_ (Clb2 PFL). In fact, in all designs except for *Design 4*, the Clb3 → Clb2 activation is the second dominant activatory regulation. Furthermore, once again, *γ*_*zx*_ (the APC-mediated inhibition of Clb5 by Clb2/Cdk1) and *γ*_*zy*_ (the APC-mediated inhibition of Clb3 by Clb2/Cdk1) are the parameters observed most frequently among the inhibitory regulations. The degradation rate of Sic1 from the Clb/Cdk1/Sic1 ternary complex (δ) was present in dominant terms of the majority of limit cycles for designs *3–9*. To a lesser extent, the same holds true for the degradation rate of the Clbs from those complexes (*ε*). Finally, the two new interactions in *Design 4* and *Design* 7 (inhibition of Clb5 synthesis by Clb2 and inhibition of Clb2 and Clb3 synthesis by Sic1 respectively) stand out in their contribution to the high number of limit cycles observed for these designs. For these two model designs the new inhibitory interactions were dominant in nearly all identified limit cycles.

We additionally calculated the Pearson correlation coefficients between all parameter combinations across Designs *3–9* (Supplementary Information, Section 6, Supplementary Fig. 19 and Supplementary Table 3). In line with the observations above, the parameters related to the Clb3 PFL and the APC-mediated inhibition of Clb3 by Clb2/Cdk1 are highly positively correlated in six out of the seven model designs (see Supplementary Table 3). This is in line with the observation that both interactions occur as often dominant activatory and inhibitory terms, respectively (see Table 2). Intriguingly, even in *Design 4*, for which we observed a shift from the Clb3 PFL to the Clb2 PFL as the most often dominant activatory term in the limit cycles, this correlation remains high. This indicates that, even though the Clb3 PFL is more rarely dominant in this design, its strength still needs to be balanced by a Clb2/Cdk1-mediated inhibition.

Altogether, our findings highlight that the Clb3 and Clb2 PFLs, together with the linear cascade (Clb5 → Clb3 → Clb2) and the APC-mediated inhibitions driven by Clb2 are principles of design that may underlie a self-sustaining cell cycle network in budding yeast.

### Limit cycles belong to distinct phenotypic regions spread across the parameter space and showcase a range of oscillation properties

Our analysis for designs *1–9* has retrieved hundreds of parameter sets that generate limit cycles (Table 1). A critical question to be addressed is whether these limit cycles belong to different, distinct regions in the parameter space. Part of this question is addressed by the fact that, for each design, we have identified limit cycles across multiple phenotypes; this implies that different interactions and regulations are dominant across (some of) the limit cycles, and that they belong to distinct parameter space regions. However, design space phenotypes may overlap in the parameter space. A key concept here is that of the *robustness region*, i.e. the parameter space region around a limit cycle point within which parameters can be smoothly altered without interrupting the limit cycle behavior^49^. Generally, it is challenging to obtain a good approximation to a robustness region around a single point let alone compare multiple such regions for overlap.

To explore whether the identified limit cycles belong to non-overlapping robustness regions, we analyzed the spread of the parameter values through boxplots and by Principal Component Analysis (PCA) projection. For all designs, most parameter values cover multiple orders of magnitude (see Supplementary Information, Section 7 and Supplementary Fig. 20). Interestingly, some parameters are consistently narrow in range across all designs: *K*_*A*_, *v*_*s*_, and *v*_*x*_ (referring to the complex formation between Clb/Cdk1 complexes and Sic1, to the basal synthesis of Sic1 and to the basal synthesis of Clb5, respectively), indicating that they need to be tightly controlled in order to generate sustained oscillations. Conversely, *α*_*yy*_ (Clb3 PFL) is narrow in range in all designs except for *Design 4*, and vice versa for *α*_*zz*_ (Clb2 PFL). This finding supports the observations of the flipped dominance of these parameters across the designs shown in Table 2. Similarly, in each design, either *δ* or *ε* show a narrow range. Interestingly, *α*_*yz*_ (Clb3 → Clb2), second step in the linear cascade (Clb5 → Clb3 → Clb2), shows a higher median value than the first step in all designs except for *Design 4*, in agreement with its previously observed dominance.

**Fig. 4:**
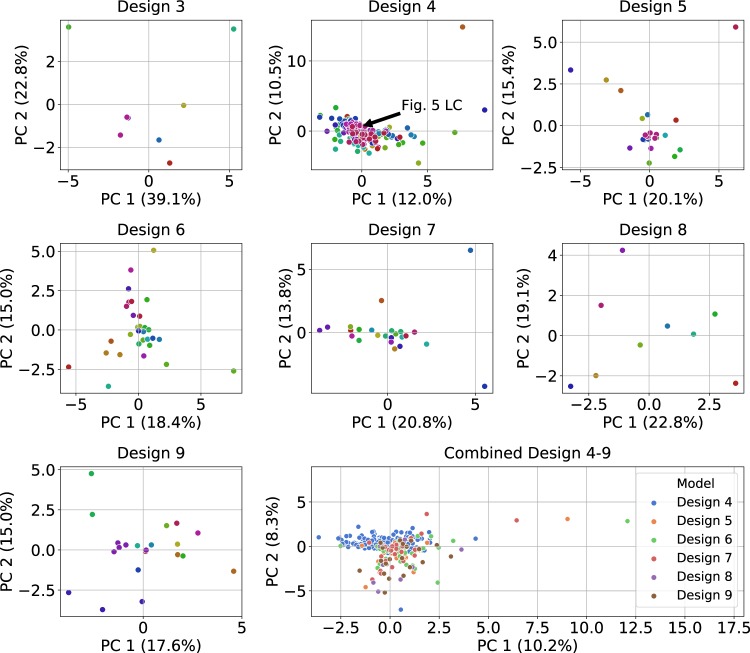
Projection of limit cycle parameter sets onto the first two principal component axes, for designs *3–9* separated and for designs *4–9* combined (bottom-right). Each dot represents a parameter set yielding a limit cycle. Parameter values were normalized to have a mean of zero and a standard deviation of 1 prior to principal component calculations, in order to deal with parameters spanning different orders of magnitudes. For the single panels, the colors indicate the unique phenotype each parameter sets belongs to. For the combined panel, the colors indicate the model design. For the purposes of this analysis, the unique inhibitory parameters in designs *4–9* were treated as the same parameter. The percentage of variance in the data explained by each principal component is listed on the axes. Values along the axes should be compared to the [0, 1] unit interval since the principal components have a length of 1 and are linear combinations of the normalized parameters.

Subsequently, we performed PCA (see Methods section) on the limit cycle parameter sets for designs *3–9* to visualize how the parameter values are spread throughout the 22-dimensional parameter space (Fig. 4). The limit cycles are spread across the two main principal components in the parameter space. For *Design 4*, many points appear to clump together; however, the scale on the axes is larger for this design than for the others, and many more limit cycles are found for this design which increase the overlap. This result is strengthened by the fact that for all designs the first two principal components never explain more than 62% of the variance in the data, indicating that there is also significant variance in the data along other orthogonal directions in the parameter space. For all designs in Fig. 4, limit cycles are separated both within and between phenotypes, indicating that, even within a single phenotype, limit cycles are found that are spread across different areas of the parameter space. The results illustrate the complex distribution of the parameter sets that support phenotypes that exhibit limit cycle oscillations. A similar result was obtained by aggregating the limit cycles across designs *4–9* (bottom-right panel in Fig. 4). The aggregated results for designs *4–9* highlight that there are different areas of the parameter space that produce oscillations for different network designs.

Our analysis does not prove that the limit cycles do not belong to one or a few continuous robustness regions (i.e. could potentially be found by continuation techniques), but indicates that the combined robustness region would have to cover multiple orders of magnitude in most dimensions. Given that the vast majority of our parameter samples did not lead to limit cycles, such a vast robustness region may seem unlikely. Altogether, Fig. 4 and Supplementary Fig. 20 suggest that either some of our limit cycles belong to different robustness regions or the robustness region must take a highly complex and large N-dimensional shape.

We further quantified the differences in oscillation properties between the limit cycles by looking at the period of the oscillation and at the minimal percentage of the oscillation amplitude with respect to the maximal concentration across species (see Supplementary Information, Section 8). In Supplementary Figs. 21 and 22 boxplots of the period and amplitude (in terms of the minimum/maximum ratio), respectively, for designs *3–9* are shown. It can be observed that the limit cycles display a wide range in both properties; the wide range of parameter values covered between the limit cycles translates to differential oscillation properties.

**Fig. 5:**
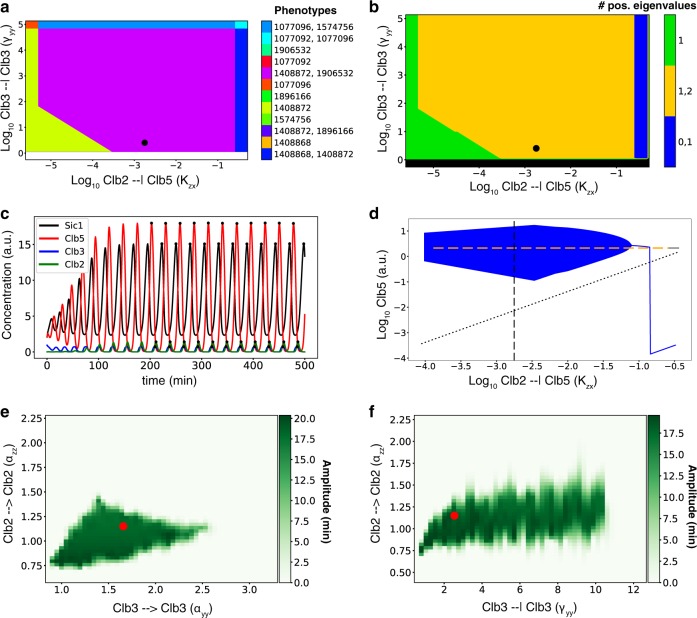
Robustness of a limit cycle for phenotype number 1906532 in *Design 4*. Black dot in **a** and **b**, time course in **c**, dotted line in **d**, and red dot in **e** and **f** represent the same limit cycle and parameter set. **a** 2D slice *K*_*zx*_–*γ*_*yy*_ of the design space visualized in regions corresponding to different phenotypes. White color represents absence of phenotypes, whereas other colors indicate specific phenotypes, or combinations of overlapping phenotypes. Some regions relate to multiple phenotypes, indicating that these phenotypes overlap in this 2D projection of the parameter space. **b** The same 2D phase plane as in **a**, visualizing the number of positive eigenvalues in the steady-state of the phenotype. Black color represents absence of phenotypes, whereas the orange area supports oscillatory behavior. **c** Sustained oscillation time course of the limit cycle. **d** 1D bifurcation diagram plotting the oscillation amplitude (minimum and maximum of the oscillation) in the full model of the limit cycle for Clb5 (x on the *y*-axis and in the equations) while varying the *K*_*zx*_ parameter. Blue line represents the range of *K*_*zx*_ values yielding stable steady-state behavior, i.e. no oscillations, whereas the blue area represents parameters values yielding oscillations, while keeping fixed all other parameter values. Yellow dashed line indicates an unstable steady-state with two non-negative eigenvalues in the phenotype. Gray dotted line indicates an unstable steady-state in the phenotype that has one non-negative eigenvalue. Solid gray line indicates a stable steady-state; as there is more than one line, multi-stability occurs. **e** 2D robustness region heatmap of the amplitude of oscillations (0 and white in case of a steady-state) within a 2D slice of the parameter space. 1,000 random log-uniform samples of the parameters *α*_*yy*_ and *α*_*zz*_ were retrieved while keeping all other model parameters fixed. For each sample, the amplitude of oscillations in Clb5 is represented by the scale of green color. The radial basis interpolation algorithm (RBF) has been applied to infer the color for points in the plot that were not explicitly sampled. **f** Similar 2D robustness region heatmap as in **e** for *α*_*zz*_ and *γ*_*yy*_.

As an illustrative example of the results that we obtained, we show a robustness analysis of a limit cycle of phenotype number 1906532 for *Design 4* (Fig. 5). We highlighted this limit cycle in the PCA plot for *Design 4* shown in Fig. 4; this limit cycle sits relatively close to most limit cycles found for *Design 4*. In Fig. 5a, b, a 2D slice of the parameter space across the *K*_*zx*_–*γ*_*yy*_ plane (two dominant inhibitory regulations in this phenotype) is shown, where the phenotype number 1906532 (purple) borders with several other phenotypes (Fig. 5a) and the stability in terms of the number of non-negative eigenvalues within these phenotypes may be observed (Fig. 5b). The black dot in Fig. 5a, b indicates a limit cycle that we identified—belonging to phenotype 1906532—which sits in an area where oscillations can be expected based on the eigenvalues. Due to the presence of two non-negative eigenvalues, the orange area in Fig. 5b is suggested to support oscillatory behavior. Consistent with the stability indications, limit cycles were not retrieved for most of the phenotypes in Fig. 5a based on our sampling (only phenotype 1574756 and 1906532) which fall in the orange area. The overlapping regions from Fig. 5a where multiple phenotypes are simultaneously valid in the same region of the design space are shown to support multi-stability in Fig. 5b. We observed two areas where multi-stability may occur, indicated by the presence of 0 and 1 positive eigenvalues or, alternatively, 1 and 2 positive eigenvalues. In Fig. 5c, properties of the limit cycle dynamics can be observed: (i) a period of about 27 min, corresponding to a frequency of 0.037 min^−1^; (ii) a similar (within 10-fold) order of magnitude of the amplitudes of the concentration of the four species considered (Sic1, Clb5, Clb3, and Clb2), consistent with previous observations^50,51^; (iii) the correct temporal order of peaks of the four species considered: Sic1 in the G1 phase, Clb5 in the S phase, Clb3 in the G2 phase, and Clb2 in the M phase^10^; and (iv) the amplitude of the oscillations covering most of the concentration range of the four species, i.e. their concentration sharply decreases and, in the case of total Clb3 and Clb2 becomes equal to zero when starting a new, successive cell cycle, as shown experimentally.

In Fig. 5d, a 1D bifurcation diagram is shown for the parameter *K*_*zx*_, which refers to the transcriptional inhibition of Clb5/Cdk1 (*x*) by Clb2/Cdk1 (*z*) that is unique to *Design 4*. We observed that there is a range of *K*_*zx*_ values (in blue color) where sustained oscillations occur in the full model. The amplitude changes with the bifurcation parameter (indicated by the vertical size of the violet area). We observe a general agreement between the region in Fig. 5b, where the phenotypes support two positive eigenvalues, and the “robustness region”^49^ of the limit cycle in Fig. 5d; however, the robustness region of the limit cycle is smaller than predicted by the eigenvalues of the phenotypic subsystem (yellow line). In Fig. 5e, a 2D parameter scan for the Clb3 PFL (*α*_*yy*_) and the Clb2 PFL (*α*_*zz*_) is shown in the form of what we consider as a “robustness region”. Figure 5f shows a similar robustness region for the Clb2 PFL (*α*_*zz*_) and the Clb3 NFL (*γ*_*yy*_). These regions visualize how far the chosen parameters may be changed around the limit cycle such that the qualitative behavior does not change, and are represented as heatmaps, where the color scale indicates the amplitude of oscillations. In this way, it is possible to investigate how mutations or environmental perturbations that lead to changes in model parameters can change or break the oscillatory behavior. This analysis highlights the complexity of combinations of parameters for which sustained oscillations may occur, and that, around the limit cycle identified by our pipeline (shown as a red dot), there is a region of the parameter space where oscillations are robust to change in these two parameters (albeit with a varying amplitude). This analysis highlights that this limit cycle is particularly sensitive to changes in the Clb2 PFL.

The analysis above can be used to identify phenotypes that are functional or dysfunctional based on specific features, e.g. in the tendency of a network design to oscillate. For instance, we can identify functional, healthy phenotypes that have a strong tendency to oscillate (Fig. 5a, phenotype in violet color), as compared to dysfunctional phenotypes, which exhibit no oscillations (Fig. 5a, b, all phenotypes except for 1906532 and 1574756) or a reduced tendency to oscillate in only a small area of the parameter space, reflecting a reduced robustness of the limit cycle.

## Discussion

In budding yeast, cell cycle networks are modeled through checkpoint mechanisms, where the starting point of oscillations is reset upon reaching specific concentration thresholds of certain network components^20,21^. Although oscillations in the cell cycle network should not be surprising, given the existence of network motifs (PFLs, NFLs, and their combination) that produce oscillations^52^, it is challenging to investigate whether minimal cell cycle models could generate such autonomous (limit cycle) oscillations. This challenge has been met for some organisms^8,15^, but not for budding yeast.

In this work we showed, to our knowledge for the first time, that some designs of the cell cycle network of budding yeast are suited to support truly autonomous limit cycle oscillations, independent of checkpoint mechanisms, for a wide range of parameter sets. Specifically, we studied whether known or hypothetical designs may modulate the tendency to generate or stabilize sustained oscillations. We considered a minimal model of the network governing the activation of the mitotic cyclin/Cdk1 (Clb/Cdk1) complexes in budding yeast^10^, and analyzed 11 alternative network designs for their ability to yield limit cycles (Supplementary Information, Sections 1 and 3). The model under investigation describes the sequence of events from the G1 through the M phases of the cell cycle and back to G1 again, assuming that to each phase is assigned one major functional component: Sic1, stoichiometric inhibitor of the Clb/Cdk1 complexes^53^, to the G1 phase; Clb5/Cdk1, which promotes DNA replication dynamics^54^, to the S phase; Clb3/Cdk1, which is involved in the spindle assembly^55^, to the G2 phase; and Clb2/Cdk1, which promotes spindle formation and cell division^56,57^, to the M phase. In seven of these designs, from *Design 3* through *Design 9*, a quasi-steady-state approximation was introduced, which assumes an equilibrium between the Clb/Cdk1/Sic1 ternary complex and its free components, Clb/Cdk1 and Sic1. This assumption is new in cell cycle models.

Our modeling effort, unlike existing cell cycle models that have been investigated in terms of their potential to show oscillations^8,20,21^, supports the implicit hypothesis that there is a functional reason for the cell cycle itself being an autonomous limit cycle oscillator. Aside from the uniqueness of our work in terms of the methodology that we have employed, we make a case for autonomous oscillations. The two opposing hypotheses of checkpoint models (i.e. the cell cycle system, by itself, should not favor oscillations, as these would disappear upon activation of checkpoints due to a cell’s response to cellular damage, or to a not favorable response to environmental cues) versus autonomous oscillations (i.e. the cell cycle system, by itself, should tend to exhibit self-sustained oscillations, independently from stimuli from the environment) are rather hard to prove or disprove. This is because oscillations do exist in living cells that may be due to cell cycle regulation alone, or to its interplay with the rest of the cell such as external factors, metabolic cues, etc. The fact that autonomously oscillating cell cycle models exist for mammalian cells^8^, does not provide any strong support for either of the two hypotheses, because cell cycle models, overall, need to oscillate, and may have been designed by evolution to do so. In the autonomous limit cycle models, limit cycle oscillations are identified by the presence of two complex conjugate eigenvalues with positive real part. In the checkpoint models, this property may or may not be present because—at specific points in the model dynamics—either the concentrations^20,21^ or both the concentration and the network wiring can be changed depending on the model under examination. The resetting of the position in the state space can force the model dynamics into a repetitive pattern that would not occur (in the same way) without the checkpoint(s).

The cell cycle models for budding yeast are currently incomplete: they require the help of the modeler or the computer program to break and then restart the model at the end/beginning of every cycle. Our model is a limit cycle model, which cycles by itself without any periodic resetting. We developed a new methodology, based on work by Savageau and colleagues, to the point that we could scan the parameter space for many possible limit cycle models, by adding a search for complex conjugate eigenvalues with positive real part expected around Hopf bifurcations. This produced a type of model that then enabled us to determine which parameters control the occurrence and period of the cell cycling of yeast.

Our cell cycle model structure was not designed to yield oscillations in general, but we found that it can yield oscillations. Specifically, our work highlights that the underlying mechanisms of these oscillations in our models are the Clb3-centered regulations—never considered in any available checkpoint model of cell cycle regulation in budding yeast—which we have shown to exist in budding yeast cells^32^. The prediction that Clb3-centered regulations are the highest represented network motifs that lead to self-sustained, autonomous oscillations, provides a more subtle proof, and evidence reconciling the checkpoint and autonomous oscillation views. Specifically, our results suggest that autonomous oscillations driven by Clb3/Cdk1 may occur when this complex is coupled and coordinated to the other S and M phase kinase complexes, Clb5/Cdk1 and Clb2/Cdk1. Whereas Clb5(/Cdk1) and Clb2(/Cdk1) have been described to be involved in the checkpoint mechanisms (Tyson and Novák’s types of models), we propose that Clb3(/Cdk1) drives autonomous cell cycle oscillations to maintain cell proliferation. Clb3 being tightly coordinated together with Clb5 and Clb2, we envision that Clb3-mediated oscillations are maintained unless an activation of checkpoints terminates the autonomous oscillations.

Our findings do not rule out checkpoint mechanisms, but add an aspect that does not appear in pure checkpoint mechanisms, i.e. designs yielding autonomous oscillations. If we assume that only checkpoint mechanisms exist, then there would be no reason to expect such designs to occur in reality. However, the fact that they do occur supports the hypothesis that generating oscillations may provide an evolutionary advantage. Therefore, we hypothesize that “health” requires the capacity to oscillate autonomously—investigated in detail in this study—and the capacity to interrupt the oscillation due to checkpoint activation and/or unfavorable environmental cues—not investigated in this study and subject of a future modeling project.

It has been demonstrated^12^ that including stochastic effects in checkpoint-based cell cycle models, using Langevin-type equations, can lead to qualitative changes in model dynamics and noise-induced oscillations. In this way, the dichotomy between checkpoint models and limit cycle oscillators is partially overcome by stochasticity, since checkpoints may be overcome through random fluctuations. Noise-induced oscillations also imply that inclusion of stochastic effects (in cell cycle models) may reshape and enlarge the regions in the parameter space that support oscillatory behavior. Experimental work regarding the role of noise in cell cycle regulation has been shown. Baumann and colleagues^58^ showed that stochastic telophase arrest of budding yeast mutants cannot be captured by a deterministic model, but can partially be captured by a stochastic model. Similarly, by using a stochastic model of the G1/S transition, we showed that entrance into the S phase is dependent on tight control of *SIC1* mRNA transcription and degradation^59^. Moreover, Peccoud, Tyson and colleagues measured experimentally the size of fluctuations in mRNA levels of 16 proteins that are important in the cell cycle by using time-lapse fluorescence microscopy^60^ and smRNA FISH^61,62^ and improved their cell cycle model to match the observations. Further development to incorporate the role of noise in cell cycle models in relation to sustained oscillations calls for software and methods supporting bifurcation analysis of stochastic differential equation models, also within the context of the SDS methodology that we have used in this study or extensions thereof.

To study the dynamic effects of the designs over a wide range of parameter values, we applied the SDS methodology to analyze the phenotypes that the 11 network designs partitioned the parameters and state space into. These phenotypes can be associated to areas of the parameter space in which sustained oscillations in the form of limit cycles can occur. The ability to enumerate the phenotypic repertoire of each of the designs, and to explore the behavior of each phenotype in a model, allows for desired properties to be readily identified^27^. This, in turn, helps to reduce the computational effort by focusing the search of limit cycles on specific regions in the parameter space. The SDS methodology is therefore useful when studying natural systems and when engineering synthetic networks intended to be endowed with particular characteristics^63^.

After applying our pipeline to identify oscillatory phenotypes (Fig. 2), we retrieved limit cycles for the network designs *3–9* but not for the designs *1A–2*. The latter lack the quasi-steady-state approximation, which is instead implemented in the former. The lack of observed oscillations in *Design 1A* provides an interesting case that is in line with the prevalent view that NFLs are required for sustained oscillations^15,16^. Remarkably, the Clb3 PFL is recurrent in all network designs that yielded sustained oscillations except for *Design 4*, where the Clb2 PFL takes over. Strikingly, PFLs have been shown to promote oscillations and switch-like responses that allow unidirectionality of cell cycle progression, by enhancing amplitude and robustness of cyclin/Cdk oscillations^5,13,14^. Our finding that PFLs are important for obtaining sustained oscillations in the cell cycle is in agreement with these previous studies. In recent work, Novák and colleagues^64^ highlighted the importance of the PFL between SBF and Cln1,2 for the cell size checkpoint in G1 phase by using checkpoint models. Similarly, they highlighted the importance of two antagonistic PFLs of Cdk1:CycB and PP2A:B55 for interphase–M phase transitions in the mammalian cell cycle, by using a non-checkpoint model that exhibits bistability and hysteresis^65^. Contrarily to these two studies, our work focused on autonomous oscillations rather than on multiple different steady-state attractors. A new insight from our work is that it appears that our models do not require both the Clb3 and Clb2 PFLs but, depending on the absence or presence of the inhibition of Clb5/Cdk1 by Clb2/Cdk1 (designs *3*, *5–9* vs. *Design 4*), one PFL has a more stabilizing effect on the oscillations than the other. The general result that we retrieve concerning the NFLs and PFLs is in agreement with the literature. However, the specific (combinations of) PFLs and NFLs that we found in oscillating phenotypes and parameter sets (see Table 2) were not predictable a priori.

Furthermore, the regulatory activation Clb3 → Clb2 (*α*_*yz*_), which forms the linear *CLB* cascade^32^ together with Clb5 → Clb3 (*α*_*xy*_), is observed more frequently than Clb5 → Clb2 (*α*_*xz*_). Therefore, our analyses point to the possible relevance of Clb3-centered regulations for the generation of sustained Clb/Cdk1 oscillations in budding yeast. Importantly, our results for *Design 4* and *Design 5* support the experimental evidence that the inhibitory regulations, Clb2/Cdk1 on Clb5 synthesis in *Design 4* and Clb2/Cdk1 on the synthesis of the Clb/Cdk1 inhibitor Sic1 in *Design 5*, play a crucial role in cell cycle regulation.

Among the hypothetical network designs, *Design 7* is of particular interest because it rationalizes a recent experimental observation for which the molecular mechanisms remain at the moment obscure. This design describes the inhibition of *CLB2* and *CLB3* syntheses by Sic1, as an attempt to describe the experimental evidence that Sic1 oscillations rescue viability of cells with low levels of mitotic Clb cyclins^48^. Our findings indicate that including this inhibitory regulation resulted in a higher number of limit cycles as compared to other hypothetical network designs. This result suggests that Sic1 inhibition on the synthesis of *CLB2* and *CLB3* may be relevant to guarantee a self-sustained cell cycle. We speculate that this inhibitory regulation might occur through a physical interaction of Sic1 on transcription factors that drive synthesis of these mitotic cyclins, such as Fkh2, which we have described to be the regulator driving both *CLB2* and *CLB3* transcription^32^. The mammalian counterpart of Sic1 (ref. ^66^), the cyclin/Cdk inhibitor p27^Kip1^, has indeed been shown to behave as a transcriptional repressor, by binding to and inhibiting a number of gene promoters through E2F4/p130 complexes^67^. The specific role of p27^Kip1^ as transcriptional repressor is to recruit G1 cyclin/Cdk complexes needed for p130 phosphorylation in the early-mid G1 phase^68^. This regulation of a cyclin/Cdk inhibitor at gene promoters is unknown in budding yeast, and a direct involvement of Sic1 as transcriptional repressor, potentially through Fkh2, calls for a detailed experimental investigation, which we are currently conducting in our laboratory.

Importantly, we addressed that the limit cycles we identified belong to multiple different phenotypic regions, in the SDS sense (which sometimes partially overlap), and that the parameter values cover multiple orders of magnitude and are spread across a PCA projection, whose variance is spread across many principal components. Our analysis suggests that it is improbable that all limit cycles found for each particular model design belong to the same robustness region. However, it is currently not possible to prove that their robustness regions do not overlap. In order to answer this question, it would be useful to explore whether methods that approximate single robustness regions^49^ can be applied to multiple limit cycles to prove their separation in the parameter space.

Our work shows how the SDS methodology can aid in the identification of qualitatively distinct behavior of complex systems, resulting in phenotypes that are characterized by a tendency to generate oscillations within a definite network design. In this respect, phenotypes that exhibit oscillations can be considered functional phenotypes of a cell, which exhibit oscillations or a high number of oscillations as compared to dysfunctional phenotypes, which can exhibit no or low amount of oscillations. This approach may represent an interesting avenue of further research, to embed the missing details of the minimal cell cycle network considered in this work into existing checkpoint models. Functional and dysfunctional cellular states may be viewed as (different) stable attractors, i.e. some alteration has been introduced in the functional biochemical network that gives rise to a different attractor that is dysfunctional^69^. The cell cycle network incorporates designs that are particularly suited to support sustained oscillations, suggesting that this biochemical process may have evolved to generate or stabilize autonomous oscillations, at least under some conditions.

We envision that populations of cells consist of subpopulations expressing different phenotypes, and that individual cells are able to dynamically shift their network configurations so as to effectively alter their phenotype. This would allow evolutionarily selectable differences between subpopulations to emerge. Point mutations and shifts in gene expression, e.g. up- and down-regulation of inhibitors, provide valid mechanisms by which the functioning of any network interaction could be altered. Such alterations can impact the strength of, or entirely block, a network interaction, e.g. a PFL. For example, in our cell cycle networks, binding or phosphorylation affinity of the Clb/Cdk1 complexes could be altered. Consequently, cells may theoretically be able to dynamically shift network configurations, as we have proposed for metabolic networks^70,71^, causing switches in phenotype. Our results indicate that, if these changes occur within the core cell cycle regulatory network, an impact on the ability of the network to exhibit oscillations can be observed. Therefore, differences in the affinity of Clb/Cdk1 complexes to bind and phosphorylate Fkh transcription factors and, vice versa, in the affinity of Fkh to the *CLB* promoters may be expected.

Given the evolutionary conservation of the cell cycle network across eukaryotes, our approach may be translated to human cell cycle models, in which components are often mutated in disease^72^. In the mammalian cell cycle there does not exist a one-to-one relationship between the robustness and maintained frequency of the cell cycle of individual cells on the one hand, and the “health” of the whole organism on the other hand. For human cells, the whole organism may not be “healthy” when each cell would cycle robustly, or with a higher frequency. Here our approach reverses: in the context of cancer, our analysis could highlight network design principles that would be good (healthy) for the particular disease state (the cancer). Regardless, applied to the whole organism or to populations of cells within the organism that may become deregulated, as in cancer, it is of interest to identify network properties that result in changes in the robustness and frequency of oscillations. Thus, our pipeline can be used to point to precise molecular strategies of intervention to restore molecular designs that may be disrupted in disease.

## Methods

All Python and MATLAB code, Jupyter Notebooks and COPASI files are available as part of a Github repository (https://github.com/barberislab/Autonomous_Minimal_Cell_Cycle_Oscillator).

### Simulation of ODE models

Time course analyses were conducted in MATLAB 2017a by using the ode15s solver or in Python 2.7 by using the scientific python (SciPy) version 1.1.0, Numeric Python (NumPy) v1.14.1, Design Space Toolbox Python module v0.3.0a4, and the related C toolbox v0.3.0a6. In the Python scripts, a sequence of integrators was set up in case that one of the methods would fail to integrate accurately. The sequence implemented was the following: lsoda, bdf, and dopri. The Python and MATLAB code used to generate all our analyses are provided in the Supplementary Code Repository.

### Finding oscillatory phenotypes by using the Systems Design Space Toolbox

The Design Space Toolbox V2 for Python 2.7 (ref. ^31^) was used to apply the SDS methodology to the 11 network designs considered in this work. The functionality of the toolbox was first tested by reproducing previously published results by Savageau and colleagues^27^. Subsequently, their pipeline to analyze the phenotypic repertoire for oscillatory phenotypes was implemented in Python. The uniqueness of our implementation is two-fold: (i) the generalization of this pipeline, to be applicable to any predefined kinetic model in terms of a set of equations in SciPy notation; (ii) the approach of Savageau and colleagues was improved to include extensive parameter sampling within the boundaries of phenotypes characterized by potential oscillations (oscillatory phenotypes), i.e. areas in the parameter space with two non-negative eigenvalues. Our computational pipeline is shown in Fig. 2. The pipeline consists of a Python script containing model definitions, a set of newly written Python functions that are wrapped around the Design Space Toolbox V2, and several Jupyter notebooks^73^ that analyze a model and properties of any given limit cycle, respectively. All files are available in the Supplementary Code Repository. The main Jupyter notebook reads the model to be analyzed (defined in GMA form), and then proceeds to (i) set up the design space for the model, (ii) identify all valid phenotypes, (iii) identify the stability of each valid phenotype indicated by the presence of two complex conjugate eigenvalues with non-negative real part, by sampling each valid phenotype for a user defined number of times (in this study 250 parameter samples were collected), and (iv) retrieve limit cycle behavior by integrating the full kinetic model in the GMA form (Supplementary Information, Section 4) for the sampled parameter set. The other Jupyter notebooks allow users (i) to analyze properties of a limit cycle, specifically to draw: 1D bifurcation diagrams, phenotype phase planes, stability diagrams, and robustness regions for a user defined set of limit cycles and bifurcation parameters; (ii) to reproduce the results from Tables 1 and 2; and (iii) to reproduce the boxplots of the period, amplitude, parameter values and the PCA plots.

### Sampling of the parameter space to identify oscillatory phenotypes and limit cycles

The phenotypes characterized by a network design may theoretically exhibit oscillatory as well as steady-state behaviors for a range of parameter values. To determine the steady state(s) of a phenotype, a representative point within the phenotypic region of the parameter space must be found. The Design Space Toolbox can determine valid parameter sets for a given phenotype. The log-linear boundaries associated with the boundary conditions for the system (referred to as an S-system) representing a particular phenotype define a continuous subspace of parameter values. Within this space, the terms in the S-system dominate the neglected terms and describe the dominant behavior (Supplementary Information, Section 4). These boundaries enable linear programming problems to be solved to identify a set of parameter values at a vertex of the phenotypic region.

We implemented a sampling approach in two steps. Since steady-state stability may change within a phenotype when model parameters are altered, sampling just once may not give an accurate view of a phenotype’s stability. We sampled parameter sets for each valid phenotype of a particular network design 250 times. For each sample of the parameter values, we first used the functionality of the Systems Design Space Toolbox to determine the steady-state and the presence of non-negative real part complex conjugate eigenvalues of the steady-state for the combination of a given phenotype and parameter set. Second, for valid phenotypes satisfying the necessary condition for sustained oscillations, i.e. two complex conjugate non-negative eigenvalues at the fixed point, we determined the dynamics in the full model in the GMA form, using the previously calculated steady-state as the initial condition, and checked for the presence of a limit cycle. Different initial conditions may give rise to different attractors and, hence, iterating this procedure for multiple initial conditions may result in the identification of more limit cycle attractors. However, in this work, we did not take this approach.

To sample a valid parameter set for a given phenotype, we first used the *valid_parameter_set* function in the Design Space Toolbox to obtain a valid parameter set for the phenotype. We then rearranged the parameters in the model by shuffling them into a random in order so as to avoid biased sampling due to the fact that the sample for each parameter may alter the phenotypic boundaries for the following parameters. Subsequently, for each parameter in the randomized order, we (i) determined the phenotypic tolerance: 1D boundaries of the phenotype when keeping all other parameters the same (utilizing the *vertices_1D_slice* function in the Systems Design Space Toolbox), and (ii) log-uniformly sampled the range of numbers between these boundaries. This sequence of steps ensures that we retrieve a set of unique parameter sets that are specific for a given phenotype, and random. We opted for log-uniform random sampling due to inherent problems that we observed with uniform random sampling. In uniform random sampling in 1D, ranges with the same length have the same probability of being sampled. When a phenotype has, for example, a range of [0, 100], 99% of the samples will fall in the [1, 100] interval and 10% will fall in the [90 to 100] interval. This has the consequence that parameter sets with relatively low parameter values are exceedingly rare, especially when sampling multiple parameters simultaneously as is commonly the case with biochemical models. This problem is further aggravated by the fact that the effects of parameters in the model are multiplicative, rather than additive. As do Metabolic Control Analysis (MCA) and Biochemical Systems Theory (BST), we think that equal relative changes are equally important; hence, we concluded that log-uniform sampling was appropriate for this work. In all model designs, parameters were limited to the range [10^−9^, 1000].

To check whether sampled parameter sets yield limit cycles, and not just damped oscillations, we integrated the system of ODEs for the full model in the GMA form (Supplementary Information, Section 4) in a series of subsequent time windows. After each successive time window we first checked whether the integration of the ODEs proceeded successfully without error. Second, we used the last time window to check the properties of the time course for each of the model species (Sic1, Clb5, Clb3, Clb2) by identifying all maxima that: (i) were within 5% of the global maxima in the current time window. When five such ordered maxima for each species occurred in the time course, we considered the time course to exhibit sustained oscillations. A limit cycle trajectory exhibits multiple, successive peaks in a repeating pattern and would therefore satisfy the aforementioned criteria. The five ordered maxima of each species may represent a yeast cell dividing at least five times. To accept the time course as a limit cycle, we additionally required that: (i) all species have an oscillation amplitude of at least 10% of their global maximum, (ii) the ratio between the global maxima across all species is less than 100-fold (experimentally, there is less than a three-fold difference in the concentration of the four species considered in the model^50,51^), and (iii) the identified maxima in step (ii) are not only found in the beginning of the time course, since this would otherwise indicate a damped oscillation. The main results presented in Table 2 did not change when we required an oscillation amplitude of at least 50%. After each successive time integration window, we checked that the conditions above were met. If the limit cycle conditions were satisfied, the integration was stopped. Finally, as last criteria, we required the identified limit cycle oscillation to exhibit the correct cell cycle order (Sic1, Clb5, Clb3, Clb2). Conversely, if the conditions were not satisfied, the integration was continued unless a steady-state had been reached. We defined a steady-state as when none of the concentrations changed more than 1% of their global maxima in the last time window. If the time course did not exhibit a limit cycle or a steady-state within 10,000 min, the integration was stopped and we concluded that the time course did not exhibit oscillations. In our hands, these two tests are sufficient to identify limit cycles. In rare cases, this approach may erroneously detect a limit cycle although there is in fact a slowly decaying, damped oscillation or slowly increasing oscillations; however, such cases should become clearer from inspection of the time course.

### Principal Component Analysis

PCA projects a set of N-dimensional vectors along a new coordinate axis specified by orthogonal and uncorrelated vectors which are ranked according to how much of the variance in the data they explain. These “principal components” are linear combinations of the original parameters and are of unit length. Each principal component is associated with a percentage of the variance in the original dataset that it explains. As a result we can visualize features (parameter values in this case) in two-dimensional space in such a way that parameter sets that are “close together” (i.e. not showing much variation) will appear together on the PCA plot.

Observations that are far apart in the PCA plot are separated in the original space along the directions specified by the principal components if there was significant deviation within the original dataset to begin with. However, parameter sets that are far apart in the original data may sometimes cluster closely together on the PCA plots if the first principal components do not explain a lot of the variance in the dataset.

We first normalized the parameter sets by dividing by the standard deviation and subtracting the mean so that each parameter has a mean of 0 and a variance of 1 across the different limit cycle parameter sets before applying the PCA algorithm. This helps avoid bias from the multiple orders of magnitude covered in the parameter values.

### Reporting summary

Further information on research design is available in the Nature Research Reporting Summary linked to this article.

## Supplementary information

Supplementary Information

Reporting Summary

## Data Availability

Data sharing not applicable to this article as no datasets were generated or analyzed during the current study.

## References

[1] Isaeva VV (2012). Self-organization in biological systems. Biol. Bull. Russ. Acad. Sci..

[2] Hess B, Mikhailov A (1994). Self-organization in living cells. Science.

[3] Richard P, Bakker BM, Teusink B, Van Dam K, Westerhoff HV (1996). Acetaldehyde mediates the synchronization of sustained glycolytic oscillations in populations of yeast cells. Eur. J. Biochem..

[4] Danø S, Sørensen PG, Hynne F (1999). Sustained oscillations in living cells. Nature.

[5] Ferrell JE, Tsai TY-C, Yang Q (2011). Modeling the cell cycle: why do certain circuits oscillate?. Cell.

[6] Murray AW, Kirschner MW (1989). Dominoes and clocks: the union of two views of the cell cycle. Science.

[7] Tyson JJ, Csikasz-Nagy A, Novak B (2002). The dynamics of cell cycle regulation. Bioessays.

[8] Gérard C, Goldbeter A (2009). Temporal self-organization of the cyclin/Cdk network driving the mammalian cell cycle. Proc. Natl Acad. Sci. USA.

[9] Gérard C, Goldbeter A (2012). From quiescence to proliferation: Cdk oscillations drive the mammalian cell cycle. Front. Physiol..

[10] Barberis M (2012). Sic1 plays a role in timing and oscillatory behaviour of B-type cyclins. Biotechnol. Adv..

[11] Barberis M, Klipp E, Vanoni M, Alberghina L (2007). Cell size at S phase initiation: an emergent property of the G1/S network. PLoS Comput. Biol..

[12] Steuer R (2004). Effects of stochasticity in models of the cell cycle: from quantized cycle times to noise-induced oscillations. J. Theor. Biol..

[13] Gérard C, Gonze D, Goldbeter A (2012). Effect of positive feedback loops on the robustness of oscillations in the network of cyclin-dependent kinases driving the mammalian cell cycle. FEBS J..

[14] Ferrell JE (2013). Feedback loops and reciprocal regulation: recurring motifs in the systems biology of the cell cycle. Curr. Opin. Cell Biol..

[15] Ananthasubramaniam B, Herzel H (2014). Positive feedback promotes oscillations in negative feedback loops. PLoS ONE.

[16] Thomas, R. in *Numerical Methods in the Study of Critical Phenomena*, Springer Series in Synergetics (eds Della Dora, J., Demongeot, J. & Lacolle, B., eds), Vol 9, pp 180–193 (Springer, Berlin, Heidelberg, 1981).

[17] Rangarajan N, Fox Z, Singh A, Kulkarni P, Rangarajan G (2015). Disorder, oscillatory dynamics and state switching: the role of c-Myc. J. Theor. Biol..

[18] Moore JD (2013). In the wrong place at the wrong time: does cyclin mislocalization drive oncogenic transformation?. Nat. Rev. Cancer.

[19] Cookson NA, Cookson SW, Tsimring LS, Hasty J (2010). Cell cycle-dependent variations in protein concentration. Nucleic Acids Res..

[20] Chen KC (2004). Integrative analysis of cell cycle control in budding yeast. Mol. Biol. Cell.

[21] Chen KC (2000). Kinetic analysis of a molecular model of the budding yeast cell cycle. Mol. Biol. Cell.

[22] Doedel E (1991). Numerical analysis and control of bifurcation problems (I): bifurcation in finite dimensions. Int. J. Bifurcat. Chaos.

[23] Chickarmane V, Paladugu SR, Bergmann F, Sauro HM (2005). Bifurcation discovery tool. Bioinformatics.

[24] Levering J, Kummer U, Becker K, Sahle S (2013). Glycolytic oscillations in a model of a lactic acid bacterium metabolism. Biophys. Chem..

[25] Savageau MA, Coelho PM, Fasani RA, Tolla DA, Salvador A (2009). Phenotypes and tolerances in the design space of biochemical systems. Proc. Natl Acad. Sci. USA.

[26] Lomnitz JG, Savageau MA (2013). Phenotypic deconstruction of gene circuitry. Chaos.

[27] Lomnitz JG, Savageau MA (2015). Elucidating the genotype–phenotype map by automatic enumeration and analysis of the phenotypic repertoire. NPJ Syst. Biol. Appl..

[28] Hilbert D (1902). Mathematical Problems. Bull. Am. Math. Soc..

[29] Battogtokh D, Tyson JJ (2004). Bifurcation analysis of a model of the budding yeast cell cycle. Chaos.

[30] Gérard C, Tyson JJ, Coudreuse D, Novák B (2015). Cell cycle control by a minimal Cdk network. PLoS Comput. Biol..

[31] Lomnitz JG, Savageau MA (2016). Design Space Toolbox V2: automated software enabling a novel phenotype-centric modeling strategy for natural and synthetic biological systems. Front. Genet..

[32] Linke C (2017). A Clb/Cdk1-mediated regulation of Fkh2 synchronizes CLB expression in the budding yeast cell cycle. NPJ Syst. Biol. Appl..

[33] Pic-Taylor A, Darieva Z, Morgan BA, Sharrocks AD (2004). Regulation of cell cycle-specific gene expression through cyclin-dependent kinase-mediated phosphorylation of the forkhead transcription factor Fkh2p. Mol. Cell. Biol..

[34] Bloom J, Cross FR (2007). Multiple levels of cyclin specificity in cell-cycle control. Nat. Rev. Mol. Cell Biol..

[35] Cross FR, Yuste-Rojas M, Gray S, Jacobson MD (1999). Specialization and targeting of B-type cyclins. Mol. Cell.

[36] Donaldson AD (1998). CLB5-dependent activation of late replication origins in S. cerevisiae. Mol. Cell.

[37] Richardson H, Lew DJ, Henze M, Sugimoto K, Reed SI (1992). Cyclin-B homologs in Saccharomyces cerevisiae function in S phase and in G2. Genes Dev..

[38] Pecani K, Cross FR (2016). Degradation of the mitotic Cyclin Clb3 is not required for mitotic exit but is necessary for G1 cyclin control of the succeeding cell cycle. Genetics.

[39] Schwob E, Nasmyth K (1993). CLB5 and CLB6, a new pair of B cyclins involved in DNA replication in Saccharomyces cerevisiae. Genes Dev..

[40] Fitch I (1992). Characterization of four B-type cyclin genes of the budding yeast Saccharomyces cerevisiae. Mol. Biol. Cell.

[41] Dahmann C, Futcher B (1995). Specialization of B-type cyclins for mitosis or meiosis in S. cerevisiae. Genetics.

[42] Cross FR, Schroeder L, Bean JM (2007). Phosphorylation of the Sic1 inhibitor of B-type cyclins in Saccharomyces cerevisiae is not essential but contributes to cell cycle robustness. Genetics.

[43] Fasani RA, Savageau MA (2010). Automated construction and analysis of the design space for biochemical systems. Bioinformatics.

[44] Amon A, Tyers M, Futcher F, Nasmyth K (1993). Mechanisms that help the yeast cell cycle clock tick: G2 cyclins transcriptionally activate G2 cyclins and repress G1 cyclins. Cell.

[45] Skotheim JM, Di Talia S, Siggia ED, Cross FR (2008). Positive feedback of G1 cyclins ensures coherent cell cycle entry. Nature.

[46] Verma R (1997). Phosphorylation of Sic1p by G1 Cdk required for its degradation and entry into S phase. Science.

[47] Moll T, Tebb G, Surana U, Robitsch H, Nasmyth K (1991). The role of phosphorylation and the CDC28 protein kinase in cell cycle-regulated nuclear import of the S. cerevisiae transcription factor SWI5. Cell.

[48] Rahi SJ, Pecani K, Ondracka A, Oikonomou C, Cross FR (2016). The CDK-APC/C oscillator predominantly entrains periodic cell-cycle transcription. Cell.

[49] Apri M, Molenaar J, de Gee M, van Voorn G (2010). Efficient estimation of the robustness region of biological models with oscillatory behavior. PLoS ONE.

[50] Cross FR, Archambault A, Miller M, Klovstad M (2002). Testing a mathematical model of the yeast cell cycle. Mol. Biol. Cell.

[51] Ghaemmaghami S (2003). Global analysis of protein expression in yeast. Nature.

[52] Kaizu K (2010). A comprehensive molecular interaction map of the budding yeast cell cycle. Mol. Syst. Biol..

[53] Barberis M (2012). Sic1 as a timer of Clb cyclin waves in the yeast cell cycle–design principle of not just an inhibitor. FEBS J..

[54] Epstein CB, Cross FR (1992). CLB5: a novel B cyclin from budding yeast with a role in S phase. Genes Dev..

[55] Ikui AE, Cross FR (2009). Specific genetic interactions between spindle assembly checkpoint proteins and B-Type cyclins in Saccharomyces cerevisiae. Genetics.

[56] Kuczera T, Bayram Ö, Sari F, Braus GH, Irniger S (2010). Dissection of mitotic functions of the yeast cyclin Clb2. Cell Cycle.

[57] Surana U (1991). The role of CDC28 and cyclins during mitosis in the budding yeast S. cerevisiae. Cell.

[58] Ball DA (2011). Stochastic exit from mitosis in budding yeast: model predictions and experimental observations. Cell Cycle.

[59] Barberis M (2011). A low number of SIC1 mRNA molecules ensures a low noise level in cell cycle progression of budding yeast. Mol. Biosyst..

[60] Ball DA (2011). Oscillatory dynamics of cell cycle proteins in single yeast cells analyzed by imaging cytometry. PLoS ONE.

[61] Ball DA (2013). Measurement and modeling of transcriptional noise in the cell cycle regulatory network. Cell Cycle.

[62] Barik D, Ball DA, Peccoud J, Tyson JJ (2016). A stochastic model of the yeast cell cycle reveals roles for feedback regulation in limiting cellular variability. PLoS Comput. Biol..

[63] Lomnitz JG, Savageau MA (2014). Strategy revealing phenotypic differences among synthetic oscillator designs. ACS Synth. Biol..

[64] Heldt FS, Lunstone R, Tyson JJ, Novák B (2018). Dilution and titration of cell-cycle regulators may control cell size in budding yeast. PLoS Comput. Biol..

[65] Rata S (2018). Two interlinked bistable switches govern mitotic control in mammalian cells. Curr. Biol..

[66] Barberis M (2005). The yeast cyclin-dependent kinase inhibitor Sic1 and mammalian p27Kip1 are functional homologues with a structurally conserved inhibitory domain. Biochem. J..

[67] Pippa R (2012). p27Kip1 represses transcription by direct interaction with p130/E2F4 at the promoters of target genes. Oncogene.

[68] Orlando S (2015). p27Kip1 and p21Cip1 collaborate in the regulation of transcription by recruiting cyclin-Cdk complexes on the promoters of target genes. Nucleic Acids Res..

[69] Davis JD, Kumbale CM, Zhang Q, Voit EO (2019). Dynamical systems approaches to personalized medicine. Curr. Opin. Biotechnol..

[70] Abudukelimu A, Mondeel TDGA, Barberis M, Westerhoff HV (2017). Learning to read and write in evolution: from static pseudoenzymes and pseudosignalers to dynamic gear shifters. Biochem. Soc. Trans..

[71] Mondeel, T. D. G. A. et al. Maps for when the living gets tough: maneuvering through a hostile energy landscape. *IFAC-PapersOnLine* 49, 364–370 (2016).

[72] Weinberg, R. A. *The Biology of Cancer* 2nd edn, Ch. 8, 320 (Garland Science, Taylor & Francis Group, New York and London, 2014).

[73] Kluyver, T. et al. in *Positioning and Power in Academic Publishing: Players, Agents and Agendas* (eds Loizides, F. & Scmidt, B.) 87–90 (IOS Press, Amsterdam, 2016).

