# Supplementary material for: Clb3-centered regulations are recurrent across distinct parameter regions in minimal autonomous cell cycle oscillator designs
Source: NPJ Syst Biol Appl. 2020 Apr 3;6:8. doi: 10.1038/s41540-020-0125-0 (PMC7125140; doi:10.1038/s41540-020-0125-0)
Supplement: Supplementary file 1 — Supplementary Information [file 41540_2020_125_MOESM1_ESM.pdf]

Supplementary Information

Clb3-centered regulations are recurrent across distinct parameter regions in minimal autonomous cell cycle oscillator designs

Thierry D.G.A. Mondeel, Oleksandr Ivanov, Hans V. Westerhoff, Wolfram Liebermeister and Matteo Barberis

1 Model derivations and initial limit cycles for *Design 1A* through *Design 3*

In this section, we introduce the model designs considered in our work. The various models are updated and expanded versions of the minimal cell cycle model of Barberis and colleagues [1], shown in **Supplementary Figure 1**. We will start by discussing the original model.

1.1 Notation and assumptions in the minimal cell cycle model

The starting point is our published minimal cell cycle model [1]. We consider a system of seven species: (i) three representing the complexes that Cdk1 forms with the three pairs of B-type cyclins, Clb5,6, Clb3,4 and Clb1,2, which we refer to as x, y and z, and (ii) the inhibitor Sic1, which we refer to as s, that binds and inhibits all three Clb/Cdk1 complexes ($s \cdot x$, $s \cdot y$ and $s \cdot z$). In living cells of budding yeast there is a distinction between the roughly constant concentration of Cdk1 and the varying concentration of Clb cyclins throughout cell cycle progression. This results in varying concentrations of the Clb/Cdk1 complexes; however, this distinction is not needed for the modeling purposes presented here.

The mathematical description of the minimal model is in terms of a system of coupled Ordinary Differential Equations (ODEs). Specifically, the model considers: (i) basal degradation of all four species in their free form; (ii) basal synthesis of the Clb/Cdk1 complexes; (iii) forward activation in the Clb cyclin cascade from one Clb/Cdk1 complex to another through phosphorylation of a transcription factor; (iv) backward inhibition in the Clb cascade from one Clb/Cdk1 complex to another through the Anaphase-Promoting Complex (APC); (v) reversible formation of the ternary complex formed by any of the Clb/Cdk1 complexes and Sic1; (vi) degradation of Sic1 in any of the Clb/Cdk1/Sic1 ternary complexes; and (vii) degradation of Clb cyclins in the Clb/Cdk1/Sic1 ternary complexes. We consider x, y and z to be active in the binary complexes, and inactive in the ternary complexes with s. We also consider that degradation of Sic1 can occur when Sic1 is either free or bound to Clb/Cdk1 in the

Clb/Cdk1/Sic1 complexes. We refer to reference [1] and **Supplementary Table 2** for the experimental evidence of the interactions described above.

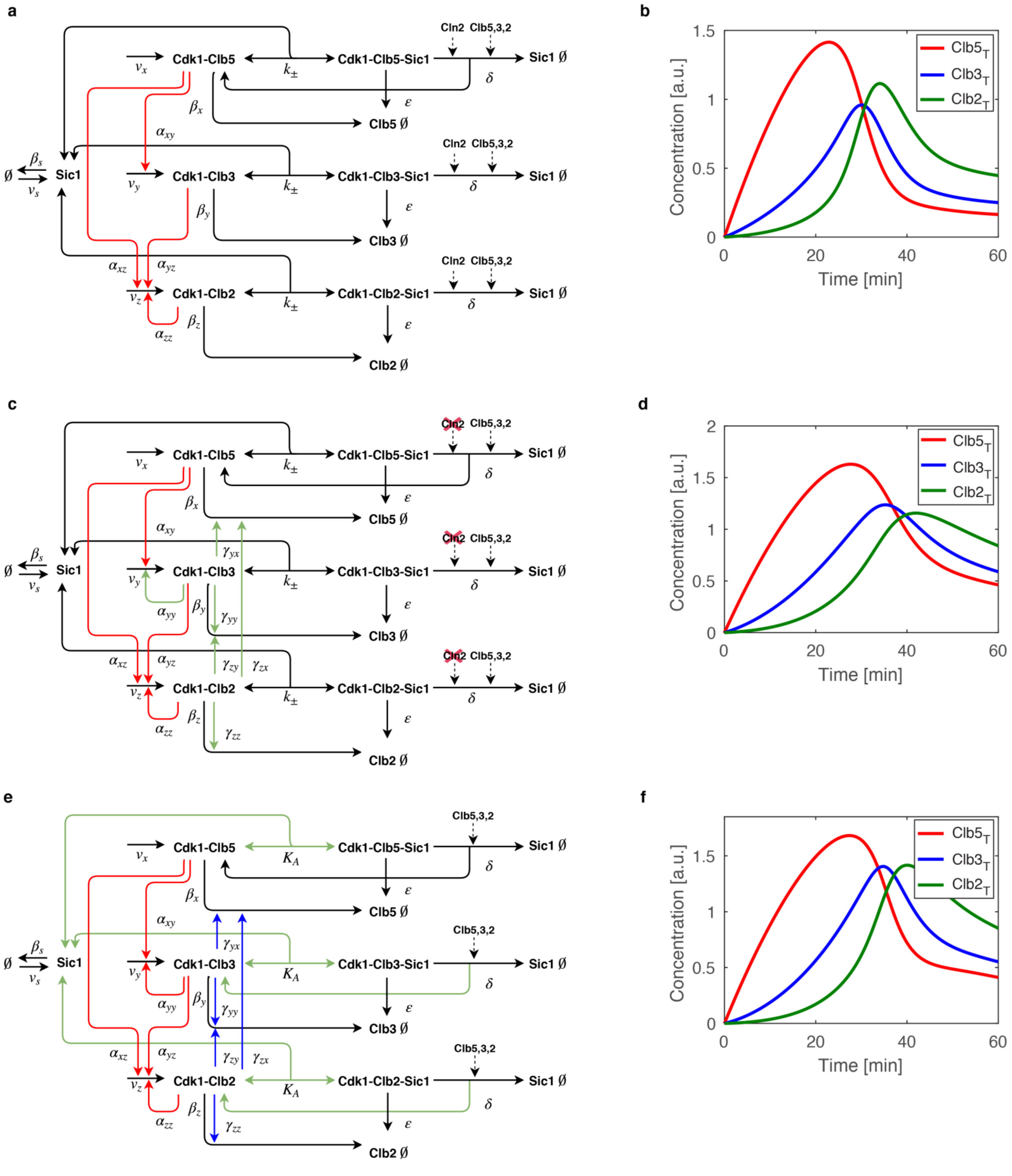

Supplementary Figure 1*.* Alternative designs of a minimal cell cycle network in budding yeast. Model schemes and simulated time courses of waves of Clb/Cdk1 complexes are represented as follows: *Design 1A* (**a**) and its time course (**b**); *Design 1B* and *Design 1C* (**c**) and simulated time course for *Design 1C* (**d**); *Design 2* and *Design 3* (**e**) and simulated time course for *Design 3* (**f**). First, the minimal cell cycle model (*Design 1A*) was expanded to generate *Design 1B* (see Section 1.4) by including: (i) a positive feedback loop (PFL) by Clb3/Cdk1 on *CLB3* (Clb3 PFL); α_{yy}) in **c**) through a transcription factor [2], (ii) a negative feedback loop (NFL) of Clb3/Cdk1 on itself through activation of degradation (Clb3 NFL; γ_{yy} in **c**), and (ii) the four known Clb-regulated degradations (inhibitory regulations) mediated by both the Clb/Cdk1 complexes and Anaphase Promoting Complex (APC) (γ_{zz}, γ_{zy}, γ_{zx} and γ_{yx}) (see Fig. 1C in ref. 1 for details). *Design 1C* removes the Cln2/Cdk1-mediated contribution to Sic1 degradation from the Clb/Cdk1/Sic1 ternary complexes (red crosses in **c**). *Design 2* includes the salvaging of Clb3/Cdk1 and Clb2/Cdk1 upon degradation of Sic1 from the Clb/Cdk1/Sic1 ternary complexes (see green lines in **e** indicated with δ) (see Section 1.5; such salvaging was already in place for Clb5/Cdk1 upon degradation of the Cdk1-Clb5-Sic1 ternary complex in **a** and **c**). *Design 3* describes the complex formation between Sic1 and Clb cyclins by the association equilibrium constant (K_A), instead of the forward (k_+) and backward (k_-) parameters in *Design 2*, as a consequence of the quasi-steady-state approximation, i.e. assumes a fast equilibrium between the free forms of Sic1 and Clb/Cdk1 complexes and the ternary complexes they form, which is reflected by a high K_A value. This implies that steady-state concentration of Clb/Cdk1/Sic1 ternary complexes changes with Sic1 dynamics, and that the fraction of free Clb cyclins is directly related to free Sic1 at any moment (Supplementary Information, Section 1.6). This assumption is supported by *in vitro* experimental evidence indicating a strong binding of Sic1 to the Clb/Cdk1 complexes. (**a**, **c** and **e**) Red arrows indicate activation of each *CLB* gene, thereby of each Clb/Cdk1 complex, by any previous Clb/Cdk1 complex in the cascade; blue arrows indicate APC-mediated Clb inhibition by Clb/Cdk1 complexes, resulting in Clb degradation; black arrows indicate all other reactions; and green arrows indicate the progressive changes with respect to the previous kinetic model(s). Dotted arrows indicate the Cln(/Cdk1)- and Clb(/Cdk1)-mediated phosphorylation of Sic1 in Clb/Cdk1/Sic1 ternary complexes, resulting in its degradation. (**b**, **d** and **f**) Time courses representing the total concentrations of Clb5, Clb3 and Clb2 as function of time are shown in red, blue and green, respectively (for sake of clarity, Sic1 time course has been omitted). Time courses were obtained by simulating the kinetic models with the canonical parameter set (see Supplementary Table 1).

We assume basal synthesis (v) of each Clb/Cdk1 complex to be at a constant rate, and basal protein degradation (β) to be proportional to the current concentration of a species. We further implement that activation (α) of one Clb/Cdk1 complex by the previous one occurs through activation of a transcription factor, and is thus assumed to be proportional to the activating Clb/Cdk1 complex. Inhibition (γ) from a Clb/Cdk1 complex to the previous one is considered to occur through the APC, and is thus assumed to be proportional to the product of both species involved. Complex formation (k^+) and dissociation (k^-) are proportional to the concentrations of the species forming the complex and to the concentration of the complex, respectively. Complex dissociation due to Sic1 or Clb degradation from the Clb/Cdk1/Sic1 ternary complexes (δ and ϵ, respectively) is assumed to be proportional to the complex concentration. In our mathematical notation, we will use brackets to denote concentrations and the $A \cdot B$ notation to denote a complex formed by two species A and B. In the equations, we neglect to mention Cdk1, as it is most abundant in living cells as compared to the Clb cyclins that activate it, thus being the limiting species.

1.2 The Barberis 2012 model

In terms of the notation introduced above, the model from [1] may be represented as follows:

$$\frac{d[x]}{dt} = v_x - \beta_x[x] - \gamma_{yx}[x][y] - \gamma_{zx}[x][z] - k_x^+[s][x] + k_x^-[s \cdot x] + \delta_x(1 + [x] + [y] + [z])[s \cdot x]$$

$$\frac{d[y]}{dt} = v_y - \beta_y[y] + \alpha_{xy}[x] - \gamma_{zy}[z][y] - k_y^+[s][y] + k_y^-[s \cdot y]$$

$$\frac{d[z]}{dt} = v_z - \beta_z[z] + \alpha_{zz}[z] - \gamma_{zz}[z]^2 + \alpha_{xz}[x] + \alpha_{yz}[y] - k_z^+[s][z] + k_z^-[s \cdot z]$$

$$\frac{d[s]}{dt} = -\beta_s[s] - \left(k_x^+[x] + k_y^+[y] + k_z^+[z]\right)[s] + k_x^-[s \cdot x] + k_y^-[s \cdot y] + k_z^-[s \cdot z]$$

$$\frac{d[s \cdot x]}{dt} = k_x^+[s][x] - k_x^-[s \cdot x] - \delta_x(1 + [x] + [y] + [z])[s \cdot x] - \epsilon_x[s \cdot x]$$

$$\frac{d[s \cdot y]}{dt} = k_y^+[s][y] - k_y^-[s \cdot y] - \delta_y(1 + [x] + [y] + [z])[s \cdot x] - \epsilon_y[s \cdot y]$$

$$\frac{d[s \cdot z]}{dt} = k_z^+[s][z] - k_z^-[s \cdot z] - \delta_z(1 + [x] + [y] + [z])[s \cdot z] - \epsilon_z[s \cdot z]$$

1.3 The canonical parameter set from the Barberis 2012 model

Supplementary Table 1 shows the parameter notation from the original model and the translation to the notation used in this text. The parameter values are those used in the original publication [1].

Supplementary Table 1. Parameters of the Barberis 2012 model as originally published. The parameter names refer to those used in this work, with the matching parameter from the original publication in brackets. Note that some parameters might seem to have different values than in the original publication. This is due to the fact that, originally, the positive regulations were multiplied by basal synthesis, and the negative regulations were multiplied by basal degradation. Here, we incorporate these multiplications into the equations. All parameters have units of min^{-1} when we adopt the convention that concentrations are dimensionless.

Parameter	Value	Parameter	Value	Parameter	Value
v_x (k_1)	0.1	α_{yz} (k_B)	1	ϵ_x (k_4)	0.01
v_y (k_7)	0.01	α_{zz} (k_D)	0.1	ϵ_y (k_{17})	0.01
v_z (k_9)	0.001	γ_{yx} (k_E)	0.7	ϵ_z (k_{13})	0.01
β_x (k_6)	0.7	γ_{zx} (k_F)	0.7	k_x^+ (k_2)	5
β_y (k_8)	0.7	γ_{zy} (k_G)	0.7	k_y^+ (k_{15})	5
β_z (k_{10})	0.7	γ_{zz} (k_H)	0.7	k_z^+ (k_{11})	5
β_s (k_{26})	0.001	δ_x (k_5)	0.05	k_x^- (k_3)	0.5
α_{xy} (k_A)	1	δ_y (k_{18})	0.05	k_y^- (k_{16})	0.5
α_{xz} (k_C)	0.1	δ_z (k_{14})	0.05	k_z^- (k_{12})	0.5

1.4 *Design 1*

In this work, we start from a model that builds on the Barberis 2012 model (**Supplementary Figure 1A**) but contains more regulations in less parameters. We assume that several parameters are equal across molecular species, i.e. all δ, ϵ, k^+, and k^- parameters are assumed to be equal, allowing us to describe the same regulations with less parameters. This means that we assume rates of complex formation and dissociation, Sic1 degradation from the ternary

complexes and total degradation of ternary complexes to be equal among all the three species. Of note, this procedure reduces the number of parameters from 27 as in the original model to 19, but the model output for the default parameter set in [1] remains the same, since these parameters were already assumed to be equal (see **Supplementary Table 1**).

Recently, Fkh2 was identified as a transcription factor of *CLB3* transcription, and that Clb3 may activate Clb2 [2]. Given that Clb3 may phosphorylate Fkh2, a positive feedback loop through Fkh2 has been envisioned in the model. In the Barberis 2012 model, negative feedback inhibitions among the Clb/Cdk1 complexes were considered; among these, the self-inhibition of M phase cyclins. However, in the model this regulation was only implemented for Clb2. Here, we extend the model to additionally implement the Clb3 self-inhibition. Consequently, our model has two new parameters (α_{yy}, γ_{yy}), increasing the total number of parameters to 21.

The Barberis 2012 model included a term representing the Cln1,2/Cdk1 phosphorylation on Sic1 for its degradation, when the latter is in the Clb/Cdk1/Sic1 ternary complexes. This term was incorporated as a constant parameter, because time-varying concentrations of Cln1,2/Cdk1 were not considered. However, this leads to an issue in the units of the corresponding parameter, which was simultaneously used to indicate the phosphorylation of Sic1 mediated by the Clb/Cdk1 complexes. For the former, δ should have units of 1/time and, for the latter, units of 1/(concentration*time). For this reason, and for simplicity, we now introduce this term as a separate parameter λ (increasing the total number of parameters to 22) with units of 1/time and with the convention that λ=δ.

Of note, the Barberis 2012 model did not include any synthesis of Sic1, instead assuming that Sic1 level is high at the start of a cell cycle and decays throughout the cell cycle, rising again during the M phase to restart the cycle. This means that, by design, the model could not generate multiple oscillations in time since this requires resetting of the Sic1 concentration. However, in this work we are interested to retrieve sustained oscillations in all four molecular species, and for this the synthesis term for Sic1 is required. Therefore, we added the simplest

possible synthesis term for Sic1 in the form the of the parameter v_s which has units of concentration/time. This increases the total number of parameters to 23. With the new parameters just introduced, the set of equations describing the model can be written as follows:

$$\frac{d[x]}{dt} = v_x - \beta_x[x] - \gamma_{yx}[x][y] - \gamma_{zx}[x][z] - k^+[s][x] + k^-[s\cdot x]$$
$$+\delta([x] + [y] + [z])\,[s\cdot x] + \lambda\,[s\cdot x]$$
$$\frac{d[y]}{dt} = v_y - \beta_y[y] + \alpha_{xy}[x] + \alpha_{yy}[y] - \gamma_{zy}[z][y] - \gamma_{yy}[y]^2 - k^+[s][y] + k^-[s\cdot y]$$
$$\frac{d[z]}{dt} = v_z - \beta_z[z] + \alpha_{zz}[z] - \gamma_{zz}[z]^2 + \alpha_{xz}[x] + \alpha_{yz}[y] - k^+[s][z] + k^-[s\cdot z]$$
$$\frac{d[s]}{dt} = v_s - \beta_s[s] - k^+([x] + [y] + [z])[s] + k^-\,([s\cdot x] + [s\cdot y] + [s\cdot z])$$
$$\frac{d[s\cdot x]}{dt} = k^+[s][x] - k^-[s\cdot x] - \delta([x] + [y] + [z])[s\cdot x] - \epsilon[s\cdot x] - \lambda\,[s\cdot x]$$
$$\frac{d[s\cdot y]}{dt} = k^+[s][y] - k^-[s\cdot y] - \delta([x] + [y] + [z])[s\cdot y] - \epsilon[s\cdot y] - \lambda\,[s\cdot y]$$
$$\frac{d[s\cdot z]}{dt} = k^+[s][z] - k^-[s\cdot z] - \delta([x] + [y] + [z])[s\cdot z] - \epsilon[s\cdot z] - \lambda\,[s\cdot z]$$

Of note, the Barberis 2012 model does not salvage Clb3,4/Cdk1 and Clb1,2/Cdk1 when Sic1 is degraded in the Clb/Cdk1/Clb ternary complexes, whereas it does recover Clb5,6/Cdk1, as experimentally demonstrated [3]. This can be seen in the equations from the δ term occurring only for x and not for y and z. Last, upon Clb degradation from the Clb/Cdk1/Clb ternary complexes, Sic1 is not recycled, as it can be seen from the equation for the evolution of $[s]$, since no terms with ϵ occur. Biologically, this means that we assume that Sic1 is not available to function, i.e. it is inactivated due to re-localization to the nucleus.

Since we have the ability to 'turn off' certain interactions by setting the corresponding parameters to 0, here we investigate three different scenarios for *Design 1*: *A*, *B* and *C*. For each scenario, we show a transient oscillation ($v_s = 0$), a limit cycle ($v_s \neq 0$) and we analyze the control on the period of the oscillations.

COPASI [4] was used to find initial parameter sets that yielded sustained cyclin/Cdk oscillations. The slider functionality of COPASI allows for the manual adjustment of one or several model parameters, and for the visualization of the output of a given combination of parameter sets. Alternatively, limit cycles may be found by using the *Manipulate* function in Mathematica or the bifurcation software such as MATCONT [5,6] and XPPAUT [7]. COPASI files for designs *1A–3* are available as Supplementary Code Repository.

Once a parameter set that generated oscillatory behavior had been identified, the control exerted by the model's parameters on the period of the oscillation was analyzed. Control can be quantified by control coefficients: logarithmic derivatives of system properties, e.g. fluxes and concentrations, with respect to kinetic parameters. Summation laws have been derived about control coefficients for parameters with dimension 1/time with respect to both autonomous and forced oscillations [8,9]. Taking log-log derivatives of a period (or any other derivative of a stationary state function) is analogous to the concept of a control coefficient for a steady-state flux or concentration i.e. $C_p^\tau = \partial \log(\tau) / \partial \log(p)$ [10,11]. The sensitivity analysis on limit cycles by control coefficients was conducted by using the PeTTSy toolbox in MATLAB [12]. The model equations in MATLAB were converted in the specific format required by PeTTSy; furthermore, files containing parameters and initial conditions were defined as specified in the PeTTSy manual. With these three files (equations, parameters, initial conditions) the model can be read by PeTTSy. The PeTTSy input files are available as part of the Supplementary Code Repository. Once a model has been imported into PeTTSy, a new parameter set may be defined that generates oscillations. PeTTSy then integrates the equations and returns a time course. At this point, the user may run the *Derivatives* function and will be prompted to accept or reject the solution. According to the PeTTSy documentation it is crucial, for accuracy reasons, to accept only solutions with a log [(condition number)] < 36. Typically, we used 400 time blocks to guarantee that this occurred for the models that we considered. After the calculation of the derivatives, the sensitivity of the period with respect to parameter changes was analyzed.

1.4.1 *Design 1A*: No inhibition through the APC

To start with, we considered the model presented in Fig. 2 of [1] (**Supplementary Figure 1A**). In **Supplementary Figure 2**, the evolution over one cell cycle is plotted by using the equations introduced above, and for the parameter values specified in **Supplementary Table 1**, with all γ parameters, α_{yy} and v_s set equal to 0. **Supplementary Figure 2A** shows a transient oscillation in the total Clb/Cdk1 concentrations that exhibits their sequential rise and (near simultaneous) fall over time. Of note, waves of the total concentrations arise predominantly due to the concentrations of the Clb/Cdk1/Sic1 ternary complexes, although waves can be also observed for the concentration of Clb/Cdk1 complexes (**Supplementary Figure 2B**). With regard to the parameter set, cyclin degradation was considered to be of several orders of magnitude faster when in complex with Cdk1 alone (β) as compared to degradation from the Clb/Cdk1/Sic1 ternary complexes (ε). Biologically, this translates to a small likelihood of cyclin degradation from the ternary complexes.

This model is able to exhibit limit cycles when a shift in the parameter set occurs. When we simply turn on the synthesis of Sic1 by setting v_s equal to 0.3. Dampened transient oscillations are shown in **Supplementary Figure 3**. Sustained oscillations are found when increasing the rate of ternary complex formation from 5 to 20 (see **Supplementary Figure 4**); the sustained oscillation is a limit cycle with a period of roughly 113 minutes.

Sensitivity analysis of the period of oscillations points to a few key parameters controlling the period, namely: β_x, ε, v_s, λ, v_x. The former three parameters yield positive derivatives, whereas the latter two parameters yield negative derivatives (see **Supplementary Figure 5)**. Intriguingly, these parameters controlling the period length are all either basal synthesis or basal degradation rates for various species in the model. Moreover, the parameters referring to the regulations among the Clb/Cdk1 complexes do not control significantly the period length.

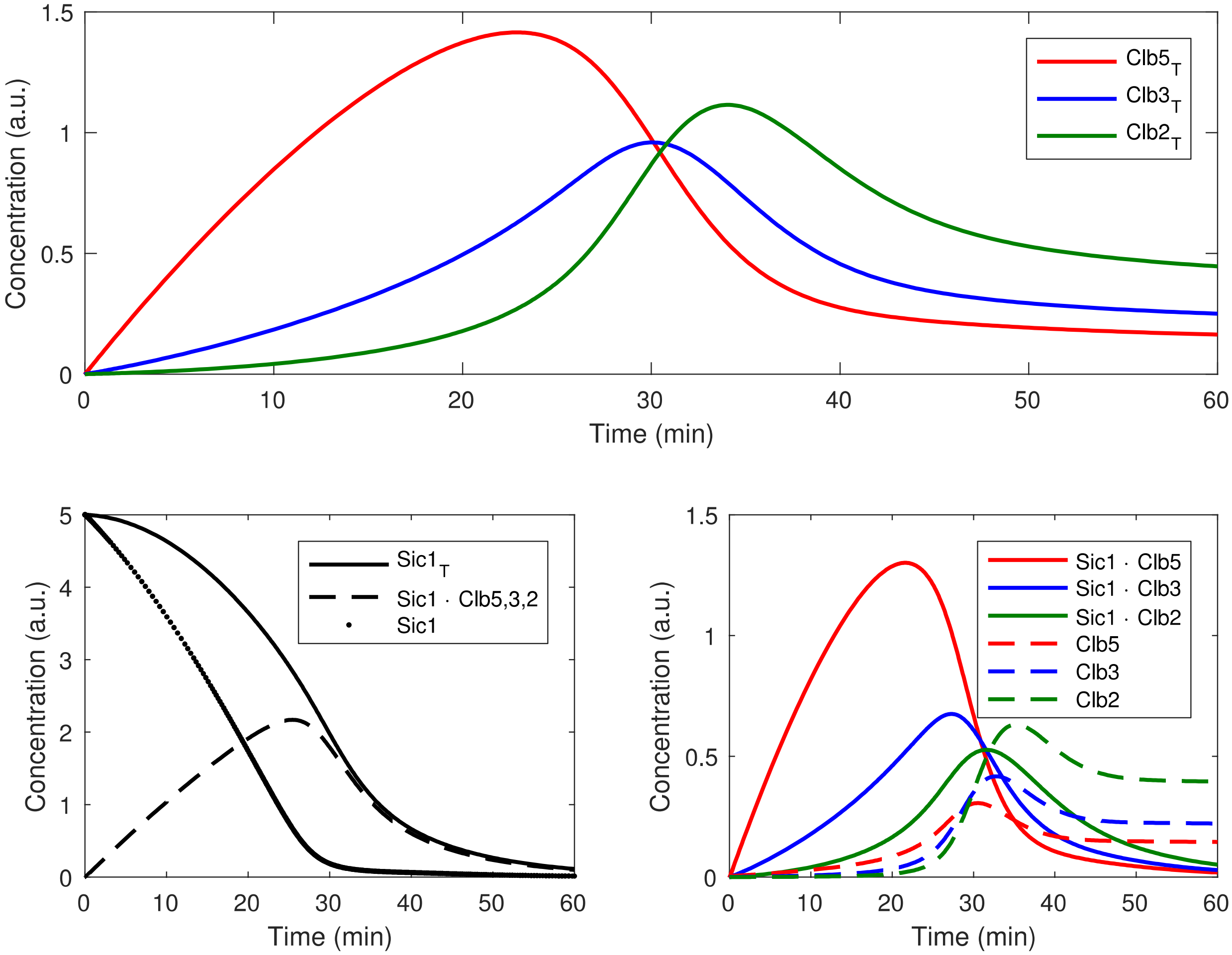

Supplementary Figure 2. Time courses for *Design 1A*. (Top) time courses for the total concentrations of the three Clb/Cdk1 complexes: Clb5/Cdk1, Clb3/Cdk1 and Clb2/Cdk1. (Bottom-left) time courses for total Sic1, Sic1 in complex with Clb/Cdk1 complexes, and Sic1. (Bottom-right) time courses for the binary (Clb/Cdk1) and ternary (Clb/Cdk1/Sic1) complexes.

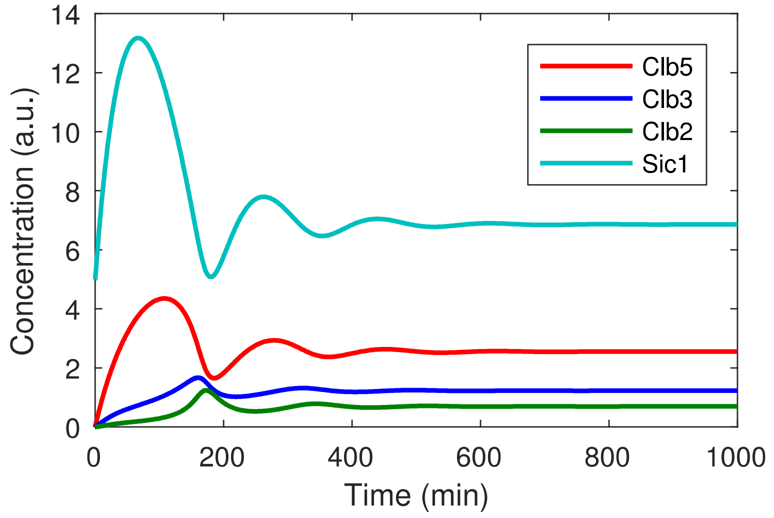

Supplementary Figure 3. Dampened transient oscillations in *Design 1A*, when switching on Sic1 synthesis but keeping all other parameters as in Supplementary Table 1.

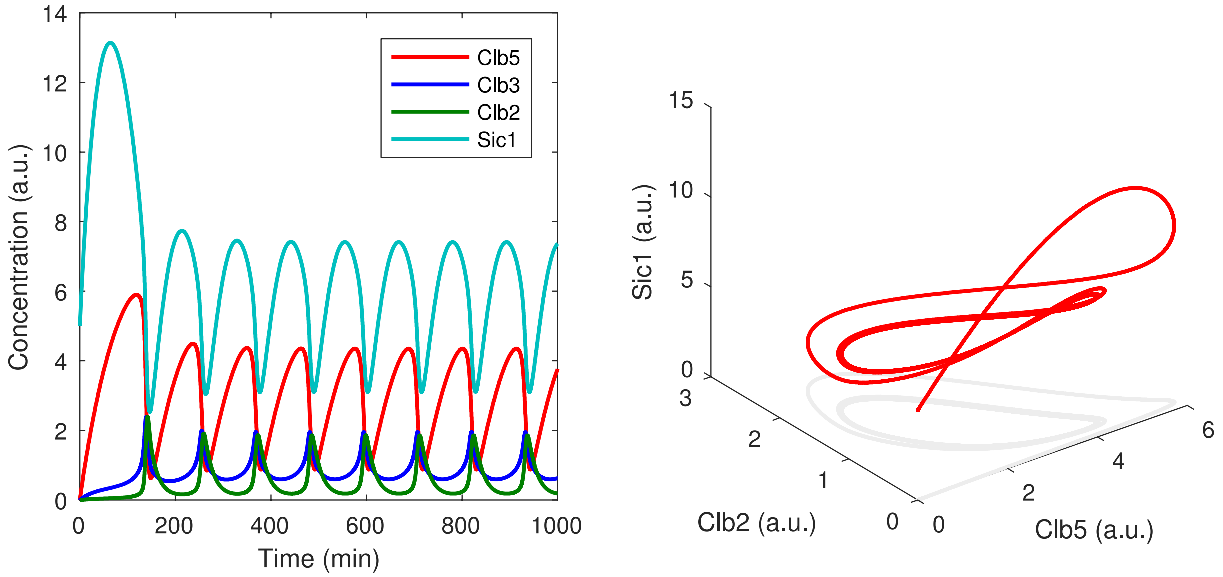

Supplementary Figure 4. Sustained oscillations in *Design 1A*. (Left) Limit cycle for the total concentrations of the four species. (Right) 3D view of the limit cycle in the Clb5-Clb2-Sic1 space.

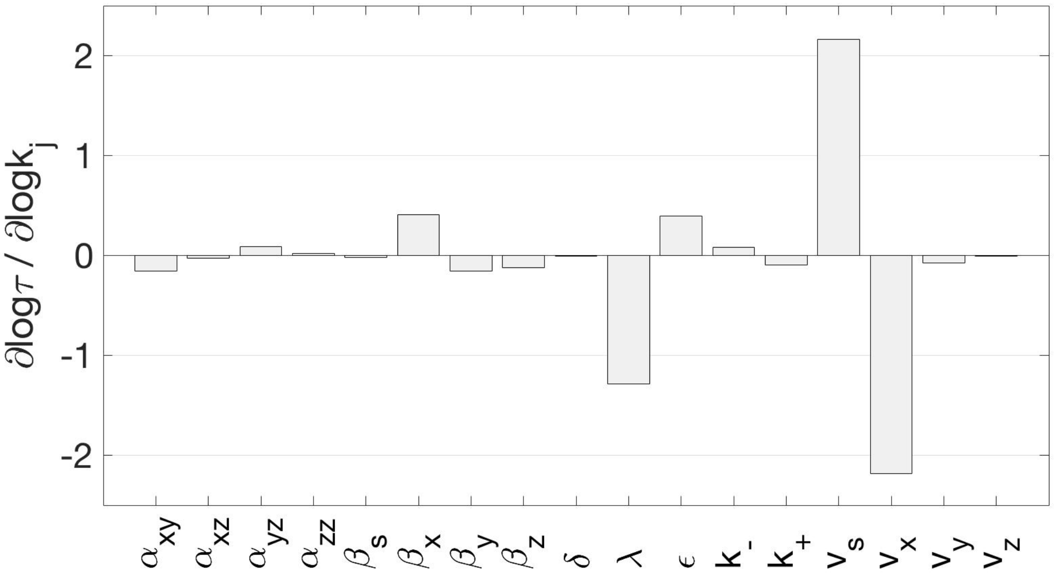

Supplementary Figure 5. Control coefficients of cell cycle times, obtained as logarithmic period derivatives for *Design 1A*. We normalized the derivative by taking the natural logarithm in both the period and the parameter, to end with a measurement of relative change in the period given a relative change in a parameter. This is akin to the definition of control coefficients. The sum of these control coefficients was equal to –1.

1.4.2 *Design 1B*: Regulatory inhibitions and Clb3 positive and negative feedback loops

The *Design 1B* includes the Clb/Cdk1-mediated inhibition on Clb/Cdk1 complexes [13] and the potential of Clb3 self-activation, as we have recently proposed [2] (**Supplementary Figure 1C**). We also include the potential of Clb3 self-inhibition, similarly to that identified on Clb2/Cdk1 [14]. We assume, as we consistently do, that self-activation and self-inhibition are proportional to the protein concentration, in this case y. For this extended model, we set the inhibitory parameters (γ) to a non-zero value, and add self-activation and self-inhibition terms to the Clb3 (y) ODE, where we assume the new parameters to have the values $\alpha_{yy} = 0.1$ and $\gamma_{AB} = 0.7$, i.e. all Clb/Cdk1-mediated inhibitions have the same parameter value as in the Barberis 2012 model (**Supplementary Table 1**). In **Supplementary Figure 6**, the time course for the canonical

parameter is plotted for *Design 1B*. Of note, the peaks of total Clb/Cdk1 concentrations appear slightly earlier as compared to *Design 1A*.

Activating Sic1 synthesis and increasing the Clb/Cdk1/Sic1 ternary complex formation rate, i.e. using the same parameter set as for *Design 1A*, we again find sustained oscillations, now with a period of roughly 95 minutes (see **Supplementary Figure 7**). Of note, this mirrors the anticipation of the Clb/Cdk1 waves in the transient oscillations by a decrease in the limit cycle period. We repeated the sensitivity analysis on the period of the sustained oscillations (see **Supplementary Figure 8**), which show the same qualitatively results as compared to *Design 1A*. Interestingly, none of the newly added inhibitory parameters (γ's) nor γ_{yy} have much control over the period of oscillations.

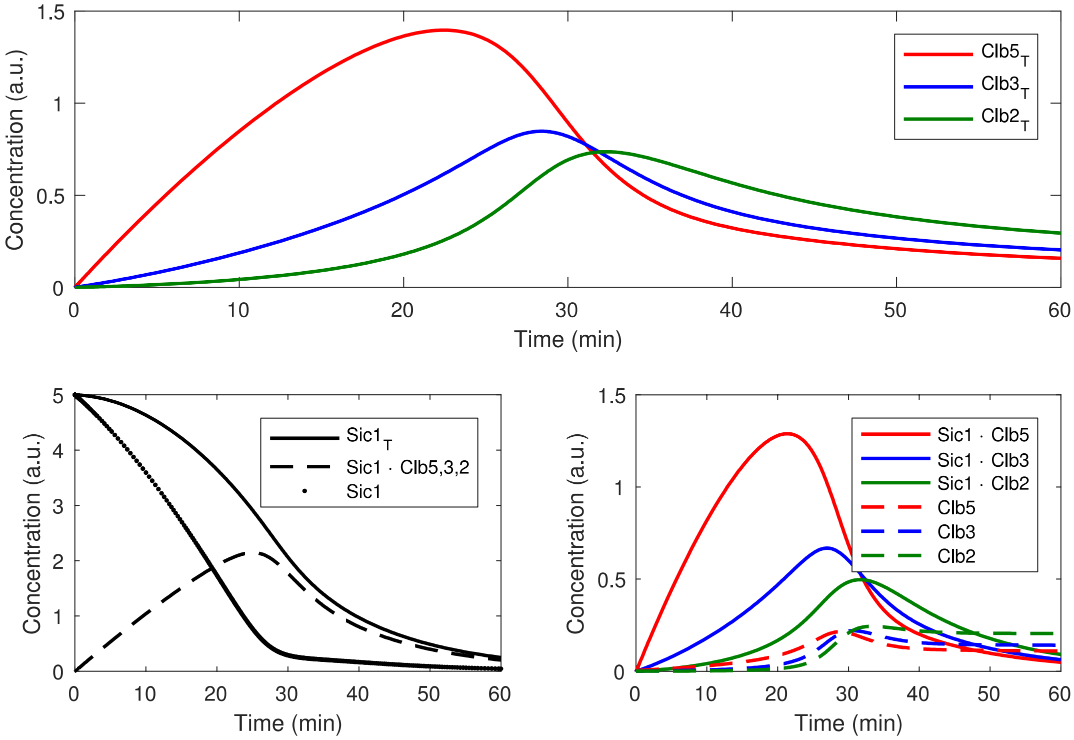

Supplementary Figure 6. Time courses for *Design 1B.* (Top) time courses for the total concentrations of the three Clb/Cdk1 complexes: Clb5/Cdk1, Clb3/Cdk1 and Clb2/Cdk1. (Bottom-left) time courses for total Sic1, Sic1 in complex with Clb/Cdk1 complexes, and Sic1. (Bottom-right) time courses for the binary (Clb/Cdk1) and ternary (Clb/Cdk1/Sic1) complexes.

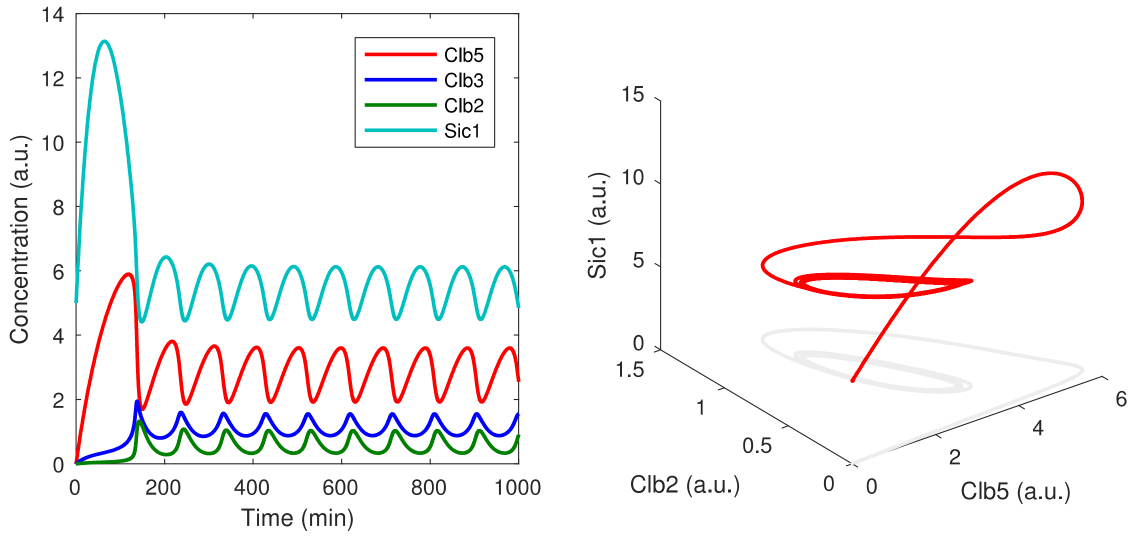

Supplementary Figure 7. Sustained oscillations in *Design 1B*. (Left) Limit cycle for the total concentrations of the four species. (Right) 3D view of the limit cycle in the Clb5-Clb2-Sic1 space.

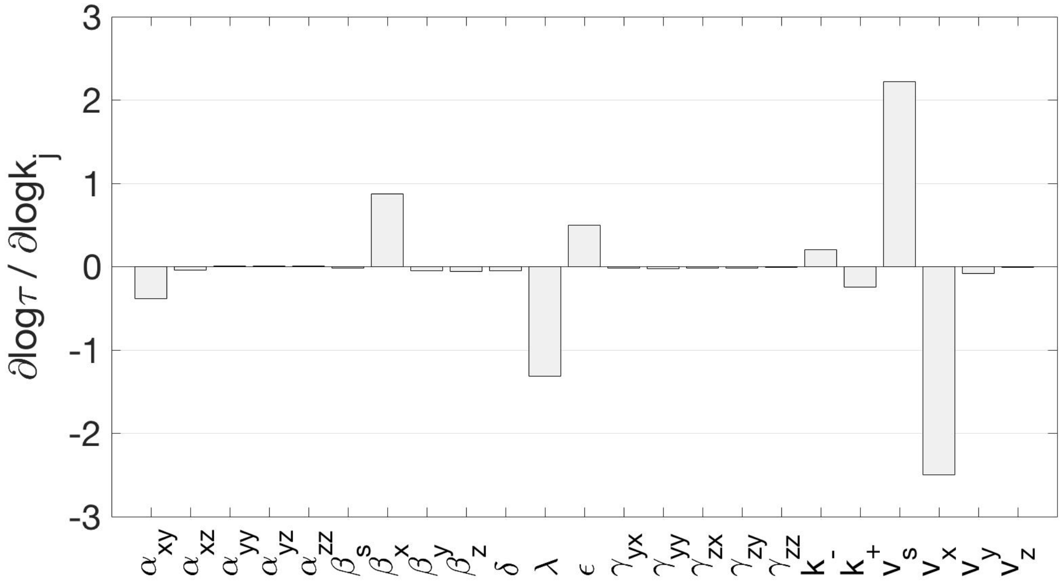

Supplementary Figure 8. Control coefficients of cell cycle times, obtained as logarithmic period derivatives for *Design 1B*.

1.4.3 *Design 1C*: Neglecting Cln1,2/Cdk1 on Sic1 degradation from Clb/Cdk1/Sic1 ternary complexes

To simplify the model further, we neglect the λ parameter, i.e. $\lambda=0$, which was added in the Barberis 2012 model as a way to include basal levels of Sic1 degradation from the Clb/Cdk1/Sic1 ternary complexes (possibly due to Cln1,2/Cdk1) (red crosses in **Supplementary Figure 1C**). However, this parameter does not change the structure of the model, and its effect can be compensated for by ε and δ. In **Supplementary Figure 9**, the transient oscillations for the canonical parameter is plotted for *Design 1C*. Of note, the peaks of total Clb/Cdk1 concentrations appear later as compared to *Design 1B*.

To obtain sustained oscillations in *Design 1C*, a number of parameters had to be altered. Ultimately, autonomous oscillations were obtained varying the following parameters as compared to the limit cycle parameter set for *Design 1A* and *Design 1B*: v_s = 0.18, β_s = 0.003, δ = 0.1, λ = 0, ε = 0.005. The resulting limit cycle is plotted in **Supplementary Figure 10**, and has a period of roughly 96 minutes.

The sensitivity analysis of the sustained oscillations is shown in **Supplementary Figure 11**. Some changes may be observed as compared to *Design 1B*. There is a significant increase in the absolute value of the control of most parameters (note the difference in y-axis values as compared to **Supplementary Figure 8**) but especially the control coefficients for v_x, v_s, β_x, δ, α_{xy} and α_{yz} increase in absolute value. This may be understood by the fact that λ had significant control in *Design 1A* and *Design 1B* and, in its absence, other parameters have to 'take over' this control. It appears that the δ parameter 'takes over' the control that was previously of λ, the other parameters that are changed in terms of their control. Therefore, there may be some complex systemic compensation for the absence of basal Sic1 degradation from the Clb/Cdk1/Sic1 ternary complex that is not dependent on the free Clb/Cdk1 concentrations.

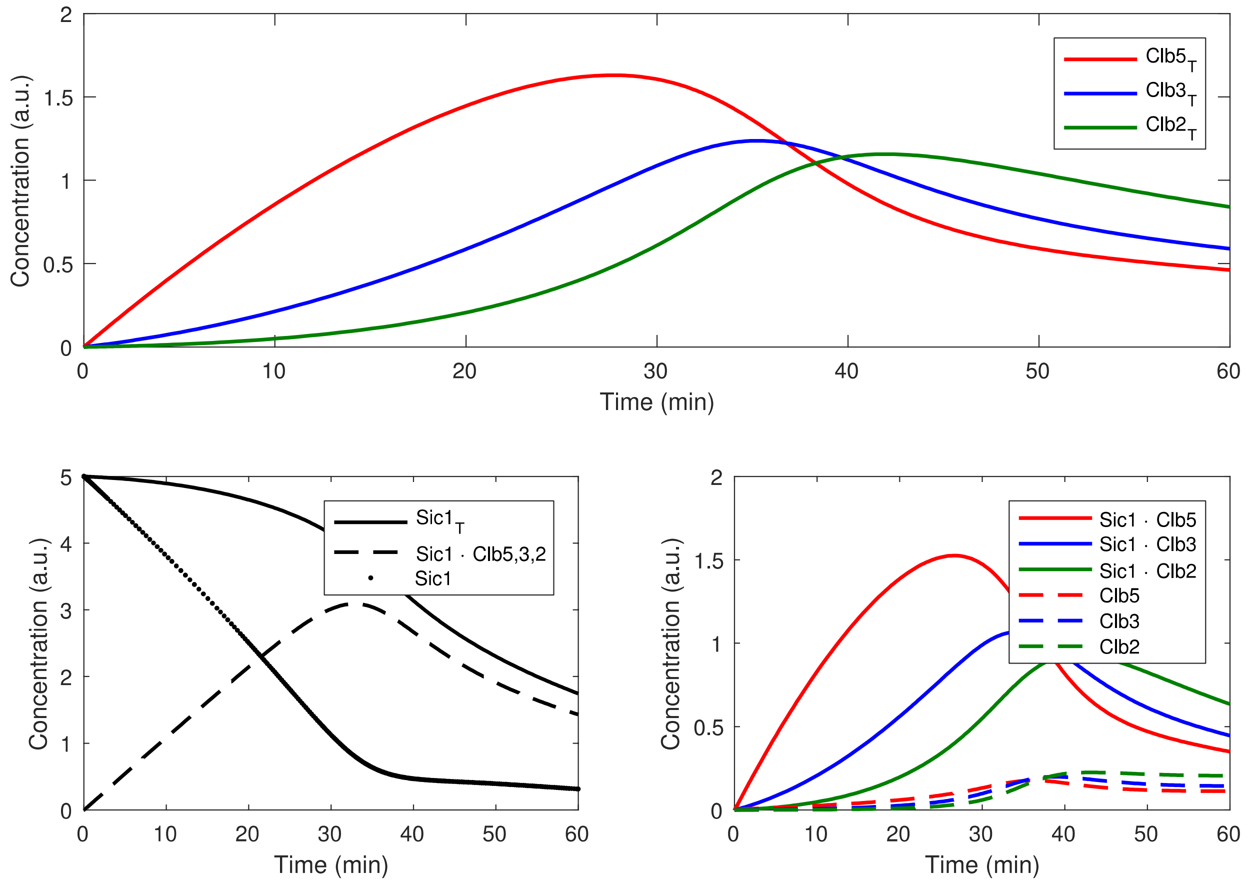

Supplementary Figure 9. Time courses for *Design 1C*. (Top) time courses for the total concentrations of the three Clb/Cdk1 complexes: Clb5/Cdk1, Clb3/Cdk1 and Clb2/Cdk1. (Bottom-left) time courses for total Sic1, Sic1 in complex with Clb/Cdk1 complexes, and Sic1. (Bottom-right) time courses for the binary (Clb/Cdk1) and ternary (Clb/Cdk1/Sic1) complexes.

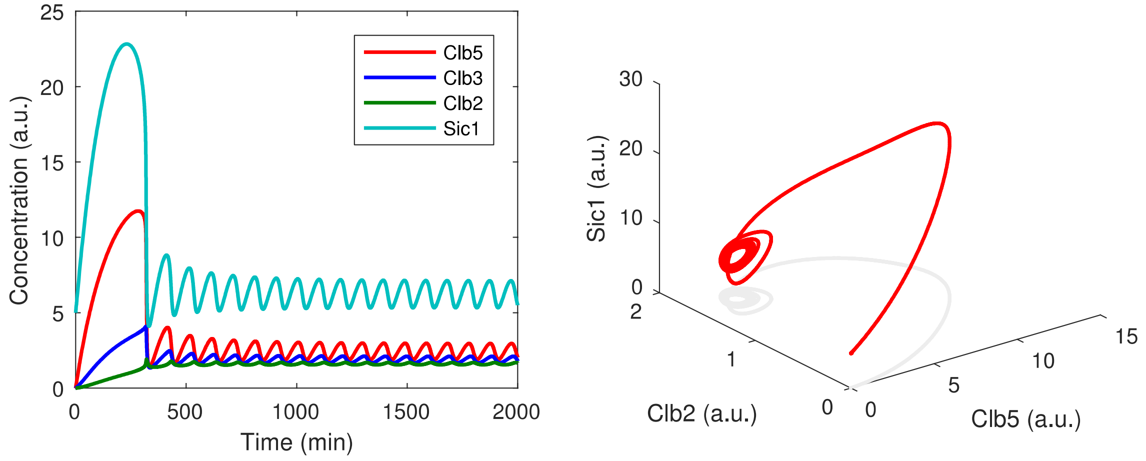

Supplementary Figure 10. Sustained oscillations in *Design 1C*. (Left) Limit cycle for the total concentrations of the four species. (Right) 3D view of the limit cycle in the Clb5-Clb2-Sic1 space.

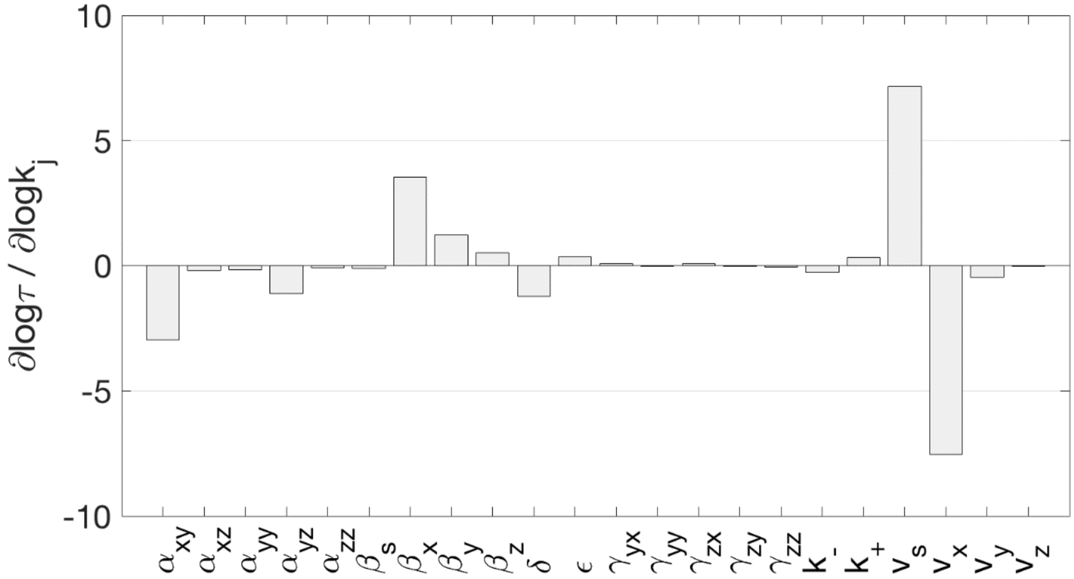

Supplementary Figure 11. Control coefficients of cell cycle times, obtained as logarithmic period derivatives for *Design 1C*. Of note, the control by λ has switched to parameter δ, and the control by v_x, v_s, β_x and α_{xy} has significant increased as compared to *Design 1B*.

1.5 *Design 2*: Including Clb3/Cdk1 and Clb3/Cdk1 salvaging

Design 2 includes the salvaging of Clb3/Cdk1 and Clb2/Cdk1 complexes when Sic1 is degraded from the Clb/Cdk1/Sic1 ternary complexes, as previously demonstrated for Clb5/Cdk1 [3] (**Supplementary Figure 1E)**.

$$\frac{d[x]}{dt} = v_x - \beta_x[x] - \gamma_{yx}[x][y] - \gamma_{zx}[x][z] - k^+[s][x] + k^-[s\cdot x]$$
$$+\delta([x]+[y]+[z])\,[s\cdot x]$$
$$\frac{d[y]}{dt} = v_y - \beta_y[y] + \alpha_{xy}[x] + \alpha_{yy}[y] - \gamma_{zy}[z][y] - \gamma_{yy}[y]^2 - k^+[s][y] + k^-[s\cdot y]$$
$$+\delta([x]+[y]+[z])\,[s\cdot y]$$
$$\frac{d[z]}{dt} = v_z - \beta_z[z] + \alpha_{zz}[z] - \gamma_{zz}[z]^2 + \alpha_{xz}[x] + \alpha_{yz}[y] - k^+[s][z] + k^-[s\cdot z]$$
$$+\delta([x]+[y]+[z])\,[s\cdot z]$$
$$\frac{d[s]}{dt} = v_s - \beta_s[s] - k^+([x]+[y]+[z])[s] + k^-\,([s\cdot x]+[s\cdot y]+[s\cdot z])$$
$$\frac{d[s\cdot x]}{dt} = k^+[s][x] - k^-[s\cdot x] - \delta([x]+[y]+[z])[s\cdot x] - \epsilon[s\cdot x]$$
$$\frac{d[s\cdot y]}{dt} = k^+[s][y] - k^-[s\cdot y] - \delta([x]+[y]+[z])[s\cdot y] - \epsilon[s\cdot y]$$
$$\frac{d[s\cdot z]}{dt} = k^+[s][z] - k^-[s\cdot z] - \delta([x]+[y]+[z])[s\cdot z] - \epsilon[s\cdot z]$$

Of note, positive δ terms in the ODEs for y and z have been added. The transient oscillations for the canonical parameter set for *Design 2* are shown in **Supplementary Figure 12**. The differences with the transient oscillations observed for *Design 1C* are negligible.

To obtain sustained oscillations in *Design 2*, a number of parameters had to be altered. Ultimately, autonomous oscillations were obtained varying the following parameters as compared to the limit cycle parameter set for *Design 1A* and *Design 1B*: $v_x = 0.09$, $v_s = 0.2$, $\beta_s = 0.005$, $\delta = 0.05$. The resulting limit cycle is plotted in **Supplementary Figure 13**, and has a period of roughly 182 minutes.

The sensitivity analysis of the sustained oscillations is shown in **Supplementary Figure 14**. The results are generally similar to those obtained for *Design 1C.*

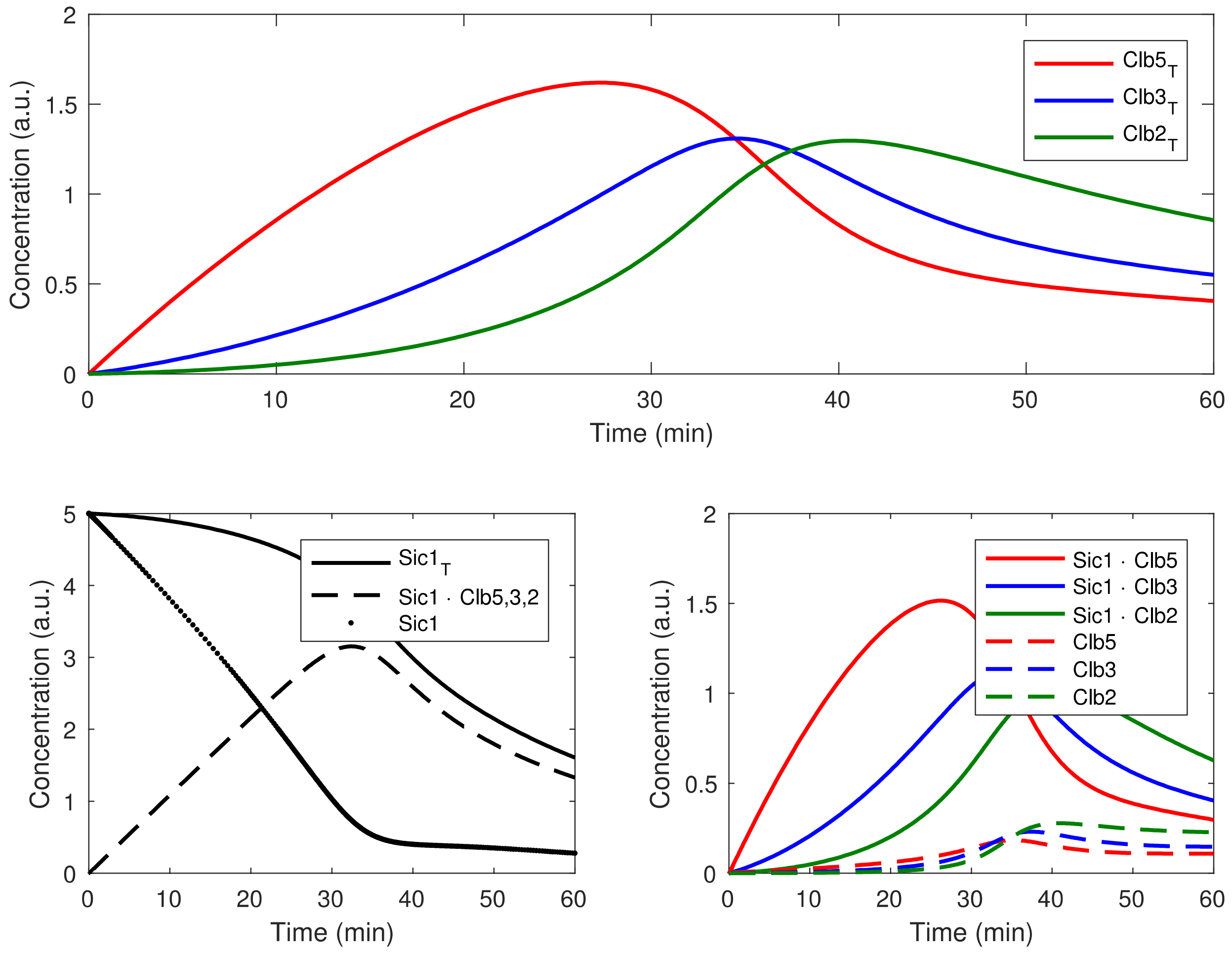

Supplementary Figure 12. Time courses for *Design 2*. (Top) time courses for the total concentrations of the three Clb/Cdk1 complexes: Clb5/Cdk1, Clb3/Cdk1 and Clb2/Cdk1. (Bottom-left) time courses for total Sic1, Sic1 in complex with Clb/Cdk1 complexes, and Sic1. (Bottom-right) time courses for the binary (Clb/Cdk1) and ternary (Clb/Cdk1/Sic1) complexes.

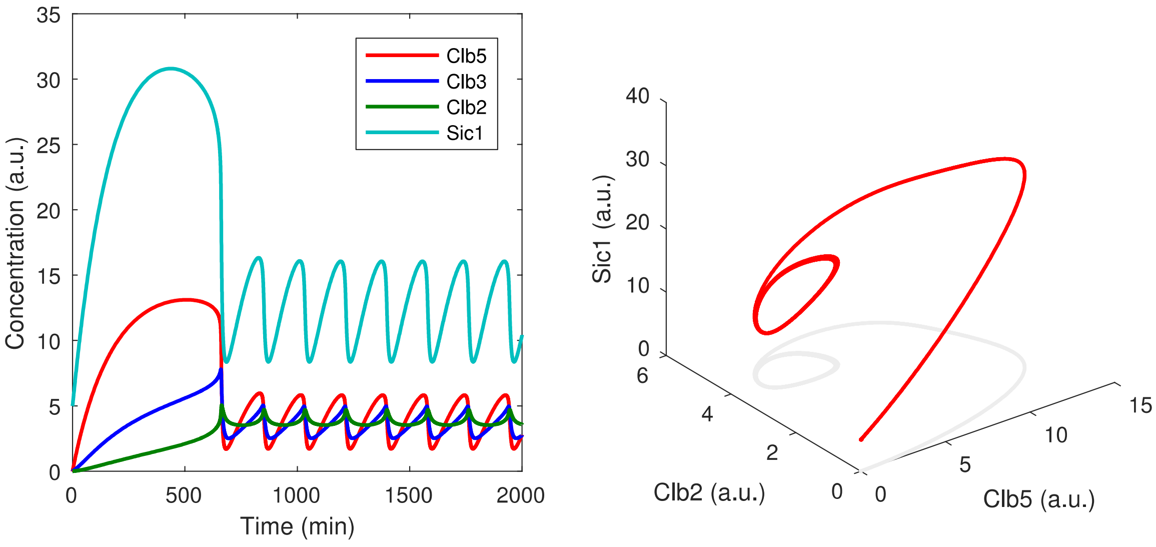

Supplementary Figure 13. Sustained oscillations in *Design 2*. (Left) Limit cycle for the total concentrations of the four species. (Right) 3D view of the limit cycle in the Clb5-Clb2-Sic1 space.

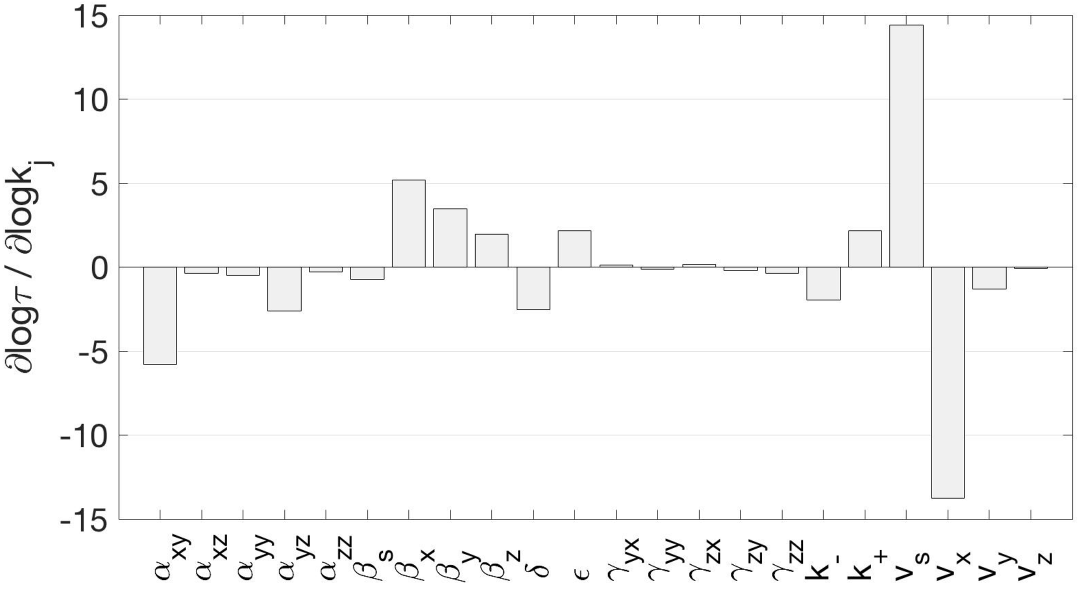

Supplementary Figure 14. Control coefficients of cell cycle times, obtained as logarithmic period derivatives for *Design 2*. Despite some numeric changes, the general direction and relative size of the derivatives are similar as compared to the analysis of *Design 1C*.

1.6 *Design 3*: The *quasi-steady-state* (QSS) model

Design 3 refers to a model including a *quasi-steady-state* approximation on the ternary complex formation between Clb/Cdk1 complexes and Sic1 (**Supplementary Figure 1E**). Although this assumption involves removal of one parameter, the mathematical description of the model is significantly altered.

1.6.1 Equations for total concentrations

Starting from *Design 2*, the system of equations was re-written in terms of the total concentrations of the four species considered. The total concentration of the Clb/Cdk1 complexes, i.e. for Clb5: $x_T = [x] + [s \cdot x]$ and analogous formulas y_T and z_T, and Sic1, i.e. $[s_T] = [s] + [s \cdot x] + [s \cdot y] + [s \cdot z]$, evolve according to the following:

$$\frac{d[x_T]}{dt} = v_x - \beta_x[x] - \gamma_{yx}[x][y] - \gamma_{zx}[x][z] - \epsilon[s \cdot x]$$

$$\frac{d[y_T]}{dt} = v_y - \beta_y[y] + \alpha_{xy}[x] + \alpha_{yy}[y] - \gamma_{zy}[z][y] - \gamma_{yy}[y]^2 - \epsilon[s \cdot y]$$

$$\frac{d[z_T]}{dt} = v_z - \beta_z[z] + \alpha_{zz}[z] - \gamma_{zz}[z]^2 + \alpha_{xz}[x] + \alpha_{yz}[y] - \epsilon[s \cdot z]$$

$$\frac{d[s_T]}{dt} = v_s - \beta_s[s] - \big(\epsilon + \delta([x] + [y] + [z])\big)([s \cdot x] + [s \cdot y] + [s \cdot z])$$

1.6.2 The *quasi-steady-state* approximation

We assume that Clb/Cdk1/Sic1 ternary complex formation and/or dissociation (k_+ and k_-) happen on a faster time-scale as compared to the other processes considered in the model. This implies that the ratio of active and ternary inactive complexes is at steady-state for a given Sic1 concentration. If we assume ternary complex degradation (δ, ε) is relatively small, the equilibrium condition implies the following:

$$\mathrm{k}_-[x \cdot s] = \mathrm{k}_+[x][s]$$

$$[x \cdot s] = K_A[x][s]$$

with $K_A = \frac{\mathrm{k}_+}{\mathrm{k}_-} = \frac{1}{K_D}$, where K_A and K_D are the association and dissociation constants, respectively. Similar equations hold for y and z. This implies the following:

$$\begin{aligned}[x_T] &= [x] + [x \cdot s] \\ &= [x](1 + K_A[s]) \\ &= \frac{1}{f} \cdot [x]\end{aligned}$$

where the variable $f([s])$ is introduced as follows:

$$f([s]) = \frac{1}{1 + [s]K_A}$$

Of note, *f* is a quantity that varies between 0 and 1. Since $[x] = f \cdot [x_T]$, f can be interpreted as the fraction of Clb/Cdk1 complex which is active, i.e. not in complex with Sic1, and that this fraction depends on the free Sic1 concentration $[s]$. Therefore, $f([s])$ is also time-dependent since $[s]$ is time-dependent. $K_D = 1/K_A$ represents the concentration $[s]$ for which $f([s]) = 1/2$. Of note, since $x = f \cdot [x_T]$ we have that $[x \cdot s] = (1 - f) \cdot [x_T]$.

1.6.3 The *quasi-steady-state* model

The quasi-steady-state assumption was incorporated in the expression of the total concentrations. The assumption can be considered for each Clb/Cdk1 species x, y or z

independently. The introduced variable $f([s])$ depends on K_A, but K_A depends only on k+ and k-, which are the same for x, y and z. Hence, $f([s])$ applies to all three Clb/Cdk1 complexes.

The equation for *f* has to be supplemented by an equation specifying the free Sic1 concentration $[s]$ at any moment in time. From the mass-balance for s we derive that:

$$
\begin{aligned}
[s_T] &= [s] + [s \cdot x] + [s \cdot y] + [s \cdot z] \\
&= [s]\big(1 + K_A([x] + [y] + [z])\big) \\
&= [s]\left(1 + K_A \frac{[x_T] + [y_T] + [z_T]}{1 + [s]K_A}\right)
\end{aligned}
$$

Multiplying out the fraction, we get a quadratic equation in $[s]$, as follows:

$$
[s_T](1 + [s]K_A) = [s]\big((1 + [s]K_A) + K_A([x_T] + [y_T] + [z_T])\big)
$$

$$
0 = [s]^2 + \left(\frac{1}{K_A} + [x_T] + [y_T] + [z_T] - [s_T]\right)[s] - \frac{[s_T]}{K_A}
$$

We find an expression for $[s]$ by solving quadratic equation and taking the positive root, as follows:

$$
[s] = -\frac{\frac{1}{K_A} + [x_T] + [y_T] + [z_T] - [s_T]}{2} + \sqrt{\left(\frac{\frac{1}{K_A} + [x_T] + [y_T] + [z_T] - [s_T]}{2}\right)^2 + \frac{[s_T]}{K_A}}
$$

Incorporating the *quasi-steady-state* assumption in the system for the total concentrations is achieved by writing down the equations for the total concentrations of the three Clb/Cdk1 species and Sic1 and replacing every occurrence of $[x]$ with $f[x_T]$ and $[s \cdot x]$ with $(1 - f)[x_T]$.

This is done similarly for y and z. The complete QSS model is defined by a system of four first-order non-linear ODEs for the total concentrations of the species together with the equations for f and s. For clarity and brevity of the equations, the expressions for $[s]$ and f are written separately, but they are to be simply substituted into the ODEs. In summary:

$$\frac{d[x_T]}{dt} = v_x - \beta_x f[x_T] - \gamma_{yx} f^2[x_T][y_T] - \gamma_{zx} f^2[x_T][z_T] - \epsilon(1-f)[x_T]$$

$$\frac{d[y_T]}{dt} = v_y - \beta_y f[y_T] + \alpha_{xy} f[x_T] + \alpha_{yy} f[y_T] - \gamma_{yy} f^2[y_T]^2 - \gamma_{zy} f^2[y_T][z_T] - \epsilon(1-f)[y_T]$$

$$\frac{d[z_T]}{dt} = v_z - \beta_z f[z_T] + \alpha_{xz} f[x_T] + \alpha_{yz} f[y_T] + \alpha_{zz} f[z_T] - \gamma_{zz} f^2[z_T]^2 - \epsilon(1-f)[z_T]$$

$$\frac{d[s_T]}{dt} = v_s - \beta_s[s] - \big(\epsilon + \delta\, f\,([x_T] + [y_T] + [z_T])\big)(1-f)([x_T] + [y_T] + [z_T])$$

The concentrations of the binary (Clb/Cdk1) and ternary (Clb/Cdk1/Sic1) complexes can be deduced by multiplying the total concentrations by f and $1-f$, respectively.

1.6.4 Numerical validation of the *quasi-steady-state* approximation

As a preliminary theoretical validation of our modeling approach, we explore the discrepancy between *Design 2* equations and the *quasi-steady-state* assumption in *Design 3*. If this assumption is satisfied, by high parameter values for Clb/Cdk1/Sic1 ternary complex formation and/or dissociation in *Design 2*, we would expect similar results between the two models. Conversely, if this assumption breaks down and these parameters become small, the two models should disagree in their behavior.

To test this assumption, we consider the canonical parameter set and simulate both models. We map the K_A parameter in the QSS model to the k_+ and k_- parameters in the standard model through the rule $k_+ = K_A\, k_-$. In the canonical parameter set K_A = 10. While keeping K_A constant

(i.e. equal to 10), we can vary either k_+ or k_- and keep track of the model behavior. In **Supplementary Figure 15**, the discrepancy between the behavior of both models is shown, measured in terms of the Average Relative Difference, i.e. $ARD = \frac{1}{\#species \cdot \#time\ steps} \sum_x \sum_t \left| \frac{x(t) - x^*(t)}{x(t)} \right|$ for the canonical parameter set but at different values for k_+ and k_- at constant ratio K_A, where x indicates the total concentrations of each of the four species (Sic1, Clb5, Clb3, Clb2) at time t in *Design 2*, and x^* indicates the same in *Design 3*. t indicates the time points at which the ODE solver returns the solution. As shown in **Supplementary Figure 15**, for high K_A values the QSS model numerically returns the same behavior as *Design 2*. Therefore, we expect all results to be roughly similar for both *Design 2* and *Design 3*.

Transient oscillations for *Design 3* are plotted in **Supplementary Figure 16**. To obtain sustained oscillations in *Design 3*, we used the same parameter set as for *Design 2*. The resulting limit cycle is plotted in **Supplementary Figure 17**, and has a period of roughly 226 minutes. The sensitivity analysis of the sustained oscillations is shown in **Supplementary Figure 18**. The results are generally similar to those obtained for *Design 2* and *Design 1C*.

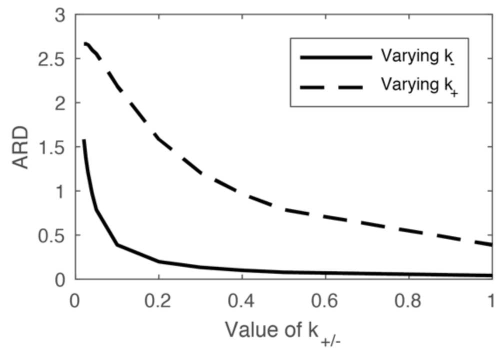

Supplementary Figure 15. Analysis of the validity of the QSS assumption in the parameter space. The sum of squared errors (SSE) is measured between the model with (*Design 3*) and without (*Design 2*) the assumption. The k_- parameter is varied along the continuous line, whereas the k_+ parameter is varied along the dashed line, always for constant K_A. The two models exhibit a similar behavior for higher values of k_+ and k_-.

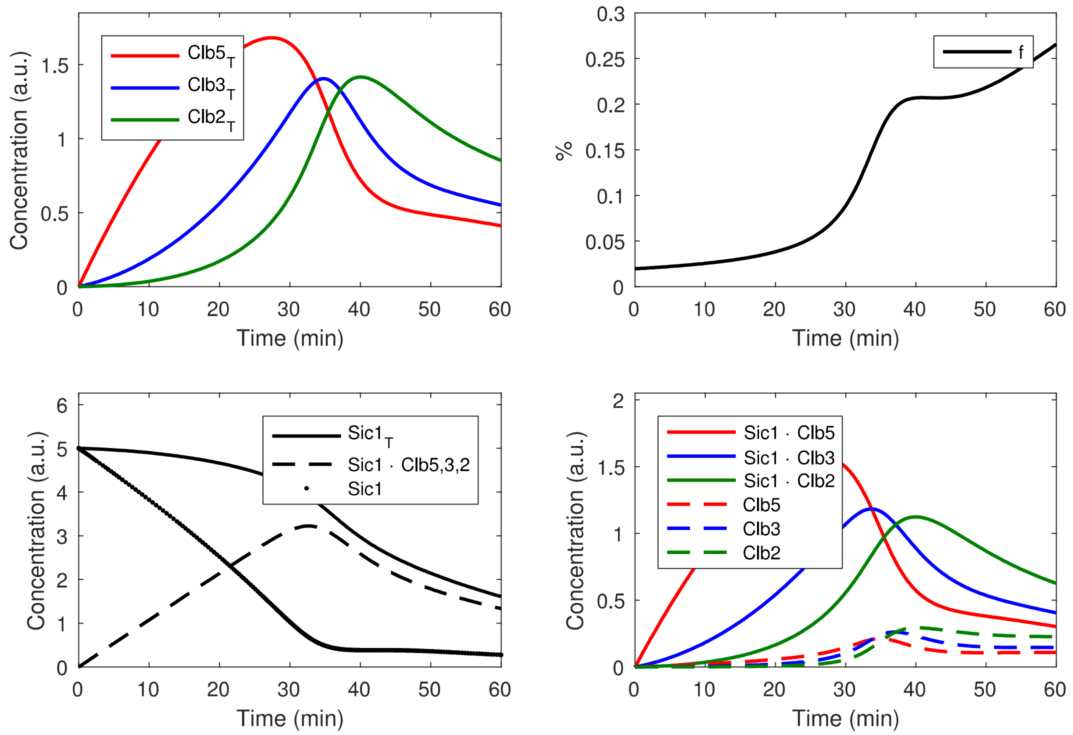

Supplementary Figure 16. Time courses for *Design 3*. (Top-left) time courses for the total concentrations of the three Clb/Cdk1 complexes: Clb5/Cdk1, Clb3/Cdk1 and Clb2/Cdk1. (Top-tight) Plot of the function f over time. (Bottom-left) time courses for total Sic1, Sic1 in complex with Clb/Cdk1 complexes, and Sic1. (Bottom-right) time courses for the binary (Clb/Cdk1) and ternary (Clb/Cdk1/Sic1) complexes.

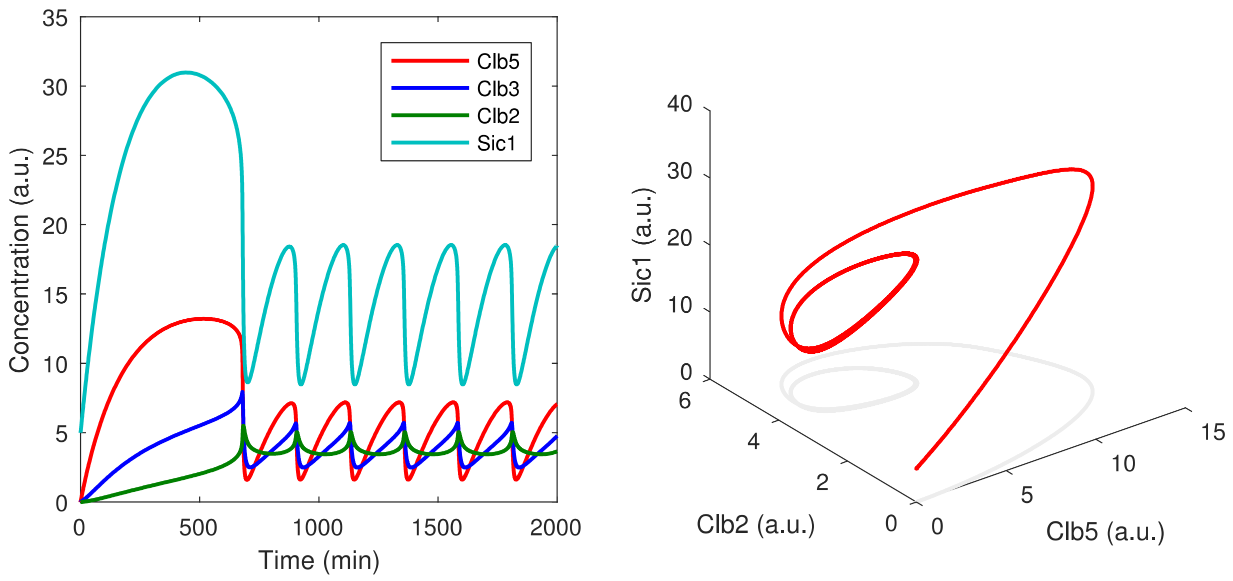

Supplementary Figure 17. Sustained oscillations in *Design 3.* (Left) Limit cycle for the total concentrations of the four species. (Right) 3D view of the limit cycle in the Clb5-Clb2-Sic1 space.

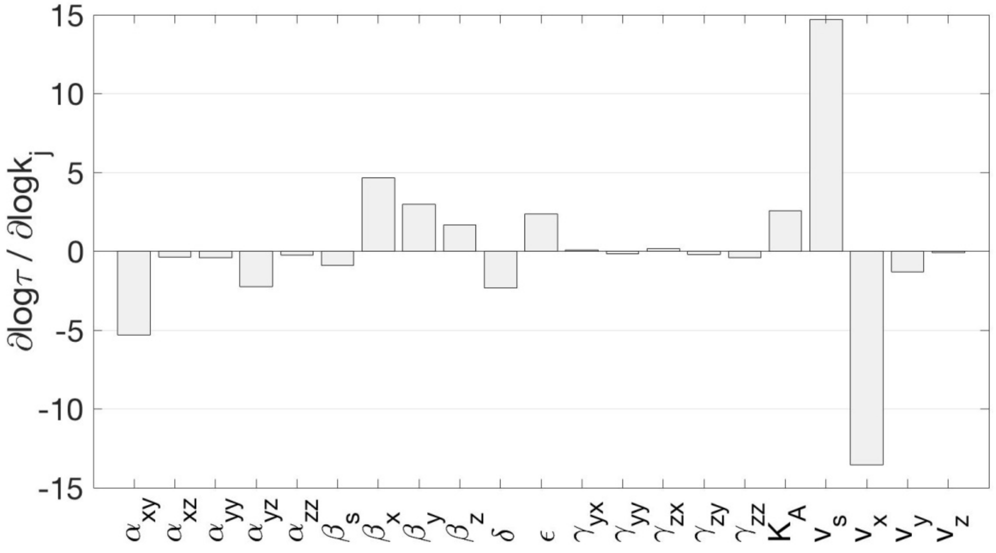

Supplementary Figure 18. Control coefficients of cell cycle times, obtained as logarithmic period derivatives for *Design 3.* Of note, despite some numeric changes, the general direction and relative size of the derivatives are similar as compared to the analysis of *Design 2*.

2 Summary of autonomous oscillations for designs *1A–3*

All five models corresponding to the designs illustrated in **Supplementary Figure 1** are able to yield sustained oscillations in the form of limit cycles. For each design, initial parameter sets resulting in limit cycles were identified through a manual adjustment of one or several model parameters through the slider functionality in COPASI [4], starting from the canonical parameter set [1] (**Supplementary Table 1**).

We quantified the control on the period of the model parameters (**Supplementary Figures 5, 8, 11, 14** and **18**). The analysis of our models indicates that this control is shared among several parameters, as it has been observed in other biochemical oscillatory systems for which the function of the oscillation is unclear [15]. This is the first time that a distributed control is found for an oscillation as functional as an autonomous cell cycle. The results recover the fact that the sum of the control coefficients on the period of oscillations for all the parameters with dimension

1/time must equal –1 [8]. Control coefficients of (–)1 indicate that the controlled property is (inversely) proportional to the controlling parameter. Among the parameters that exerted a more considerable control in Design 3 (i.e. an absolute value of the control coefficient > 2), some increased (v_s, β_x, β_y, K_A, ε) and some decreased (negative control coefficient; v_x, α_{xy}, α_{yz}, δ) the period of oscillations. The latter correspond to the parameters that activate cell cycle progression by decreasing the period. Indeed, they stimulate the formation of Clb5/Cdk1, Clb3/Cdk1, Clb2/Cdk1 and the degradation of Sic1, respectively. Conversely, activation of the former should inhibit cell cycle progression, by stimulating Sic1 formation, dissociation of Clb5/Cdk1 and Clb3/Cdk1 and by favoring the formation and degradation of the ternary Clb/Cdk1/Sic1 complex. The control that these parameters exhibit is conserved between designs *1C*, *2* and *3* (**Supplementary Figures 11, 14** and **18**, respectively). Strikingly, the Clb3 PFL (associated to the parameter α_{yy}), the Clb3 NFL (γ_{yy}) – as well as the Clb2 PFL (α_{zz}) and the Clb2 NFL (γ_{zz}) – and the four known Clb-regulated inhibitory regulations mediated by both the Clb/Cdk1 complexes and Anaphase-Promoting Complex, APC (see Section 1.1) exerted almost no control over the period of oscillations.

3 *Designs 4* through *9*: Inhibitory regulations that may boost oscillatory potential

Below we briefly highlight the changes in the equations for *Design 4* through *Design 9* as compared to *Design 3*.

3.1 *Design 4*

Design 4 entails inhibition of Clb5 synthesis by Clb2/Cdk1. The SBF transcription factor, formed by Swi4 and Swi6, promotes transcription of the G1 phase cyclin genes *CLN1* and *CLN2*; the MBF transcription factor, formed by Mbp1 and Swi6, promotes transcription of the S phase cyclin genes *CLB5* and *CLB6*. Genetic evidence indicates that *CLB2* and *SWI6* are functionally

related [16], and Clb2 has been shown to interact physically with Swi4, thus repressing transcription of the G1 cyclins [17]. Inhibition of the G1 cyclins translates to an effective inhibition of the Clb5/Cdk1 activity, due to the lack of the PFL between Cln2/Cdk1 and SBF/MBF [18] and to the lifted inhibition of Sic1 by Cln1,2/Cdk1 [19].

The altered ODE for Clb5 (x) is now written as follows:

$$\frac{d[x_T]}{dt} = v_x\left(\frac{1}{1+\frac{z_T}{K_{zx}}}\right) - \beta_x f[x_T] - \gamma_{yx} f^2[x_T][y_T] - \gamma_{zx} f^2[x_T][z_T] - \epsilon(1-f)[x_T]$$

This structural change in the equations with the inhibitory term is representative for all subsequent designs. Of note, v_x, which used to represent the synthesis of Clb5, is now the V_{max} of the synthesis, which is attained when Clb2 is not present.

3.2 *Design 5*

Design 5 entails inhibition of Sic1 synthesis by Clb2/Cdk1 through the *SWI5* transcription factor. During the G2 phase, Cdk1 phosphorylates specific serine residues of Swi5 near the NLS (Nuclear Localization Sequence) at its C-terminal, in order to keep Swi5 sequestered in the cytoplasm [20], effectively inhibiting *SIC1* transcription. The Cdk-dependent phosphorylation reaction may be reversed by the phosphatase Cdc14 [21], which thus contributes to *SIC1* transcription. *Design 5* describes the inhibition of *SIC1* transcription mediated by the Clb/Cdk1 activity, reflecting the likely scenario where the most abundant Cdk1 activity is due to Clb2/Cdk1.

The altered ODE for Sic1 (s) is now written as follows:

$$\frac{d[s_T]}{dt} = v_s(\frac{1}{1+\frac{z_T}{K_{zs}}}) - \beta_s[s] - \left(\epsilon + \delta f\ ([x_T] + [y_T] + [z_T])\right)(1-f)([x_T] + [y_T] + [z_T])$$

3.3 *Design 6*

Design 6 entails inhibition of Sic1 synthesis by Clb2/Cdk1, Clb3/Cdk1 and Clb5/Cdk1 through the *SWI5* transcription factor. *Design 6* describes the same mechanism detailed for *Design 5* but mediated by the three Clb/Cdk1 complexes: Clb2/Cdk1, Clb3/Cdk1 and Clb5/Cdk1.

The altered ODE for Sic1 (s) is now written as follows:

$$\frac{d[s_T]}{dt} = v_s(\frac{1}{1+\frac{x_T + y_T + z_T}{K_{cs}}}) - \beta_s[s] - \left(\epsilon + \delta f\ ([x_T] + [y_T] + [z_T])\right)(1-f)([x_T] + [y_T] + [z_T])$$

3.4 *Design 7*

Design 7 entails inhibition of Clb2 and Clb3 syntheses by Sic1. A recent study that integrated experimentation and computer modeling showed that Sic1 oscillations rescue viability of cells with low levels of mitotic Clb cyclins [22]. However, the molecular mechanism(s) at the basis of this observation at the moment remains obscure. Here we propose that inhibition of Clb2 and Clb3 synthesis by Sic1 may rationalize this observation; both *CLB2* and *CLB3* genes appear to be regulated by a similar transcriptional mechanism [2], thus we have incorporated for both the same Sic1-mediated inhibitory regulation.

The altered ODEs for Clb3/Cdk1 (y) and Clb2/Cdk1 (z) are now written as follows:

$$\frac{d[y_T]}{dt} = v_y(\frac{1}{1+\frac{s_T}{K_{syz}}}) - \beta_y f[y_T] + \alpha_{xy} f[x_T] + \alpha_{yy} f[y_T] - \gamma_{yy} f^2[y_T]^2 - \gamma_{zy} f^2[y_T][z_T] - \epsilon(1-f)[y_T]$$

$$\frac{d[z_T]}{dt} = v_z(\frac{1}{1+\frac{s_T}{K_{syz}}}) - \beta_z f[z_T] + \alpha_{xz} f[x_T] + \alpha_{yz} f[y_T] + \alpha_{zz} f[z_T] - \gamma_{zz} f^2[z]^2 - \epsilon(1 - f)[z_T]$$

Of note, *Design 7* is slightly different from *Design 4*, *Design 5* and *Design 6*, as the inhibitory term is assumed to affect basal synthesis while there are other synthesis terms in the equations that are not affected.

3.5 *Design 8*

Design 8 entails inhibition of Clb2, Clb3 and Clb5 syntheses by Sic1. The altered ODEs for Clb5/Cdk1, Clb3/Cdk1 and Clb2/Cdk1 are now written as follows:

$$\frac{d[x_T]}{dt} = v_x(\frac{1}{1+\frac{s_T}{K_{sxyz}}}) - \beta_x f[x_T] - \gamma_{yx} f^2[x_T][y_T] - \gamma_{zx} f^2[x_T][z_T] - \epsilon(1-f)[x_T]$$

$$\frac{d[y_T]}{dt} = v_y(\frac{1}{1+\frac{s_T}{K_{sxyz}}}) - \beta_y f[y_T] + \alpha_{xy} f[x_T] + \alpha_{yy} f[y_T] - \gamma_{yy} f^2[y_T]^2 - \gamma_{zy} f^2[y_T][z_T] - \epsilon(1-f)[y_T]$$

$$\frac{d[z_T]}{dt} = v_z(\frac{1}{1+\frac{s_T}{K_{sxyz}}}) - \beta_z f[z_T] + \alpha_{xz} f[x_T] + \alpha_{yz} f[y_T] + \alpha_{zz} f[z_T] - \gamma_{zz} f^2[z]^2 - \epsilon(1 - f)[z_T]$$

3.6 *Design 9*

Design 9 entails inhibition of Sic1 synthesis by Sic1 through the *SWI5* transcription factor. This regulation does not have any experimental support; however, we aimed to test all possible Sic1-dependent negative regulations.

The altered ODE for Sic1 is now written as follows:

$$\frac{d[s_T]}{dt} = v_s(\frac{1}{1+\frac{s_T}{K_{ss}}}) - \beta_s[s] - \left(\epsilon + \delta\, f\ ([x_T] + [y_T] + [z_T])\right)(1-f)([x_T] + [y_T] + [z_T])$$

4 System Design Space (SDS) methodology

In this work we make an extensive use of the System Design Space (SDS) methodology developed by Savageau and collaborators [23–26] and the associated Python toolbox [26]. The cited papers should be seen as required reading to reproduce the analyses presented in our work. We especially point the reader to the Supplementary Information accompanying [26], which contains an excellent introduction to the terminology with clear examples.

The SDS methodology has been proposed to overcome the analytical difficulty to find limit cycles. In 1900, David Hilbert posed his 16th problem, which partially concerns the finding of the number of limit cycles of a polynomial differential equation in the plane [28]. The Bendixson-Dulac theorem and the Poincaré-Bendixson theorem predict the absence or existence, respectively, of limit cycles of two-dimensional nonlinear dynamical systems. However, these theorems do not help to actually to find the parameter sets that generate limit cycles.

The SDS methodology has grown out of the Biochemical Systems Theory (BST), in which every process (generalized mass-action reaction [29]) is formulated in a simplified way as a product of power-law functions [30]. A trade-off exists between (i) the accuracy by which system's complexity is modeled and (ii) the computational cost to analyze a model and the complexity of its results. The SDS methodology starts from a system of ordinary differential equations (ODEs), described by using generalized mass-action (GMA) kinetics [30]. First, the set of all combinatorically possible combinations of single dominant positive and negative terms in each ODE is generated. The reduction to dominant processes transforms the ODE system into an S-system. S-systems can capture saturable and synergistic (thus the capital S) properties of a biochemical system [31], and is then referred to as a *phenotype* that can be used

to approximate the full GMA model [23]. In an S-system, for a particular phenotype, every ODE consists of a single dominant positive term and a single dominant negative term. For a given set of parameters and concentrations there exists a single dominant positive term, i.e. largest, and a single dominant negative term in each differential equation. The dominance of certain positive and negative terms gives rise to *dominance* conditions, i.e. inequalities stating that the dominant positive (negative) term is larger than the other positive terms in a specific ODE. Altogether, the dominance conditions form a set of inequalities that are either inconsistent, i.e. there is no set of parameters and concentrations that satisfies them all, or consistent. When reducing the mathematical description of the phenotypes to these dominant processes, a biochemical system becomes mathematically tractable, and a consistent set of dominance conditions defines boundaries within the parameter space and (reaction) state space within which the dominance conditions, and therefore the phenotype, are valid [26,31]. In this way, a phenotype may be viewed as a bounded area within the parameter and state space. Subsequently, by transforming the equations to logarithmic coordinates, the S-system becomes linear, and an analytical solution for the steady states may be obtained [23]. In addition to this analytical solution, properties such as the stability of steady states may be determined. This is particularly relevant for the identification of limit cycles, since Hopf bifurcations that give rise to limit cycles occur when a pair of complex conjugate eigenvalues crosses the imaginary axis. Consequently, a fixed point (steady state in the mathematical term) with two complex conjugate eigenvalues with positive real parts is a necessary condition for the occurrence of limit cycles after undergoing a Hopf bifurcation. Therefore, by using the SDS methodology, phenotypes with unstable steady states that have two complex conjugate eigenvalues with positive real parts may suggest bounded areas of the parameter space that might generate limit cycles, as highlighted previously [26]. This procedure greatly reduces the area of the parameter space to be sampled, and allows for the exploration of relatively small areas that might otherwise be overlooked.

4.1 Application of the SDS methodology and GMA casting for *Design 7*

We start here by deriving the 11 different model designs that we considered in our work. For designs *1A*, *1B*, *1C*, *2* and *3*, we show (i) transient oscillations using a canonical parameter set (see Section 1.3, **Supplementary Table 1**), an initial parameter set yielding limit cycles, and (ii) a sensitivity analysis for all parameters on the period of oscillations. *Design 4* through *Design 9* develop on *Design 3*, and for these we highlight the changes that occur in the equations.

To implement any design that we considered in our work such that the models work with the System Design Space Toolbox, these need to be translated into their GMA (Generalized Mass Action) form. In the following, we use *Design 7* to illustrate the translation from the Ordinary Differential Equations (ODEs) into the Generalized Mass-Action (GMA) form. In the GMA form, all equations must consist of sums of products of parameters and concentrations that may be raised to a power. For *Design 7*, the ODEs are written as follows:

$$\frac{d[x_T]}{dt} = v_x - \beta_x f[x_T] - \gamma_{yx} f^2[x_T][y_T] - \gamma_{zx} f^2[x_T][z_T] - \epsilon(1-f)[x_T]$$

$$\frac{d[y_T]}{dt} = v_y\left(\frac{1}{1+\frac{s_T}{K_{syz}}}\right) - \beta_y f[y_T] + \alpha_{xy} f[x_T] + \alpha_{yy} f[y_T] - \gamma_{yy} f^2[y_T]^2 - \gamma_{zy} f^2[y_T][z_T] - \epsilon(1-f)[y_T]$$

$$\frac{d[z_T]}{dt} = v_z\left(\frac{1}{1+\frac{s_T}{K_{syz}}}\right) - \beta_z f[z_T] + \alpha_{xz} f[x_T] + \alpha_{yz} f[y_T] + \alpha_{zz} f[z_T] - \gamma_{zz} f^2[z]^2 - \epsilon(1 - f)[z_T]$$

$$\frac{d[s_T]}{dt} = v_s - \beta_s[s] - \big(\epsilon + \delta\, f\, ([x_T]+[y_T]+[z_T])\big)(1-f)([x_T]+[y_T]+[z_T])$$

$$f([s]) = \frac{1}{1+[s]K_A}$$

$$[s] = -\frac{\frac{1}{K_A} + [x_T] + [y_T] + [z_T] - [s_T]}{2} + \sqrt{\left(\frac{\frac{1}{K_A} + [x_T] + [y_T] + [z_T] - [s_T]}{2}\right)^2 + \frac{[s_T]}{K_A}}$$

For the GMA form, we need to get rid of any fractions, i.e. in the equations for *f* and *s*, and we need to expand brackets, i.e. in the equations for *x*, *y*, *z* and *s*. For the $(1-f)$ terms, we introduce a new variable f_{inv} ; for the $[x_T]+[y_T]+[z_T]$ terms, we introduce a new variable Clb_T. In the equations for *f* and s_{free}, we introduce several auxiliary variables to satisfy the GMA form. Ultimately, the GMA form is written as follows:

$$\frac{d[x_T]}{dt} = v_x - \beta_x f[x_T] - \gamma_{yx} f^2 [x_T][y_T] - \gamma_{zx} f^2 [x_T][z_T] - \epsilon f_{inv}[x_T]$$

$$\frac{d[y_T]}{dt} = v_y \, \mathrm{aux}_3^{-1} - \beta_y f[y_T] + \alpha_{xy} f[x_T] + \alpha_{yy} f[y_T] - \gamma_{yy} f^2 [y_T]^2 - \gamma_{zy} f^2 [y_T][z_T] - \epsilon f_{inv}[y_T]$$

$$\frac{d[z_T]}{dt} = v_z \, \mathrm{aux}_3^{-1} - \beta_z f[z_T] + \alpha_{xz} f[x_T] + \alpha_{yz} f[y_T] + \alpha_{zz} f[z_T] - \gamma_{zz} f^2 [z_T]^2 - \epsilon f_{inv}[z_T]$$

$$\frac{d[s_T]}{dt} = v_s - \beta_s [s_{free}] - (\epsilon + \delta \, f \, clb_T) f_{inv} \, clb_T$$

$$f([s]) = \frac{1}{f_{denom}}$$

$$f_{denom} = 1 + [s_T] K_A$$

$$f_{inv} = 1 - f$$

$$clb_T = [x_T] + [y_T] + [z_T]$$

$$s_{free} = -\frac{1}{2} K_A^{-1} + \frac{1}{2}[s_T] - \frac{1}{2} clb_T + aux_1^{\frac{1}{2}}$$

$$aux_1 = [s_T] \, K_A^{-1} - \frac{1}{4} aux_2^2$$

$$aux_2 = K_A^{-1} - [s] + clb_T$$

$$aux_3 = 1 + [s_T] K_{syz}^{-1}$$

In Python code that is readable by the System Design Space Toolbox, the equations are written as follows:

```
Eq = [
'x. = v_x - b_x * f * x - g_yx * f^2 * x * y - g_zx * f^2 * x * z - e * f_inv * x',
'y. = v_y * aux3^(-1.0) - b_y * f * y + a_xy * f * x + a_yy * f * y - g_yy * f^2 * y^2 - g_zy * f^2 * z * y - e
* f_inv * y',
'z. = v_z * aux3^(-1.0) - b_z * f * z + a_xz * f * x + a_yz * f * y + a_zz * f * z - g_zz * f^2 * z^2 - e *
f_inv * z',
's. = v_s - b_s * s_free - e * f_inv * clbT - d * f_inv * clbT * f * clbT',
's_free =  -(1/2.0)*K_A^(-1.0) + (1/2.0)*s - (1/2.0)*clbT + aux1^(1/2.0)',
'f_inv = 1 - f',
'f = f_denom^(-1.0)',
'f_denom = (1+s_free*K_A)',
'aux1 = s*K_A^(-1.0) + (1/4.0)*aux2^2.0',
'aux2 = K_A^(-1.0) - s + clbT',
'clbT = x+y+z',
'aux3 = 1 + s*K_syz^(-1.0)'
]
```

5 Experimental evidence of the interactions and regulations in designs *1–9*

The known experimental evidence for the regulations across all designs used in this work are listed in **Supplementary Table 2**.

Supplementary Table 2. Overview of the experimental evidence for the interactions and regulations in the model designs considered in this work, along with the associated parameter symbol and relevant notes. The symbol -> indicates activations, -| indicates inhibitions, and <-> indicates reversible complex formation. For clarity, the relevant references are grouped based on the described interaction/regulation.

**Interaction**	**Symbol**	**References**	**Notes**
Sic1 <-> Clb5,3,2	$k^{+/-}$	[1]	Fast complex formation is incorporated in Design 3-9 (Mart Loog, personal communication)
Clb5 -> Clb3	α_{xy}	[2], [32–35]	
Clb5 -> Clb2	α_{xz}	[32–35], [2,36,37]	
Clb3 -> Clb3	α_{yy}	[2], [2*]	
Clb3 -> Clb2	α_{yz}	[2,36,37], [2*]	
Clb2 -> Clb2	α_{zz}	[32–35], [2,36,37]	
Clb2 -| Clb2	γ_{zy}	[13], [38]	Absent in Design 1A
Clb2 -| Clb3	γ_{zy}	[1], [38]	Absent in Design 1A. We hypothesize that Clb3 is targeted for degradation by APC-Cdc20
Clb2 -| Clb5	γ_{zx}	[37], [39,40]	Absent in Design 1
Clb3 -| Clb3	γ_{yy}	[1]	Absent in Design 1A. We hypothesize that Clb3 may activate APC-Cdc20. We hypothesize that Clb3 is targeted for degradation by APC-Cdc20
Clb3 -| Clb5	γ_{yx}	[1], [39,40]	Absent in Design 1A. We hypothesize that Clb3 may activate APC-Cdc20
Clb5,3,2 -> Sic1-Clb5,3,2	δ	[18,41]	
Clb2 -| Clb5	K_{zx}	[16]	Present only in Design 4
Clb2 -| Sic1	K_{zs}	[19]	Present only in Design 5-6
Clb3,5 -| Sic1	K_{cs}	–	Present only in Design 6. We hypothesize that Clb3/Cdk1 and Clb5/Cdk1 may inhibit *SIC1* transcription
Sic1 -| Clb2,3	K_{syz}	[21]	Present only in Design 7-8. We hypothesize that Sic1 may inhibit *CLB2 and CLB3* transcription
Sic1 -| Clb5	K_{sxyz}	[21]	Present only in Design 8. We hypothesize that Sic1 may inhibit *CLB5* transcription
Sic1 -| Sic1	K_{ss}	–	Present only in Design 9. We hypothesize that Sic1 may inhibit *SIC1* transcription

[1]: Sic1 interacts and co-exists in time with Clb5/Cdk1, Clb3/Cdk1 and Clb2/Cdk1.
[2]: Fkh2 regulates *CLB3* expression.
[2*]: Clb3,4/Cdk1 play a role in Fkh2 phosphorylation.
[32–35]: Clb5/Cdk1 and Clb2/Cdk1 interact with, and phosphorylate, Fkh2 to control Clb1,2 accumulation.
[2, 36,37] *CLB1,2* transcription is regulated by Fkh2 during the G2/M phase.
[38] Phosphorylation of Cdc20 by Clb2/Cdk1 activates APC-Cdc20.
[13] APC-Cdc20 degrades mitotic cyclins.
[39,40] APC-Cdc20 targets Clb5 for degradation.
[18,41] Clbs/Cdk1 phosphorylate Sic1, resulting in the recognition of Sic1 by the protein degradation machinery.
[16] Clb2/Cdk1 interacts with Swi4 and represses transcription of G1 cyclins. This regulation translates to an effective inhibition of Clb5/Cdk1, due to the lack of the PFL between Cln2/Cdk1 and SBF/MBF [17] and to the lifted inhibition of Sic1 by Cln1,2/Cdk1 [18,40].
[19] Inhibition of *SIC1* transcription is mediated by Clb2/Cdk1.
[21] Hypothetical interaction to rationalize the observation that Sic1 oscillations rescue viability of cells with low levels of mitotic cyclins (Clb2).

6 Parameter correlation analysis in limit cycles

Within the set of identified limit cycles for each model design, we analyzed whether there were correlations present between the parameters as measured by the Pearson correlation coefficient. **Supplementary Figures 19–25** summarize the correlation coefficients as heatmaps between all model parameters across the seven model designs that returned limit cycles. In **Supplementary Table 3**, the set of combinations of two parameters that were highly correlated (absolute value of correlation coefficient ≥ 0.5) in three or more of the designs are summarized.

Supplementary Figures 19–25. Heatmap of Pearson correlation coefficients between all model parameters for designs *3–9* across all identified limit cycles.

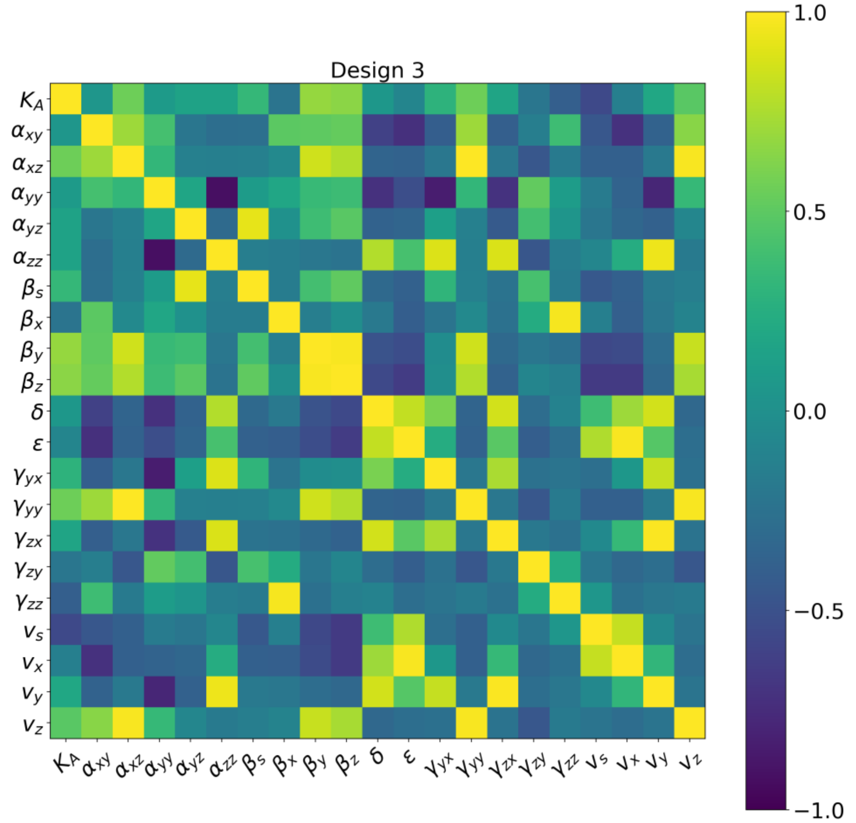
Design 3
K_A, α_{xy}, α_{xz}, α_{yy}, α_{yz}, α_{zz}, β_s, β_x, β_y, β_z, δ, ε, γ_{yx}, γ_{yy}, γ_{zx}, γ_{zy}, γ_{zz}, v_s, v_x, v_y, v_z
1.0
0.5
0.0
−0.5
−1.0

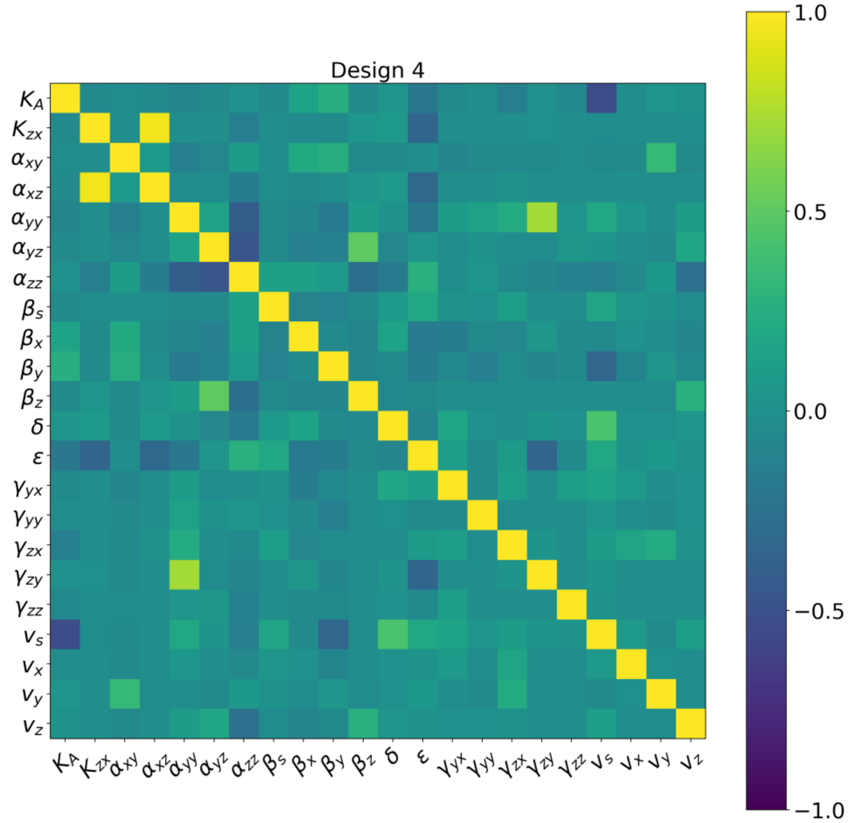
Design 4
K_A, K_{zx}, α_{xy}, α_{xz}, α_{yy}, α_{yz}, α_{zz}, β_s, β_x, β_y, β_z, δ, ε, γ_{yx}, γ_{yy}, γ_{zx}, γ_{zy}, γ_{zz}, v_s, v_x, v_y, v_z
1.0
0.5
0.0
−0.5
−1.0

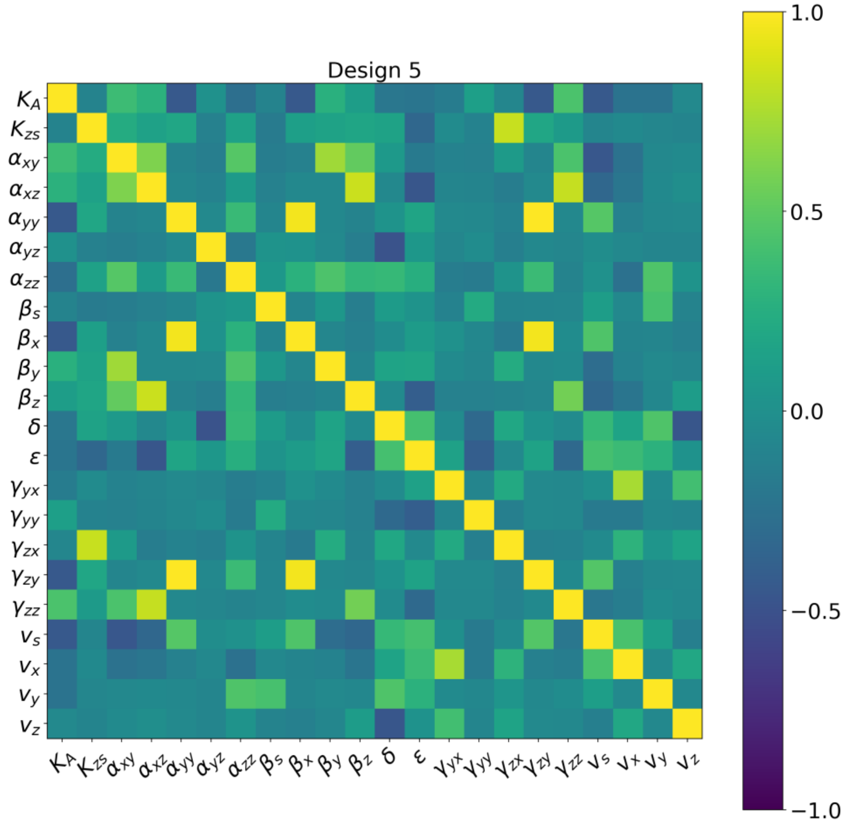
Design 5
KA
Kzs
αxy
αxz
αyy
αyz
αzz
βs
βx
βy
βz
δ
ε
γyx
γyy
γzx
γzy
γzz
νs
νx
νy
νz
1.0
0.5
0.0
−0.5
−1.0

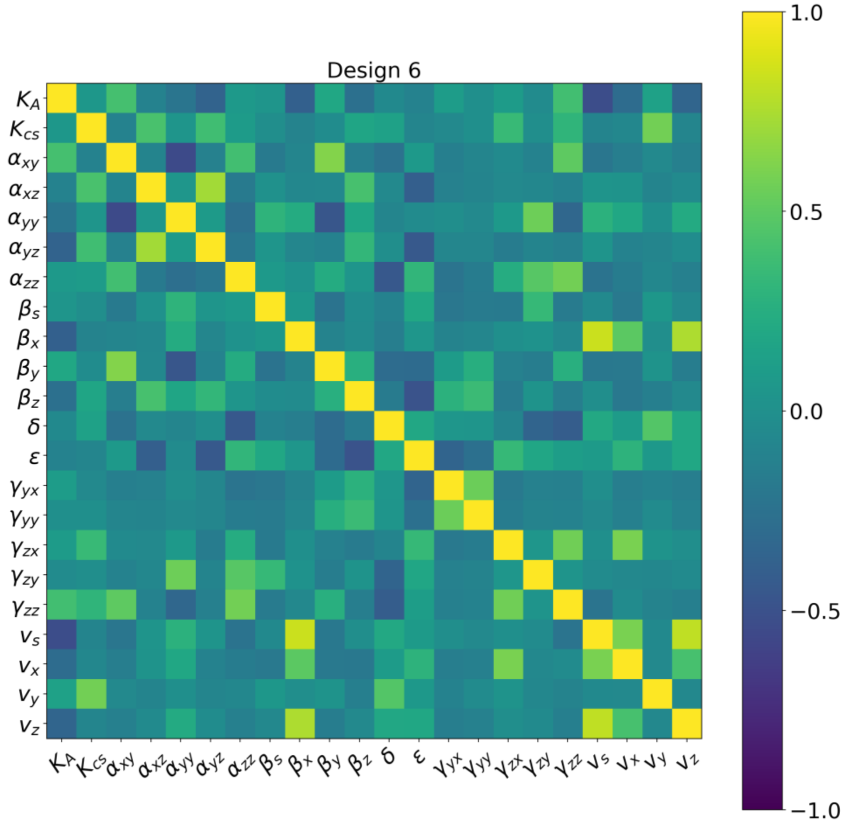
Design 6
KA
Kcs
αxy
αxz
αyy
αyz
αzz
βs
βx
βy
βz
δ
ε
γyx
γyy
γzx
γzy
γzz
νs
νx
νy
νz
1.0
0.5
0.0
−0.5
−1.0

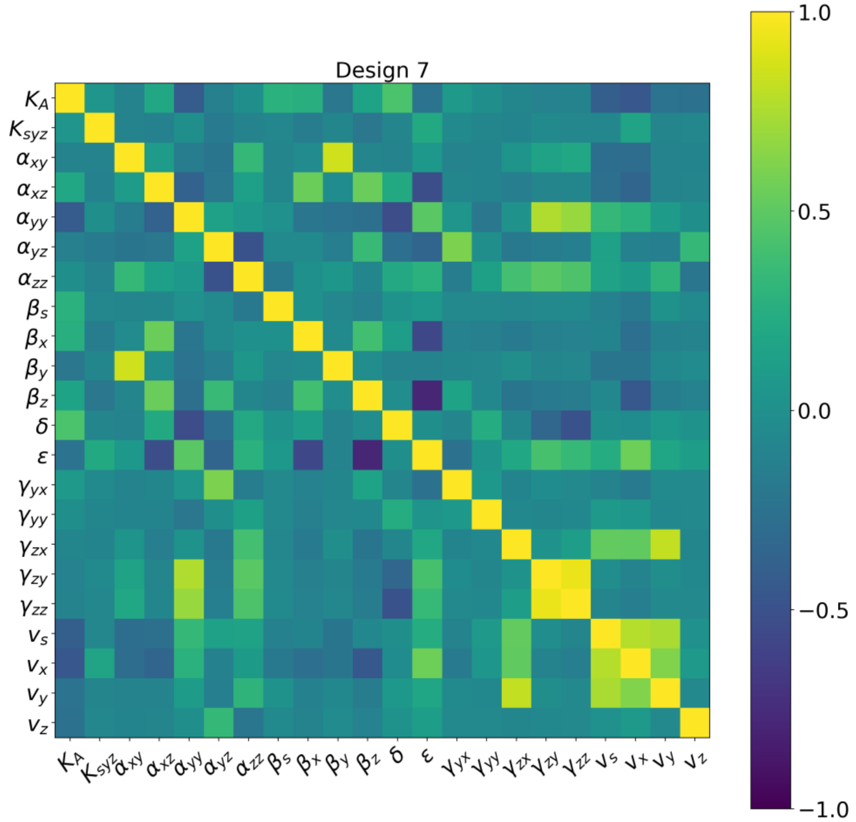
Design 7
K_A
K_syz
α_xy
α_xz
α_yy
α_yz
α_zz
β_s
β_x
β_y
β_z
δ
ε
γ_yx
γ_yy
γ_zx
γ_zy
γ_zz
ν_s
ν_x
ν_y
ν_z
1.0
0.5
0.0
−0.5
−1.0

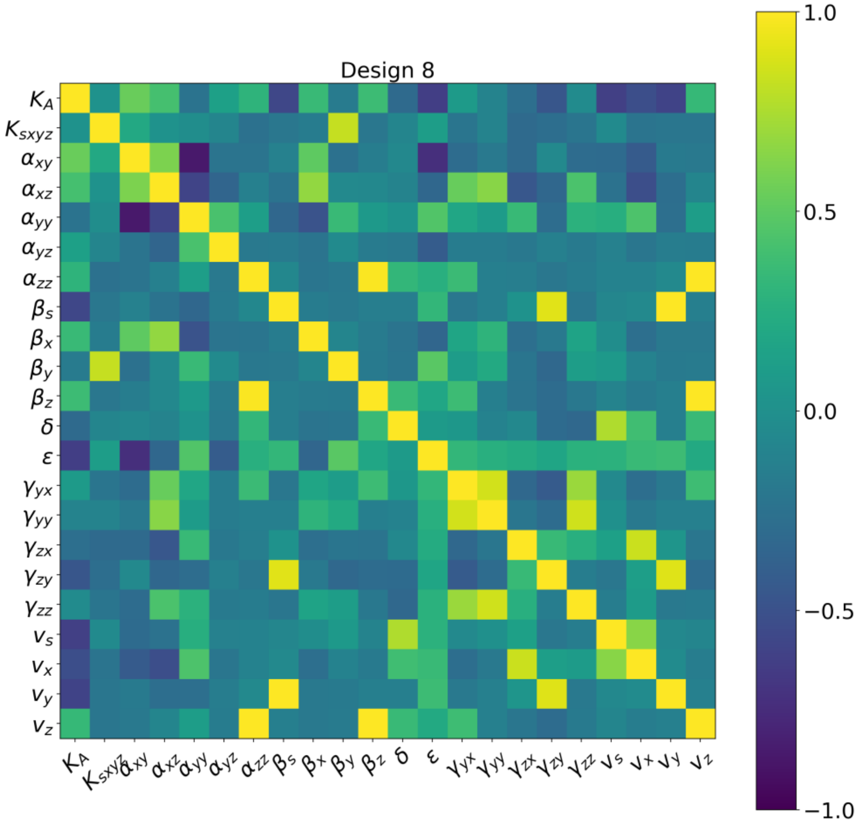
Design 8
K_A
K_sxyz
α_xy
α_xz
α_yy
α_yz
α_zz
β_s
β_x
β_y
β_z
δ
ε
γ_yx
γ_yy
γ_zx
γ_zy
γ_zz
ν_s
ν_x
ν_y
ν_z
1.0
0.5
0.0
−0.5
−1.0

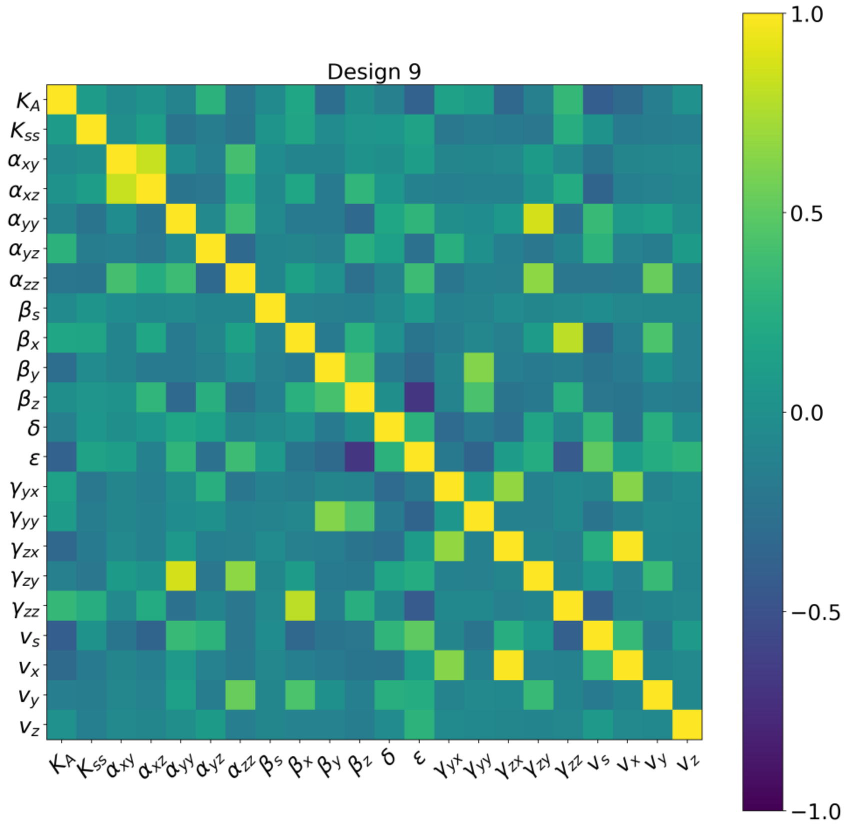

Supplementary Table 3. Set of combinations of two parameters that were highly correlated (absolute value of correlation coefficient ≥ 0.5) in three or more of the model designs.

		Designs	Positive correlation	Negative correlation
α_{yy}	γ_{zy}	3, 4, 5, 6, 7, 9	6	0
α_{xy}	α_{xz}	3, 5, 8, 9	4	0
v_s	v_x	3, 6, 7, 8	4	0
K_A	v_s	3, 4, 6, 8	0	4
γ_{zx}	v_x	6, 7, 8, 9	4	0
α_{xy}	β_y	3, 5, 6, 7	4	0
α_{xz}	β_z	3, 5, 7	3	0
β_z	ε	3, 7, 9	0	3

7 Spread of identified limit cycles across the parameter space

To indicate the unlikeliness of the connectivity between the identified parameter sets that result in limit cycles, boxplots of the parameter values in these parameter sets for designs *3–9* were generated (**Supplementary Figure 26**). For all designs most parameter values cover multiple orders of magnitude. We do note that the logarithmic scales on the lower end, e.g. –3 through –9, should not be given too much weight, since this may simply indicate that the parameter value is so small that would not affect the model output. However, even discounting the lower range, the parameter values in the limit cycles still cover a wide range of values.

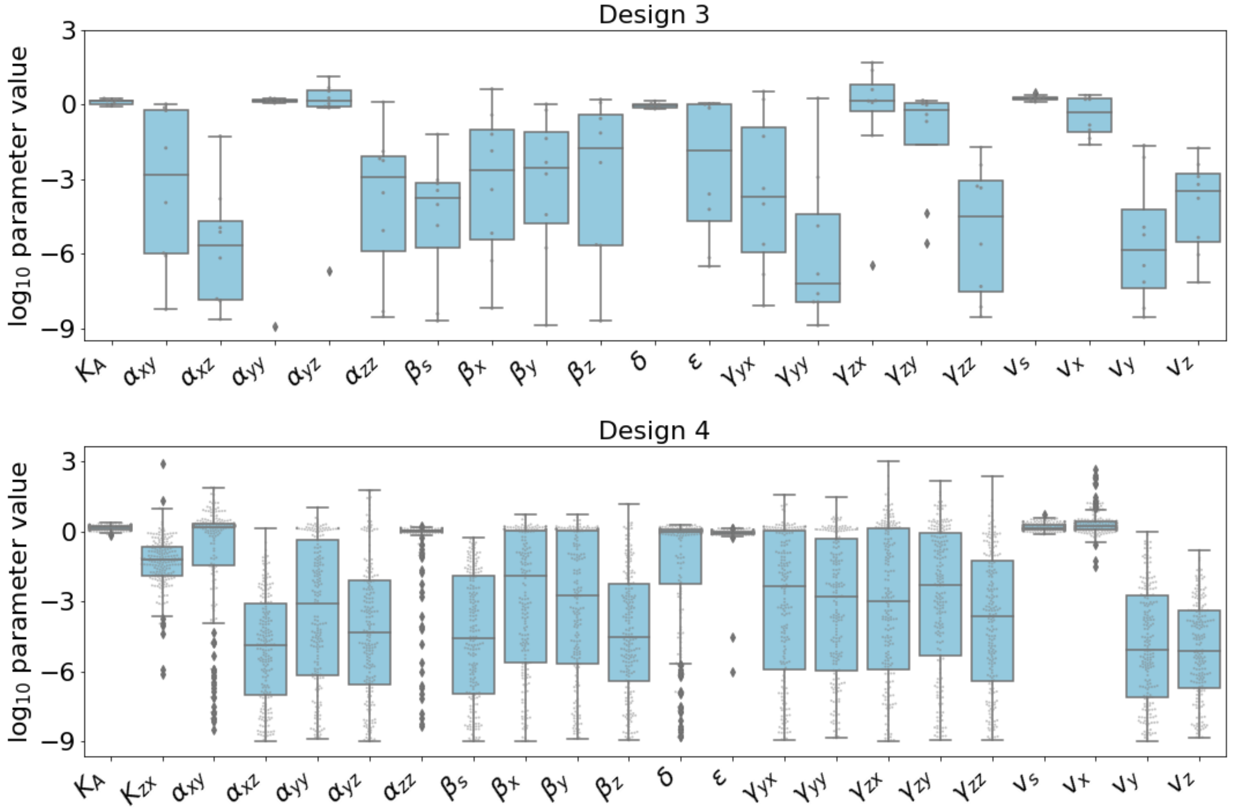

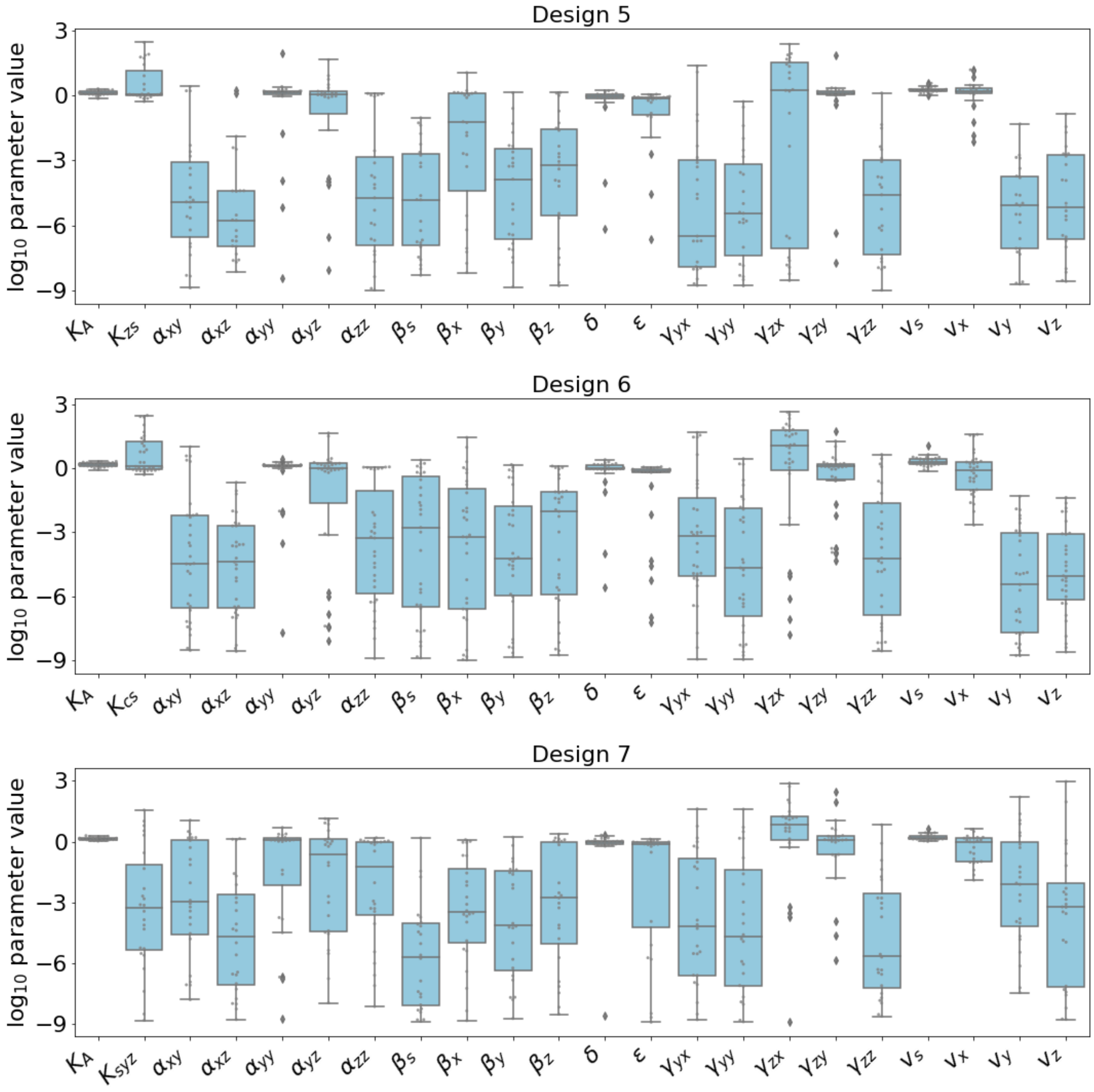
Design 5
Design 6
Design 7
log10 parameter value
3
0
−3
−6
−9
KA
Kzs
Kcs
Ksyz
αxy
αxz
αyy
αyz
αzz
βs
βx
βy
βz
δ
ε
γyx
γyy
γzx
γzy
γzz
νs
νx
νy
νz

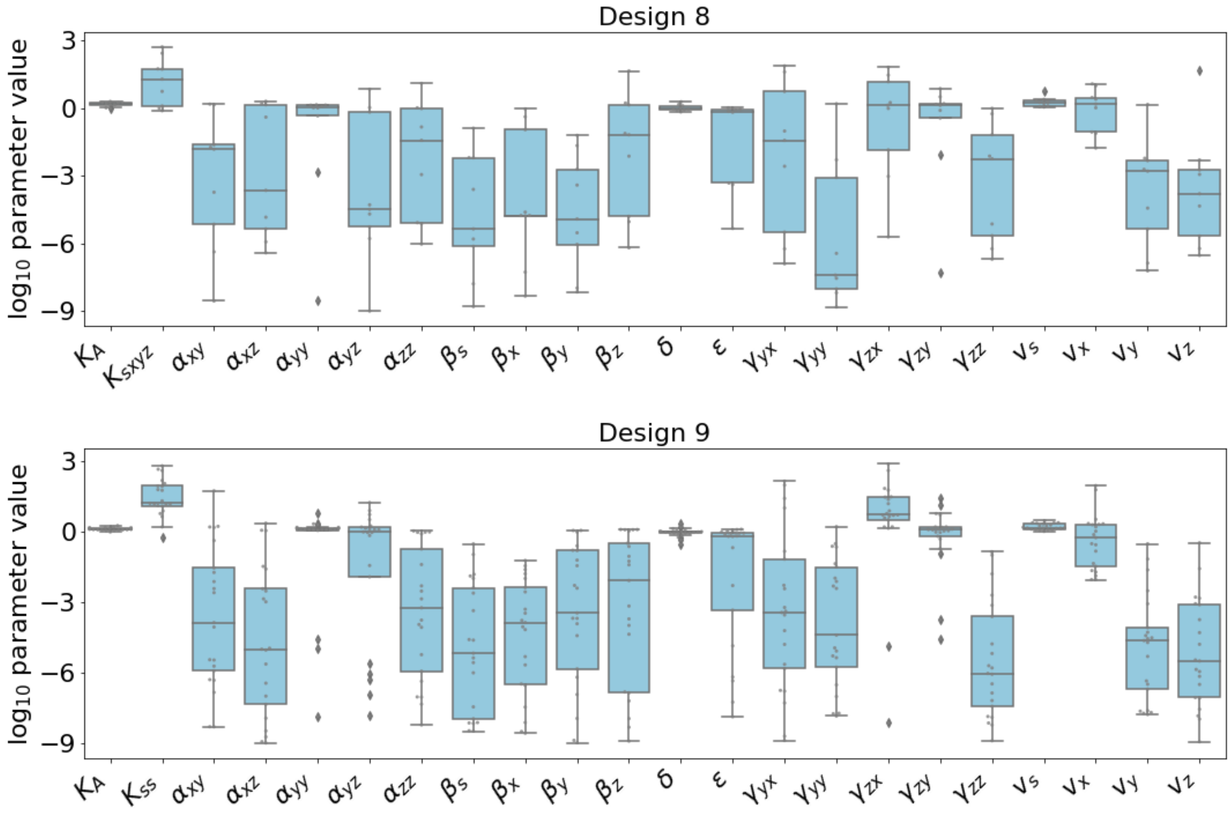

Supplementary Figure 26. Boxplots of the parameter values in the limit cycles identified for designs *3–9* on log10 scale. The entire allowable range in our parameter sampling [10^3, 10^(-9)] is shown. The box edges indicate the 25th and 75th percentile respectively and the black line within the box indicates the median of the parameter values. The actually sampled parameter values are indicates by grey dots.

8 Limit cycles in designs *3–9* differ in oscillation properties (period and amplitude)

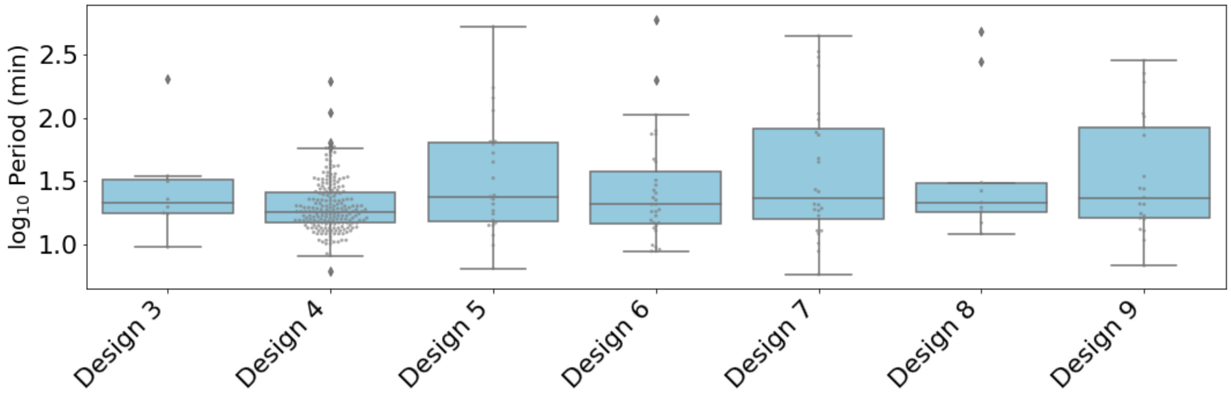

Supplementary Figure 27. Boxplots of the logarithm of the period of the limit cycles identified for designs *3–9*. The range covers periods from several minutes to several hundred minutes.

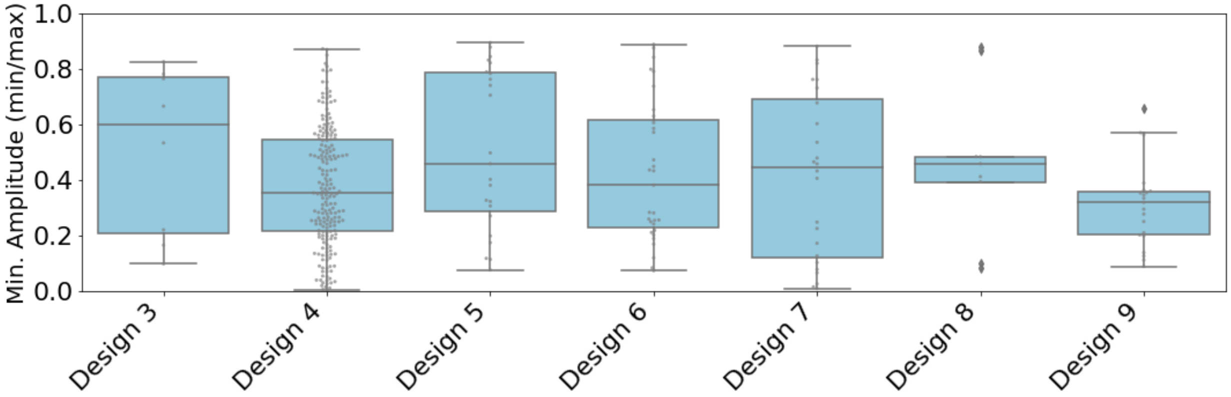

Supplementary Figure 28. Boxplots for designs *3–9* of the "minimum oscillation amplitude". We defined this as the maximum taken across the four model species of the ratio between the minimum and maximum concentrations attained within the limit cycle time course. This quantity therefore measures the relative amplitude of the oscillations as a fraction of the maximal concentration for each species. Taking the maximum across species results into a measure of the "minimum amplitude size" of the individual oscillations in the limit cycle. The smaller the value the bigger the oscillations are, relatively. A value of 1 indicates lack of oscillations, whereas a value of 0 indicates that the concentration of each species reaches zero during each cell cycle. We required each limit cycle to have a maximal ratio of 0.9.

References

[1] Barberis, M., Linke, C., Adrover, M. À., González-Novo, A., Lehrach, H., et al. Sic1 plays a role in timing and oscillatory behaviour of B-type cyclins. *Biotechnol. Adv.* **30**, 108–130 (2012).

[2] Linke, C., Chasapi, A., González-Novo, A., Al Sawad, I., Tognetti, S., et al. A Clb/Cdk1-mediated regulation of Fkh2 synchronizes *CLB* expression in the budding yeast cell cycle. *NPJ Syst. Biol. Appl.* **3**, 7 (2017).

[3] Rossi, R. L., Zinzalla, V., Mastriani, A., Vanoni, M. & Alberghina, L. Subcellular localization of the cyclin dependent kinase inhibitor Sic1 is modulated by the carbon source in budding yeast. *Cell Cycle* **4**, 1798–1807 (2005).

[4] Hoops, S., Sahle, S., Gauges, R., Lee, C., Pahle, J. et al. COPASI—a COmplex PAthway Simulator. *Bioinformatics* **22**, 3067–3074 (2006).

[5] Dhooge, A., Govaerts, W. & Kuznetsov, Y. A. MATCONT. *ACM Trans. Math. Softw.* **29**, 141–164 (2003).

[6] Dhooge, A., Govaerts, W., Kuznetsov, Y. A., Meijer, H. G. E. & Sautois, B. New features of the software MatCont for bifurcation analysis of dynamical systems. *Math. Comput. Model. Dyn. Syst.* **14**, 147–175 (2008).

[7] Ermentrout, B. *Simulating, Analyzing, and Animating Dynamical Systems: A Guide to XPPAUT for Researchers and Students* (Society for Industrial and Applied Mathematics, SIAM, Press, Philadelphia, 2002).

[8] Kholodenko, B. N., Demin, O. V. & Westerhoff, H. V. Control Analysis of Periodic Phenomena in Biological Systems. *J. Phys. Chem. B* **101**, 2070–2081 (1997).

[9] Reijenga, K. A., Westerhoff, H. V., Kholodenko, B. N. & Snoep, J. L. Control analysis for autonomously oscillating biochemical networks. *Biophys. J.* **82**, 99–108 (2002).

[10] Fell, D. A. Metabolic control analysis: a survey of its theoretical and experimental development. *Biochem. J.* **286**, 313–330 (1992).

[11] Westerhoff, H. V. Signalling control strength. *J. Theor. Biol.* **252**, 555–567 (2008).

[12] Domijan, M., Brown, P. E., Shulgin, B. V. & Rand, D. A. PeTTSy: a computational tool for perturbation analysis of complex systems biology models. *BMC Bioinformatics* **17**, 124 (2016).

[13] Cross, F. R. Two redundant oscillatory mechanisms in the yeast cell cycle. *Dev. Cell.* **4**, 741–752 (2003).

[14] Wäsch, R. & Cross, F. R. APC-dependent proteolysis of the mitotic cyclin Clb2 is essential for mitotic exit. *Nature* **418**, 556–562 (2002).

[15] Teusink, B., Bakker, B. M. & Westerhoff, H. V. Control of frequency and amplitudes is shared by all enzymes in three models for yeast glycolytic oscillations. *Biochim. Biophys. Acta* **1275**, 204–212 (1996).

[16] Fiedler, D., Braberg, H., Mehta, M., Chechik, G., Cagney, G. et al. Functional Organization of the S. cerevisiae Phosphorylation Network. *Cell* **136**, 952–963 (2009).

[17] Amon, A., Tyers, M., Futcher, B. & Nasmyth, K. Mechanisms that help the yeast cell cycle clock tick: G2 cyclins transcriptionally activate G2 cyclins and repress G1 cyclins. *Cell* **74**, 993–1007 (1993).

[18] Skotheim, J. M., Di Talia, S., Siggia, E. D. & Cross F. R. Positive feedback of G1 cyclins ensures coherent cell cycle entry. *Nature* **454**, 291–296 (2008).

[19] Verma, R., Annan, R. S., Huddleston, M. J., Carr, S. A., Reynard, G. et al. Phosphorylation of Sic1p by G1 Cdk required for its degradation and entry into S phase. *Science* **278**, 455–460 (1997).

[20] Moll, T., Tebb, G., Surana, U., Robitsch, H. & Nasmyth, K. The role of phosphorylation and the CDC28 protein kinase in cell cycle-regulated nuclear import of the S.cerevisiae transcription factor SWI5. *Cell* **66**, 743–758 (1991).

[21] Visintin, R., Craig, K., Hwang, E. S., Prinz, S., Tyers, M. et al. The phosphatase Cdc14 triggers mitotic exit by reversal of Cdk-dependent phosphorylation. *Mol. Cell* **2**, 709–718 (1998).

[22] Rahi, S. J., Pecani, K., Ondracka, A., Oikonomou, C. & Cross, F. R. The CDK-APC/C Oscillator Predominantly Entrains Periodic Cell-Cycle Transcription. *Cell* **165** 475–487 (2016).

[23] Savageau, M. A., Coelho, P. M., Fasani, R. A., Tolla, D. A. & Salvador, A. Phenotypes and tolerances in the design space of biochemical systems. *Proc. Natl. Acad. Sci. U S A* **106**, 6435–6440 (2009).

[24] Lomnitz, J. G. & Savageau, M. A. Phenotypic deconstruction of gene circuitry. *Chaos* **23**, 025108 (2013).

[25] Lomnitz, J. G. & Savageau, M. A. Strategy revealing phenotypic differences among synthetic oscillator designs. *ACS Synth. Biol.* **3**, 686–701 (2014).

[26] Lomnitz, J. G. & Savageau, M.A. Elucidating the genotype–phenotype map by automatic enumeration and analysis of the phenotypic repertoire. *NPJ Syst. Biol. Appl.* **1**, pii:15003 (2015).

[27] Lomnitz, J. G. & Savageau, M. A. Design Space Toolbox V2: Automated Software Enabling a Novel Phenotype-Centric Modeling Strategy for Natural and Synthetic Biological Systems. *Front. Genet.* **7**, 118 (2016).

[28] Hilbert, D. Mathematical Problems. *Bull. Amer. Math. Soc.* **8**, 437–479 (1902).

[29] Savageau, M. A. & Voit, E. O. Recasting nonlinear differential equations as S-systems: a canonical nonlinear form. *Math. Biosci.* **87**, 83–115 (1987).

[30] Voit, E. O. Biochemical Systems Theory: A Review. *ISRN Biomath*. **2013**, 897658 (2013).

[31] Savageau, M. A. Introduction to S-systems and the underlying power-law formalism. *Mathl Comput. Modelling* **11**, 546–551 (1988).

[32] Hollenhorst, P. C., Bose, M. E., Mielke, M. R., Müller, U. & Fox, C. A. Forkhead genes in transcriptional silencing, cell morphology and the cell cycle. Overlapping and distinct functions for FKH1 and FKH2 in Saccharomyces cerevisiae. *Genetics* **154**, 1533–1548 (2000).

[33] Pic-Taylor, A., Darieva, Z., Morgan, B. A. & Sharrocks, A. D. Regulation of cell cycle-specific gene expression through cyclin-dependent kinase-mediated phosphorylation of the forkhead transcription factor Fkh2p. *Mol. Cell. Biol.* **24**, 10036–10046 (2004).

[34] Ubersax, J. A., Woodbury, E. L., Quang, P. N., Paraz, M., Blethrow, J. D. et al. Targets of the cyclin-dependent kinase Cdk1. *Nature* **425**, 859–864 (2003).

[35] Yeong, F. M., Lim, H. H., Wang, Y. & Surana, U. Early expressed Clb proteins allow accumulation of mitotic cyclin by inactivating proteolytic machinery during S phase. *Mol. Cell. Biol.* **21**, 5071–5081 (2001).

[36] Kumar, R., Reynolds, D. M., Shevchenko, A., Shevchenko, A., Goldstone, S. D. et al. Forkhead transcription factors, Fkh1p and Fkh2p, collaborate with Mcm1p to control transcription required for M-phase. *Curr. Biol.* **10**, 896–906 (2000).

[37] Reynolds, D., Shi, B. J., McLean, C., Katsis, F., Kemp, B. et al. Recruitment of Thr 319-phosphorylated Ndd1p to the FHA domain of Fkh2p requires Clb kinase activity: a mechanism for CLB cluster gene activation. *Genes Dev.* **17**, 1789–1802 (2003).

[38] Rudner, A. D. & Murray, A. W. Phosphorylation by Cdc28 activates the Cdc20-dependent activity of the anaphase-promoting complex. *J. Cell Biol.* **149**, 1377–1390 (2000).

[39] Shirayama, M., Tóth, A., Gálová, M. & Nasmyth, K. APC(Cdc20) promotes exit from mitosis by destroying the anaphase inhibitor Pds1 and cyclin Clb5. *Nature* **402**, 203–207 (1999).

[40] Zachariae, W., Schwab, M., Nasmyth, K. & Seufert, W. Control of cyclin ubiquitination by CDK-regulated binding of Hct1 to the anaphase promoting complex. *Science* **282**, 1721–1724 (1998).

[41] Nash, P., Tang, X., Orlicky, S., Chen, Q., Gertler, F. B. et al. Multisite phosphorylation of a CDK inhibitor sets a threshold for the onset of DNA replication. *Nature* **414**, 514–521 (2001).
